# CO_2_ Activation over Nanoshaped CeO_2_ Decorated with Nickel for Low-Temperature Methane Dry Reforming

**DOI:** 10.1021/acsami.2c05221

**Published:** 2022-07-08

**Authors:** Kristijan Lorber, Janez Zavašnik, Iztok Arčon, Matej Huš, Janvit Teržan, Blaž Likozar, Petar Djinović

**Affiliations:** † National Institute of Chemistry, Hajdrihova 19, 1000 Ljubljana, Slovenia; ‡ University of Nova Gorica, Vipavska 13, SI-5000 Nova Gorica, Slovenia; § Jožef Stefan Institute, Jamova cesta 39, SI-1000 Ljubljana, Slovenia; ∥ Association for Technical Culture (ZOTKS), Zaloška 65, 1000 Ljubljana, Slovenia

**Keywords:** surface carbonates, *in situ* characterization, spectator species, CeO_2_ nanoshapes, CO_2_ activation

## Abstract

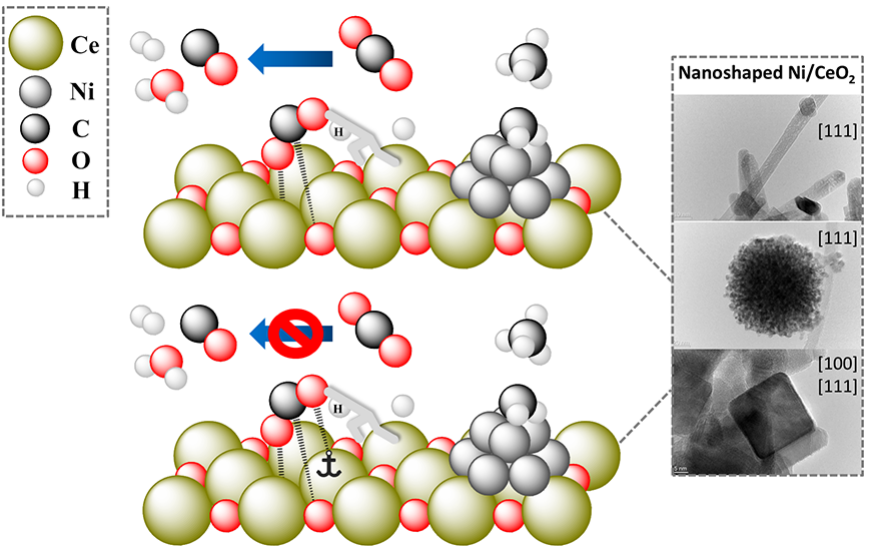

Dry reforming of methane (DRM) is a promising way to convert methane and carbon dioxide into H_2_ and CO (syngas). CeO_2_ nanorods, nanocubes, and nanospheres were decorated with 1–4 wt % Ni. The materials were structurally characterized using TEM and *in situ* XANES/EXAFS. The CO_2_ activation was analyzed by DFT and temperature-programmed techniques combined with MS-DRIFTS. Synthesized CeO_2_ morphologies expose {111} and {100} terminating facets, varying the strength of the CO_2_ interaction and redox properties, which influence the CO_2_ activation. Temperature-programmed CO_2_ DRIFTS analysis revealed that under hydrogen-lean conditions mono- and bidentate carbonates are hydrogenated to formate intermediates, which decompose to H_2_O and CO. In excess hydrogen, methane is the preferred reaction product. The CeO_2_ cubes favor the formation of a polydentate carbonate species, which is an inert spectator during DRM at 500 °C. Polydentate covers a considerable fraction of ceria’s surface, resulting in less-abundant surface sites for CO_2_ dissociation.

## 1 Introduction

In recent decades, economic growth has led to an increased demand for energy, which is the driving force for human welfare.^1^ The majority (>90%) of fuels and energy is produced by combusting fossil fuels, causing elevated CO_2_ emission,^2^ which has a major impact on global warming. Lately, many legislative and research efforts have been dedicated to the large-scale utilization of CO_2_ for the production of fuels and chemicals or enhanced biological and technological utilization.^3^ The main problem of CO_2_ utilization is that the molecule is very stable (Δ*G*_298 K_ = −394 kJ/mol) and requires about 530 kJ/mol for the C=O bond to dissociate. Methane is an abundant hydrocarbon source that has an even higher greenhouse effect than CO_2_. One of the promising methods for large-scale methane and CO_2_ utilization is the dry reforming of methane (DRM, eq 1), which converts CO_2_ and CH_4_ into syngas (H_2_ and CO) with a H_2_/CO ratio of <1. The CO-rich syngas produced via DRM is suitable for the synthesis of dimethyl ether or long-chain hydrocarbons via the Fischer–Tropsch process.^4^

There are several drawbacks related to catalytic DRM: difficult activation of both CH_4_ and CO_2_ and a reaction that is highly endothermic and thermodynamically limited, which requires high temperatures for substantial CH_4_ and CO_2_ conversion.
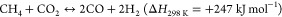
1
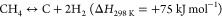
2
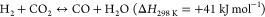
3
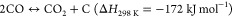
4During the DRM reaction, several side reactions occur, such as methane cracking, the reverse water–gas shift (RWGS), and the Boudouard reaction (eqs 2–4)). These favor carbon accumulation and catalyst deactivation and also impact the H_2_/CO ratio by lowering the H_2_ selectivity.^5^

Nickel is a promising metal for catalyzing the DRM because of its low cost, abundance, and high activity. The main disadvantage of Ni is its low carbon resistance,^6^ which strongly depends on the catalyst support and nickel ensemble size.^7,8^ Different threshold values for the nickel particle size are reported, above which carbon accumulation is initiated: about 5 nm for silica,^8^ 10 nm on γ-Al_2_O_3_^9^ and 20 nm for CeZrO_2_.^7^ The carbon accumulation on nickel is influenced by the metal–support interactions. Specifically in the case of ceria, a sufficient supply of oxygen species from the support has to be ensured to gasify the carbon precursors accumulating on the nickel surface.^7^ The oxygen mobility in ceria and consequently its carbon gasification efficiency can be adjusted by doping^10,11^ or nanoparticle engineering to preferentially expose certain facets, namely, {100}, {110}, and {111}.^12,13^ These facets are characterized by different energies of oxygen vacancy formation (1.96, 1.06, and 1.35 eV on {111}, {110}, and {100}, respectively^14^), resulting in different reactivity and mobility of surface oxygen.

In recent years, a lot of research has focused on understanding the structure–activity relationships of nanoshaped ceria in different reactions.^12^ Over different CeO_2_ nanoshapes, Li et al.^15^ observed a beneficial effect of CeO_2_ {100} and {110} crystal planes on activity for CO oxidation, compared to CeO_2_ {111}. A similar activity dependency trend was reported by Trovarelli et al.^16^ for the soot oxidation reaction. Zhang et al.^17^ studied the DRM reaction over Ni supported on CeO_2_ nanorods and nanopolyhedra. They reported a higher catalytic activity and carbon accumulation resistance of nanorod-based catalysts, which they attributed to more abundant reactive oxygen species on CeO_2_ {100} and {110} crystal planes than on {111}.

On the basis of UV/vis, Raman, and XPS analyses, Wei et al.^18^ reported a decreasing abundance of oxygen vacancies as well as oxygen mobility in ceria in the following order: rods > octahedrons > cubes > particles. The catalytic activity for DRM over Ni/CeO_2_ catalysts over the mentioned morphologies decreased in the same order. In addition, both DFT and experiments suggest that oxygen-assisted methane activation could be used to accelerate the C–H bond activation.^19^ Consequently, the ability to optimize ceria for accelerating CO_2_ activation and dissociation and initiating oxygen spillover could be exploited to accelerate the DRM reaction and minimize carbon accumulation. Although ceria is known as a basic support,^20^ its basic/acidic properties can vary depending on the exposed facet.^21^ Remarkably active catalysts for the low-temperature methane dry reforming reaction are of great academic and industrial interest, which would increase the feasibility of decentralized natural gas valorization. A detailed structure-dependent understanding of CO_2_ activation pathways on such highly active catalytic surfaces is important not only for avoiding carbon accumulation in DRM and improving stability but also for accelerating CO_2_ reduction to value-added chemicals such as CO, CH_4_, and methanol.

In this work, 1–4 wt % nickel was deposited on CeO_2_ rods, cubes, and spheres, which in the DRM reaction exhibited notably different catalytic activity as well as the dynamics of carbon accumulation. Using temperature-programmed DRIFT-MS analyses complemented with DFT, we analyzed the CO_2_ activation pathways in the absence and presence of hydrogen over the particularly promising 2Ni/CeO_2_ nanorod catalyst.

## 2 Material and Methods

### 2.1 Synthesis of Catalysts

The nanorod (CeO_2_–R)- and nanocube (CeO_2_–C)-shaped CeO_2_ was synthesized as reported by Zabilskiy et al.^22^ In a typical synthesis, 58.8 g of NaOH (Merck, purity 99%) was dissolved in 140 mL of ultrapure water. A separate solution containing 4.9 g of Ce(NO_3_)_3_·6H_2_O (Sigma-Aldrich, purity 99%) was dissolved in 84 mL of ultrapure water. Both solutions were mixed for 0.5 h using a magnetic stirrer and transferred into Teflon-lined stainless steel autoclaves (∼35 mL volume). The autoclaves were heated to 100 and 180 °C for 24 h to stimulate the formation of ceria nanorods and cubes, respectively. The precipitate was separated from the supernatant by repetitive washing and centrifugation (7500 rpm for 90 s) using ultrapure water and finally absolute ethanol until complete sodium removal occurred. To lower the possibility of CeO_2_ nanorods breaking, the CeO_2_–R precursor was freeze-dried and the CeO_2_–C precursor was dried overnight in a laboratory dryer in air at 70 °C. The dried precursors were calcined in a static air atmosphere at 500 °C for 4 h with a heating ramp of 5 °C/min using a chamber furnace (Nabertherm P330).

The CeO_2_ spheres (CeO_2_–S) were synthesized using a modified protocol reported by Yan et al.^23^ and cetyltrimethylammonium bromide (CTAB, Alfa Aesar, purity 98%) as the structure-directing agent. In a typical synthesis, 0.91 g of CTAB and 2.18 g of Ce(NO_3_)_3_·6H_2_O (Sigma-Aldrich, purity 99%) were dissolved in 10 mL of absolute EtOH and stirred for 1 h at room temperature in a 50 mL glass beaker covered with Parafilm. The solution was transferred into a laboratory drier and aged in two steps: at 40 °C for 48 h with a controlled relative humidity of 25%. For the formation of ceria spheres, maintaining the mentioned relative humidity is crucial. In the second step, the solution was held at 100 °C for 48 h, independent of the relative humidity. After the appointed aging, CTAB was removed from the precipitate by extraction in absolute EtOH at 45 °C for 24 h. The collected powder was calcined in static air at 500 °C for 4 h with a heating ramp of 1 °C/min in a chamber furnace (Nabertherm P330).

When depositing a nominal 2 wt % nickel content, 100 mg of Ni(NO_3_)_2_·6H_2_O (99%, Merck) was dissolved in 40 mL of ultrapure water. After stirring for 15 min, 1 g of a powdered CeO_2_ support was added and stirred for an additional 15 min. Afterward, a 2.5 wt % aqueous ammonia solution was added dropwise until the pH reached 7.5. The beaker was covered with Parafilm and stirred for an additional 2 h. After the latter, the pH value of the suspension was adjusted to 9 with a 25 wt % aqueous ammonia solution and stirred for an additional 15 min. The final product was filtered, dried at 70 °C overnight, and calcined at 500 °C for 4 h with a heating ramp of 5 °C/min.

The synthesized catalysts are denoted as *x*Ni–R, *x*Ni–C, and *x*Ni–S for nanorods, nanocubes, and nanospheres, respectively, where *x* represents the nominal nickel content. The actual Ni content (analyzed using ICP-OES) equaled 1.95 (2Ni–R), 1.80 (2Ni–C), and 1.85 wt % (2Ni–S).

### 2.2 Characterization and Catalytic Testing

#### 2.2.1 Transmission Electron Microscopy

The phase composition and crystal structure of the samples were analyzed by transmission electron microscopy (TEM, JEM-2010F, Jeol Inc.) operating at 200 kV and equipped with a slow-scan CCD camera (Orius SC-1000, Gatan). The powdered samples were dispersed in EtOH and sonicated to prevent agglomeration and then transferred onto Cu-supported amorphous carbon lacey grids. Raw image data was processed with Digital Micrograph software (GMS3, Gatan), and selected-area electron diffraction data was simulated by electron microscopy software Java Version (JEMS 4.9).

#### 2.2.2 BET Specific Surface Area and Porosity

The BET specific surface area, total pore volume, and pore size distribution were determined by N_2_ physisorption at −196 °C (Micromeritics, model TriStar II 3020). Before the analysis, the samples were degassed in a flow of N_2_ (purity 6.0, Linde) for 1 h at 90 °C, followed by 4 h at 300 °C. The total pore volume and pore size distribution values were calculated with the BJH (Barrett–Joyner–Halenda) method from the desorption branch of the isotherms.

#### 2.2.3 X-ray Diffraction

The PANalytical Empyrean diffractometer using Bragg–Brentano geometry and Cu Kα1 radiation was used for the characterization of the crystalline phases. XRD patterns were recorded in the 2θ range from 10 to 80°, with a measurement increment of 0.034° and a step time of 100 s.

#### 2.2.4 Temperature-Programmed Analyses

The H_2_-TPR and CO_2_-TPD analyses were performed to (i) qualitatively and quantitatively assess the amount of removable oxygen as a function of ceria shape and (ii) qualitatively and quantitatively assess the number of CO_2_ adsorption sites. Powdered samples (∼60 mg) were positioned inside a U-shaped quartz reactor on a flock of quartz wool (Micromeritics Autochem 2920 apparatus). The analytical procedure is shown in Figure S1A. The temperature range of the H_2_-TPR analysis (25–550 °C) was selected to cover the range of temperatures, probed by the catalytic tests (see below).

The dynamic structure of CO_2_ bound to CeO_2_–R and 2Ni–R was analyzed by temperature-programmed *in situ* DRIFTS-MS (DiffusIR cell from PIKE Technologies) on reduced and oxidized samples. Evolved gases were continuously analyzed by mass spectrometry. Three possible scenarios were analyzed: (A) CO_2_ activation in the absence of hydrogen, (B) CO_2_ activation with hydrogen species adsorbed on the catalyst, mimicked by formic acid (FA) as the probe molecule, and (C) CO_2_ activation in excess gaseous hydrogen. See the Supporting Information and Figure S1 for more details.

#### 2.2.5 *In Situ* Ni K-Edge XANES and EXAFS

The *in situ* Ni K-edge XANES and EXAFS spectra were measured in transmission mode on the 2Ni–R, 2Ni–C, 2Ni–S, and 4Ni–R catalysts, first at RT in air, then after reduction in a 5% H_2_/N_2_ stream (flow rate of 15 mL/min at 1 bar) for 60 min at 400 °C, and during the DRM reaction. For the latter, the catalyst was kept for 30 min at 400, 500, and 550 °C in a CH_4_/CO_2_ atmosphere (50% CH_4_, 50% CO_2_, flow rate 20 mL/min each).

We measured the XAS scans on different spots of the sample pellet to avoid potential measurement errors caused by radiation damage to the catalyst. The exact energy calibration was established with an absorption measurement on a 5-μm-thick Ni foil placed between the second and third ionization detectors. The absolute energy reproducibility of the measured spectra was ±0.02 eV.

The quantitative analysis of XANES and EXAFS spectra was performed with the Demeter program package^24^ in combination with FEFF6 program code^25^ for the *ab initio* calculation of photoelectron scattering paths. More details on XAS analyses are shown in the Supporting Information.

#### 2.2.6 DFT Calculations

Electronic structure calculations were performed using VASP 5.4.1.^26−28^ The periodicity of the crystals was accounted for using the plane-wave formalism of the density functional theory with the projector augmented wave method.^29,30^ The energy cutoff of 500 eV was tested and found to suffice for well-converged results (<1 meV). The geometric optimizations were performed at the GGA+U level with the Perdew–Burke–Ernzerhof (PBE) functional^31^ in the single effect parameter (*U*–*J*) approximation, as proposed by Dudarev et al.^32^ The on-site Coulomb interaction of localized electrons was applied to Ce 4f orbitals only with *U*–*J* = 4.5 eV, as done for CeO_2_ by Penschke and Paier.^33^ The 4.5 eV value for ceria in PBE+U had also been calculated self-consistently by Fabris et al.^34^ and used as such.^35^ The Grimme D3 correction for van der Waals interactions was employed.^36^

For the bulk calculations, a 8 × 8 × 8 Monkhorst–Pack *k* mesh was used. For the *p*(2 × 2) slabs, a 2 × 2 × 1 mesh was found to suffice for well-converged results. For molecules in vacuum, a box with dimensions of 20 × 21 × 22 Å^3^ was used with gamma-point meshing.

During the GGA+U relaxations, the threshold was set at 0.02 eV/Å. All geometries were confirmed to represent local minima by performing a vibrational analysis, where no imaginary frequencies were found. Standard dipole corrections on the *z* axis were used to describe the slabs.^37,38^ Spin polarization was used when required. Activation barriers for the diffusion of oxygen vacancies were calculated with the climbing-image nudged elastic band method with seven images.^39,40^

The adsorption energies were calculated as *E*_ads_ = *E*_adsorbed_ – *E*_slab_ – *E*_gaseous_, where *E*_adsorbed_ denotes the energy of the slab with the adsorbed adsorbate, *E*_slab_ is the energy of the empty relaxed slab, and *E*_gaseous_ is the energy of the relaxed adsorbate in vacuum. The experimentally determined entropy of gasesous CO_2_ is 213.8 J/(mol K).^41^ During the adsorption, CO_2_ loses approximately one-third of its entopy, yielding 143 J/(mol K).^42^ The temperature of desorption is estimated from Δ*G*(*T*)_ads_ = 0, where *G* = *H* – *TS* = *E*_ads_ – *TS* + *PV*.

For the CeO_2_(111) and CeO_2_(110) structure, 12-layer slabs (four O–Ce–O trilayers) with the bottom 6 layers fixed in their bulk positions were constructed. For CeO_2_(100), an eight-layer slab was used with the bottom four layers fixed. However, because CeO_2_(100) is a polar structure,^43^ the stable geometry was compensated for by moving half of the top oxygen atoms to the bottom of the slab, effectively making a nine-layer slab.^44^ For precise electronic energy calculations on previously relaxed structures, the tetrahedron method with Blöchl corrections was used for smearing.^45^

#### 2.2.7 Catalytic Tests

In preliminary catalytic tests (Figure S3), a powdered sample (20 mg) was activated in 5% H_2_/N_2_ (15 mL/min) at 400 °C for 1 h. After pretreatment, the activity was analyzed in 50 °C increments between 400 and 550 °C in an equimolar flow of CH_4_ and CO_2_ (30 mL/min each) in a tubular quartz reactor (5 mm i.d.) at atmospheric pressure. For catalytic activity and stability tests, catalysts were activated for 1 h at 500 °C and the catalytic activity was investigated in 20 °C decrements or isothermally at 500 °C, respectively. The reaction products were analyzed by gas chromatography (Agilent490, equipped with Poraplot U and MS5A capillary columns). To ensure that the intrinsic activity is reported, operation in the kinetic regime was experimentally confirmed (Figure S2). The amount of carbon accumulated during the DRM reaction was quantified using a CHNS elemental analyzer (PerkinElmer, model 2400).

To evaluate the reactivity of the carbon which accumulated during DRM, methane cracking, and the Boudouard reaction, the 2Ni–R, 2Ni–C, and 2Ni–S samples were analyzed. The catalysts were first activated in 5% H_2_/Ar for 1 h at 500 °C, followed by exposure to either equimolar CH_4_–CO_2_ (WHSV = 180 L/g_cat_·h), pure CH_4_, or a CO flow (WHSV = 120 L/g_cat_·h), for 1 h. The reactivity of carbon was analyzed using a TGA-TPO technique (PerkinElmer, model STA6000) by heating the samples to 800 °C in air (25 mL/min) at a rate of 10 °C/min.

## 3 Results and Discussion

### 3.1 Catalytic Activity

Preliminary catalytic results of the DRM reaction are presented in Figure S3. The following increasing trend in catalytic activity (based on CH_4_ and CO_2_ conversions) at identical nickel content was observed: Ni–S < Ni–C < Ni–R. By increasing the amount of nickel from 1 to 4 wt %, the conversion of CH_4_ and CO_2_ increased for all three CeO_2_ morphologies, as did the H_2_/CO ratio. The carbon accumulation rate increases with increasing nickel content (Figure S4A). As a result, further tests were focused on catalysts containing 2 wt % Ni.

**Figure 1:**
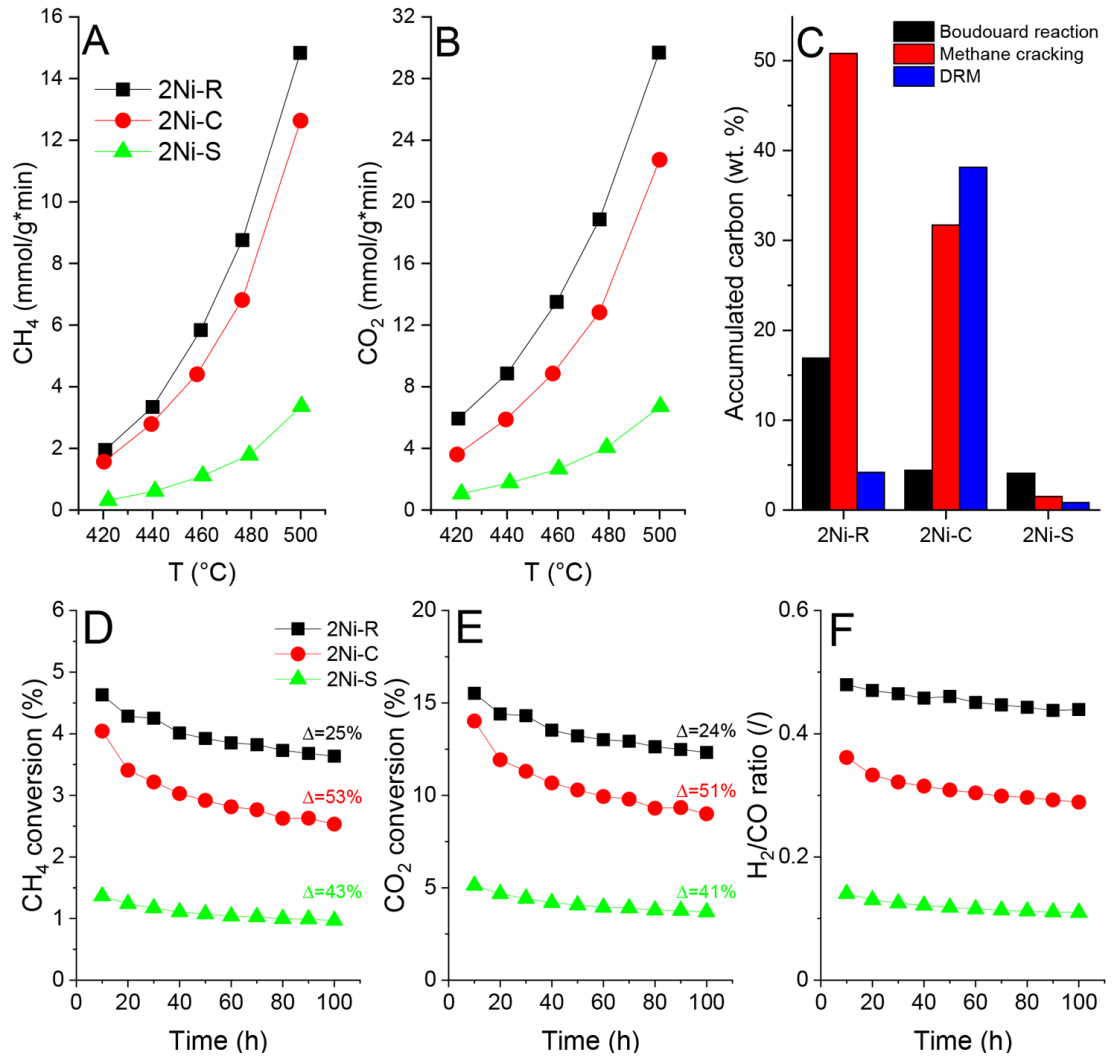
Reaction rates for (A) CH_4_ and (B) CO_2_ as a function of temperature for 2Ni–R, 2Ni–C, and 2Ni–S catalysis. Lines are shown to guide the eye. (C) Carbon accumulated over 2Ni–R, 2Ni–C, and 2Ni–S catalysts during 1 h of TOS in methane cracking and the Boudouard reaction and 6 h of TOS during DRM at 500 °C. Stability test results at 500 °C showing (D) CH_4_ conversion, (E) CO_2_ conversion, and (F) the H_2_/CO ratio versus time on stream.

Over the 2Ni–R catalyst, CH_4_ and CO_2_ rates of 15 and 30 mmol/g_cat_·min were achieved at 500 °C, which were higher than over 2Ni–C (13 and 23 mmol/g_cat_·min) and about 5 times higher than over 2Ni–S (3 and 7 mmol/g_cat_·min) (Figure 1A,B). The reason for the low catalytic activity of 2Ni–S lies in the low accessibility of nickel sites, which was confirmed by pulse CO chemisorption experiments (Figure S5). The CH_4_ and CO_2_ rates over the 2Ni–R catalyst in the temperature range between 420 and 500 °C are more than an order of magnitude higher compared to values in the literature (Table S1 in the Supporting Information). It is evident that we are working with an exceptionally active catalyst.

Stability analysis at 500 °C for 100 h on stream revealed 53, 43 and 25% activity losses occurring over 2Ni–C, followed by 2Ni–S, and the lowest loss over 2Ni–R. In parallel, deactivation results in lowering of the reaction selectivity, namely, the H_2_/CO ratio (Figure 1D–F).

### 3.2 Carbon Deposition

Reactivity and quantity of carbon deposited during DRM, methane cracking (pure CH_4_ feed), and the Boudouard reaction (pure CO feed) were compared (Figure 1C).

The 2Ni–S sample exhibits the strongest resistance toward carbon accumulation (Figure 1C) in all three experiments, which is likely connected to its low intrinsic activity. During the DRM reaction, the amount of deposited carbon was about an order of magnitude higher on 2Ni–C (38 wt %) than on 2Ni–R (4 wt %) and 2Ni–S (2 wt %). However, during methane cracking, the 2Ni–R accumulated the most carbon (52 wt %), compared to 2Ni–C (32 wt %) and 2Ni–S (1.4 wt %) (Figure 1C). This suggests that over the 2Ni–R catalyst, both CH_4_ and CO_2_ activation are fast and kinetically balanced, which ensures efficient carbon gasification during the DRM reaction.^7,8^

The TGA-TPO experiment was used to probe the reactivity of carbon originating from CO, CH_4_, or a mixture of CH_4_ and CO_2_ (DRM) (Figure S4B). Carbon can exhibit markedly different reactivity, which differentiates it from spectator (blocking the catalytic surface and causing deactivation) and reaction intermediates. The latter are able to react with surface hydroxyl or (lattice) oxygen species, thus forming CO.^46−48^ Carbon from CO (eq 4, black trace in Figure S4B) oxidizes at a lower temperature and is more reactive than carbon produced from methane cracking (red trace). Carbon accumulated during DRM (blue trace, Figure S4B) is the most stable, regardless of the CeO_2_ morphology.

### 3.3 Structural Characterization

#### 3.3.1 TEM Analysis

**Figure 2:**
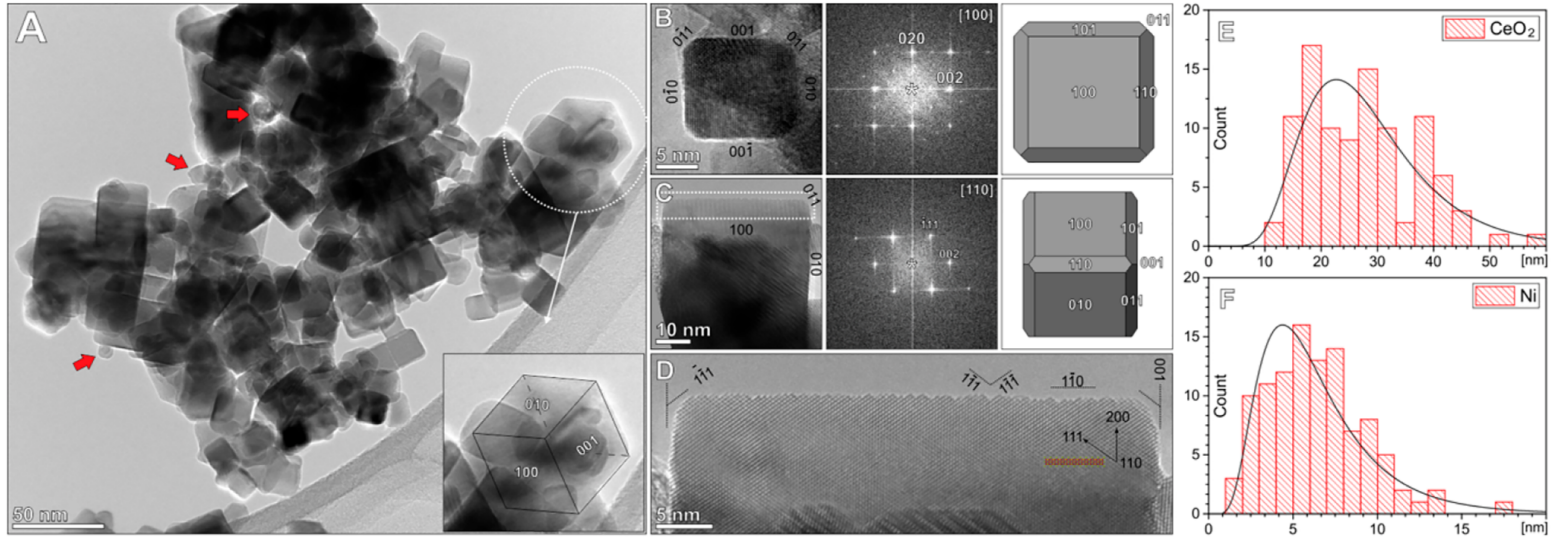
(A) TEM overview of the 2Ni–C catalyst comprising CeO_2_ cubes decorated with Ni nanoparticles (red arrows). (B) Individual CeO_2_ cube aligned in the [100] orientation, with marked Miller indices for exposed crystal faces. The FFT was used for the identification of the zone axis and crystal planes. The schematic of the CeO_2_ cube is in the same orientation, with marked crystal faces at the terraces and truncated edges. (C) CeO_2_ cube in the [110] orientation, with the corresponding FFT and model in the same orientation. (D) HR-TEM of stepped [1–10] edge of the cube, composed of ⟨111⟩ steps, with marked crystal axes, faces, and structure model. Particle size distribution histograms with log-normal distribution curve for (E) CeO_2_ cubes (*N* = 100, σ = 9.7) and (F) Ni nanoparticles (*N* = 105, σ = 2.9).

Visualization of the 2Ni–C catalyst (Figures 2A and S6) revealed CeO_2_ cubes ranging from 10 to 50 nm in size (Figure 2E, mean size 27 nm), which are truncated at the edges and decorated with Ni nanoparticles with a mean size of 6 nm (Figure 2F). The CeO_2_ cubes terminate with {100} crystal facets, with a minor contribution of {110} crystal facets exposed at the truncated part of the cubes (Figure 2B,C). The atomic resolution analysis of the [1–10] facet representing the terrace sites of the truncated part of the cube revealed that it is actually a saw-like surface composed of steps which are about 2–4 atoms thick, following the ⟨111⟩ crystal planes (Figures 2D and S6G).

**Figure 3:**
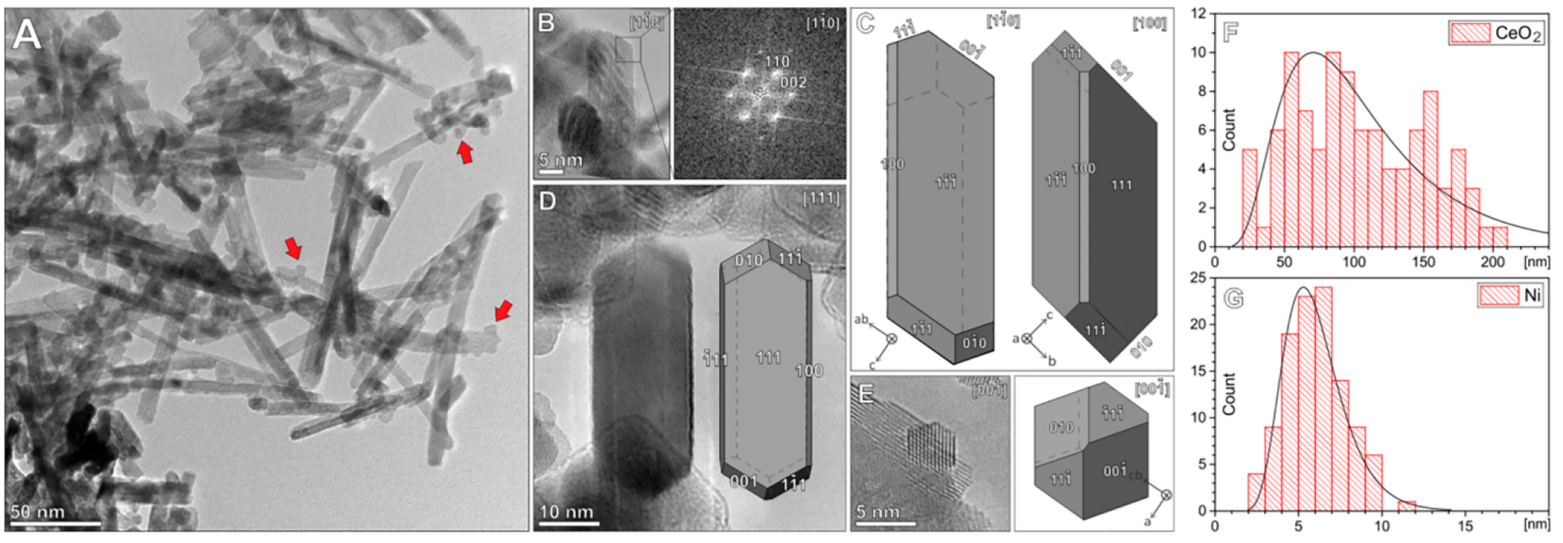
(A) TEM overview micrograph with CeO_2_ rods and Ni NPs (red arrows). (B) Side view, [1–10] with FFT, (C) CeO_2_ rods model with Miller indices for crystal faces, (D) side view, [111], and (E) top view [00-1]. Particle size distribution histogram with log-normal distribution curve for (F) the length of CeO_2_ nanorods (*N* = 102, σ = 46.5) and (G) Ni nanoparticles (*N* = 109, σ = 1.7).

Visualization of the 2Ni–R catalyst (Figure 3 and S7) identified ceria rods measuring about 6–10 nm in diameter and 100–200 nm in length (Figure 3F, mean length 100 nm), decorated with Ni nanoparticles which measure about 6 nm in diameter (Figure 3G, mean size 6 nm). The surface morphology of the ceria rods is mainly composed of {111} facets, with a minor proportion of {100} facets.

The prevailing facet in CeO_2_ rods is {111} and is different from pioneering CeO_2_ nanorod literature^15^ data due to a longer hydrothermal digestion time (24 vs 10 h) and a higher alkali concentration (10 vs 2 M). The extended hydrothermal digestion time apparently retains the overall nanorod shape, but the surface energy minimization favors surface restructuring and termination from {110} to {111}.

**Figure 4:**
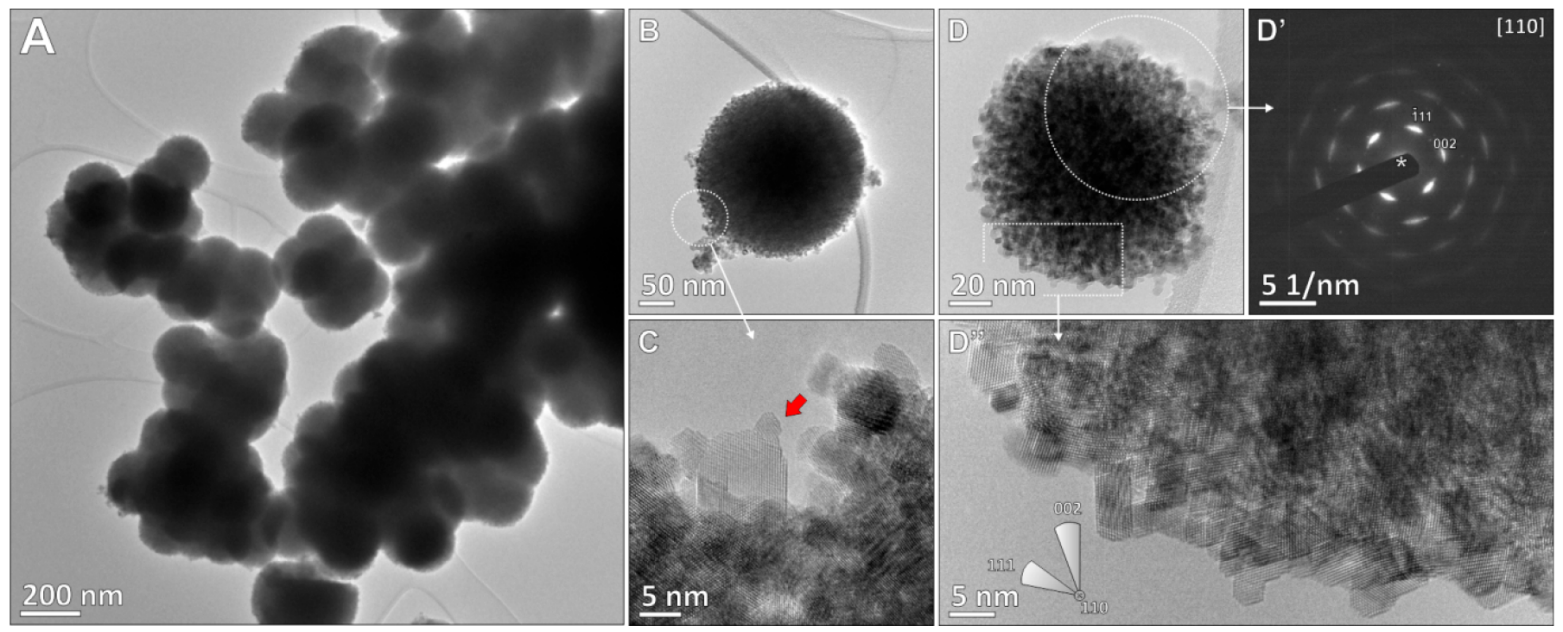
(A) TEM overview micrograph of CeO_2_ sphere agglomerates in the 2Ni–S catalyst and (B) an individual CeO_2_ sphere with (C) nanosized crystalline Ni particles (arrow). (D) Smaller, electron-transparent CeO_2_ sphere with a corresponding (D′) characteristic SAED pattern for textured materials. (D″) HR-TEM micrograph of edge-crystallites with marked main axes. The individual crystallites are misaligned by up to ∼25°.

The CeO_2_ spheres measuring up to 200 nm were visualized in the 2Ni–S catalyst (Figures 4A and S8). The individual CeO_2_ crystallites measuring about 5 nm are closely packed, forming larger spheres (Figure 4B–D). The SAED pattern, recorded over the individual sphere composed of numerous crystallites, does not show the continuous rings characteristic of polycrystalline materials (Figure 4D′). Instead, a single-crystal-like pattern is obtained. When a sphere is tilted in the [110] zone axis, the {110} and {200} diffraction peaks appear to be smothered, a characteristic feature observed in closely packed, self-assembled mesocrystals. Individual CeO_2_ crystallites are mainly terminated by {111} crystal facets.

#### 3.3.2 N_2_ Physisorption and XRD Analysis

The morphological properties are summarized in Table S2 and Figure S9. CeO_2_–S and 2Ni–S exhibit a type I isotherm (characteristic of microporous materials), whereas CeO_2_–R and 2Ni–R as well as CeO_2_–C and 2Ni–C exhibit type II and III isotherms, characteristic of mesoporous materials.

The pore size distribution of CeO_2_–S materials shows a dominant contribution of micropores (*d*_pore_< 2 nm, Figure S9B). For CeO_2_–R and CeO_2_–C materials, the bimodal pore size distribution likely occurs as a result of interparticle porosity among randomly stacked rods and cubes as well as their aggregates. After the deposition of 2 wt % Ni, the BET specific surface area, pore size distribution, and volume change only marginally.

The XRD results before and after nickel deposition are shown in Figure S10A. No differences in crystal structure between different CeO_2_ morphologies were observed, suggesting that all consist of the same fcc crystal structure. After a 2 wt % deposition of nickel, no change in the characteristic CeO_2_ [111] diffraction position is visible (Figure S10B), suggesting that there is no (or that there is below detection limit) NiCeO_*x*_ solid solution formation. Furthermore, no diffraction lines of NiO or metallic nickel were observed (Figure S10C).

#### 3.3.3 Temperature-Programmed Reduction with Hydrogen (H_2_-TPR)

H_2_-TPR was used to analyze the reactivity of oxygen in the materials, which has important consequences for carbon oxidation during the DRM reaction. The reduction of bare CeO_2_ is initiated at about 250 °C, regardless of its morphology, and increases progressively until ∼520 °C (Figure S11). After the sample was held at 550 °C for 30 min, no further H_2_ consumption was noticed, revealing that quasi-steady-state reduction was achieved. The H_2_ consumption, normalized per mass of the sample, is very similar for the CeO_2_–S and CeO_2_–R morphologies, whereas reduction is less intense for CeO_2_–C. This reflects the fraction of reduced Ce^3+^ (quantified on the basis of the amount of H_2_ consumed between 10 and 550 °C: 15 and 17% for CeO_2_–S and CeO_2_–R, respectively), compared to 9% for CeO_2_–C (Table 1). One should keep in mind that H_2_ consumption below 600 °C mainly accounts for the surface reduction of ceria.^49^ Consequently, when normalized per specific surface area, the highest H_2_ consumption is achieved by cubes (7.1 μmol/m^2^) followed by rods and spheres (5.8 and 4.0 μmol/m^2^). Because CeO_2_ cubes have the lowest surface area (Table S2), absolutely speaking they are the least susceptible to reduction, despite being terminated by (100) facets, which exhibit the lowest oxygen vacancy formation energy.^14^

**Table 1:** Fraction of Ce^3+^ Attained during H_2_-TPR^a^, the Amount of CO_2_ Adsorbed, Normalized per Mass or Surface Area of Catalysts, and the Fraction of Adsorbed CO_2_ That Remains Adsorbed at up to 500 °C

sample	fraction of Ce^3+^ (%)	CO_2_ consumption (mmol/g_cat_)	CO_2_ consumption (μmol/m^2^)^b^	CO_2_ remaining adsorbed at 500 °C (%)
CeO_2_–C	9	0.14	4.1 (2.5)	50
CeO_2_–R	17	0.30	3.32 (2)	37
CeO_2_–S	15	0.2	1.93 (1.2)	45
2Ni–C	10	0.14	4.25 (2.6)	68
2Ni–R	18	0.28	3.35 (2)	47
2Ni–S	17	0.24	2.2 (1.3)	47

a See the Supporting Information for calculation details.

b Values in parentheses represent the number of CO_2_ molecules adsorbed per nm^2^ of CeO_2_.

When 2 wt % nickel is deposited over CeO_2_ supports, the reduction starts at ∼60 °C for 2Ni–R and 2Ni–S and at ∼90 °C for 2Ni–C and continues as a cluster of overlapping peaks for up to ∼500 °C, which contains nickel reduction as well as surface and partial bulk ceria reduction.^50,51^ The role of nickel during H_2_-TPR is to facilitate H_2_ dissociation at lower temperatures compared to those for ceria, thus strongly improving the low-temperature oxygen removal from CeO_2_. The highest fraction of Ce^3+^ normalized per mass was achieved on 2Ni–R, followed closely by 2Ni–S, while the lowest was measured for 2Ni–C (Table 1).

#### 3.3.4 CO_2_ Adsorption

The CO_2_-TPD experiments were performed on the *in situ* reduced catalysts to mimic the oxidation state of the catalyst during the DRM reaction. During sample saturation with CO_2_ pulses injected at 25 °C, no gaseous CO was identified, revealing that no reoxidation of the catalyst took place (eq 5).
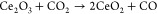
5A clear difference in the CO_2_ adsorption site density (μmol/m^2^) is evident among different ceria morphologies: CeO_2_–C > CeO_2_–R > CeO_2_–S (Table 1).

The CO_2_-TPD profiles (Figure S12) show that all three CeO_2_ morphologies (with or without Ni) possess weak (30–150 °C), intermediate (150–400 °C), and strong (above 500 °C) adsorption sites. At 500 °C, a considerable fraction of CO_2_ remains bound as polydentate carbonates (see below).

After the deposition of nickel, the total amount of adsorbed CO_2_ increases only marginally, whereas the fraction of CO_2_ which remains adsorbed at 500 °C increases. Apparently, nickel notably increases the CO_2_ binding strength. This is likely caused by electron displacement from the nickel clusters to adjacent Ce^3+^ sites (causing increased electron density), which is due to lowering the work function of nickel (5.01 eV) compared to that of partially reduced ceria nanorods (5.34 eV).^52^

During CO_2_-TPD, carbonates can dissociate to CO and O_L_ (lattice oxygen), leading to catalyst reoxidation and release of gaseous CO (eq 5). The identification of a CO signal above 400 °C on CeO_2_–R and CeO_2_–S (Figure S12) shows that these morphologies are able to (partly) reoxidize via the decomposition of adsorbed carbonates. This draws a parallel with a higher reducibility of CeO_2_–R and CeO_2_–S, compared to CeO_2_–C.

#### 3.3.5 Temperature-Programed Activation (TPA) of CO_2_

**Figure 5:**
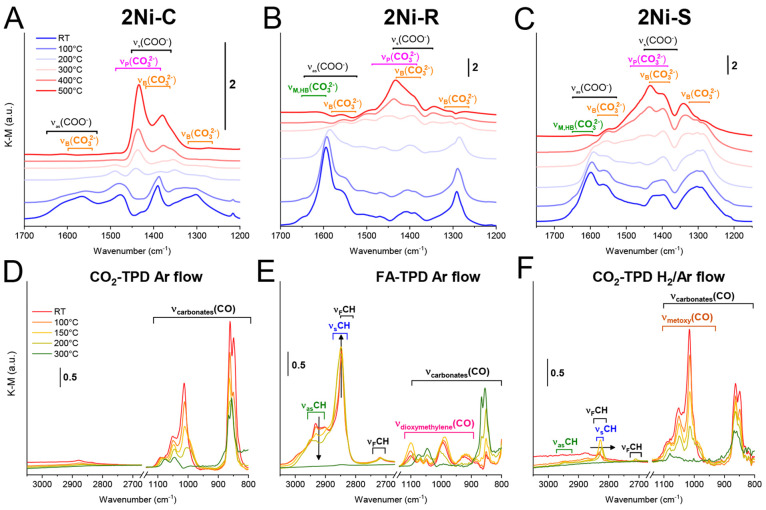
(A–C) DRIFT spectra of reduced 2Ni–C, 2Ni–R, and 2Ni–S catalysts after CO_2_ adsorption at 25 °C and ramping to 500 °C in the 1200–1700 cm^–1^ range. Evolution of νC–O carbonate, methoxy, and dioxymethylene stretching and ν_as,s_C–H formate stretching during (D) CO_2_-TPD and (E) FA-TPD in argon flow and (F) CO_2_-TPD in 5% H_2_/Ar flow over the reduced 2Ni–R catalyst.

**Figure 6:**
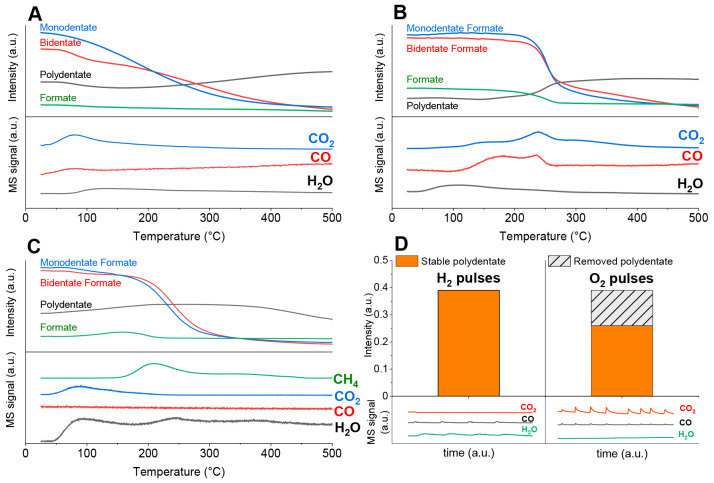
Intensity profiles of characteristic IR bands during (A) CO_2_-TPD, (B) FA-TPD, and (C) CO_2_-TPD in 5% H_2_/Ar flow for a reduced 2Ni–R sample. MS analysis of the evolved gases (bottom panels of A–C) and (D) the polydentate carbonate band intensity (1440–1420 cm^–1^) after H_2_ and O_2_ pulses at 500 °C on a reduced 2Ni–R sample (upper panel) and simultaneous MS analysis of the evolved gases (bottom panel).

The reconstruction and desorption of surface carbonates in the absence of hydrogen on the 2Ni–R, 2Ni–C, and 2Ni–S catalysts were analyzed by temperature-programmed DRIFTS-MS (Figures 5A–C, 6A, and S13 and scenario A; Materials and Methods and the Supporting Information). Prior to the experiments, the samples were reduced *in situ* and flushed with argon at 500 °C for 30 min.

In the region between 1200 and 1700 cm^–1^, bands characteristic of different carbonates on ceria were observed: monodentate (M), bidentate (B), polydentate (P), and hydrogen carbonate (HC).^53^ Between 25 and 200 °C, 2Ni–S and 2Ni–R are populated with bidentate carbonate (1550–1570 cm^–1^). The stretching of monodentate carbonate and hydrogen carbonate (M, HC 1590–1610 cm^–1^)^21,53,54^ occurs in a similar region. On the contrary, on the 2Ni–C catalyst, bands at 1490 and 1385 cm^–1^ are more pronounced compared to 2Ni–S and especially 2Ni–R, identifying a considerable population of polydentate carbonates on ceria nanocubes.

As the samples are heated to 400 and 500 °C, the (COO^–^) stretching intensities of the B and M species decrease, whereas the intensity of bands between 1490 and 1385 cm^–1^ increases. This reveals a reconstruction of M and B into polydentate carbonate (P) species on all three ceria morphologies.

On oxidized bare CeO_2_–R and 2Ni–R catalyst, the carbonate signal is considerably less intense and less diverse compared to that of reduced counterparts (Figure S14), identifying the significance of Ce^3+^ and O_v_ (oxygen vacancy) sites for CO_2_ adsorption and activation and more numerous adsorption modes of CO_2_ on reduced ceria surfaces.

During CO_2_-TPD in argon (Figure 5D), the C–O stretching bands are observed in the region between 800 and 1100 cm^–1^. Because there is negligible C–H stretching between 2800 and 3000 cm^–1^, they belong to different surface carbonates.^21,53,55^

Upon heating to 500 °C, a minor desorption of water is noticed because of sample dehydroxylation (Figure 6A, bottom panel). The monodentate carbonate (1600 cm^–1^, blue trace) and bidentate carbonate (1550–1570 cm^–1^, red trace) intensities decrease. On the other hand, the polydentate carbonate (1420–1440 cm^–1^, dark trace) intensity increases (Figure 6A). This identifies partial decomposition of the carbonates and their desorption as CO_2_ as well as a restructuring of M and B carbonate species into the thermally most stable species, the polydentate. Only a trace change in the C–H band intensity was detected (green trace in Figure 6A).

The CO_2_-TPD DRIFTS-MS experiment over the oxidized 2Ni–R sample (Figure S15A) is characterized by the absence of the monodentate carbonate (1600 cm^–1^) species and a less pronounced polydentate carbonate gain due to lower initial carbonate coverage (Figure S14).

For hydrogen-assisted CO_2_ activation on the reduced 2Ni–R catalyst, two possibilities were analyzed: scenario B, where formic acid was used as a probe molecule because it contains two H atoms per one molecule of CO_2_ (Figure 6B and S16B). The FA vapors were injected over the sample which was held in a flow of argon, followed by sample heating. In scenario C, the temperature-programmed CO_2_ activation was performed while the 2Ni–R sample was held in a flow of H_2_ (Figures 6C and S16C).

Scenario B: Initially at RT, the characteristic stretching C–H (2950–2700 cm^–1^) coexists with the COO– carbonate stretchings, identifying the presence of bidentate and monodentate formate species (BF and MF, Figure S16).

The symmetric C–H stretch between 2878 and 2835 cm^–1^ together with the asymmetric C–H stretch in the 2970–2920 cm^–1^ region is characteristic of the dioxymethylene H_2_COO* group.^56^ Additional proof of the existence of dioxymethylene H_2_COO* species can be obtained from the C–O stretching region: the presence of characteristic bands at 910, 960, and 1100 cm^–1^.^57^ During the FA-TPD DRIFTS experiment, there is an increase in the symmetric C–H stretch (2878–2835 cm^–1^) compared to the asymmetric C–H stretch (2970–2920 cm^–1^). Simultaneously, the C–O stretching of the H_2_COO* increases. This reveals that two formate species are transformed to the dioxymethylene H_2_COO* group as a result of the C–H bond cleavage and the transfer of H to the carbon atom with the simultaneous release of CO gas between 100 and 200 °C (Figure 6B).

Changes in the νC–H stretching and C–O stretching above 200 °C are caused by restructuring of the BF, MF, and H_2_COO* into P and the desorption of CO, CO_2_, and H_2_O (the latter was found only in the case of an oxidized 2Ni–R sample, Figure S15B). At 300 °C, all formate and H_2_COO* are decomposed and C–H bands disappear. Because formates are less thermally stable than carbonates, they decompose at lower temperatures and the population of inert polydentate carbonates stabilizes at about 300 °C in scenario B, compared to at 500 °C in scenario A.

During temperature-programmed CO_2_ activation in a flow of H_2_ (scenario C; Figures 5F, 6C, and S16), the intensity of C–H bands starts to increase at about 100 °C, reaches a maximum at 150 °C, and completely decays at 250 °C (green trace in Figure 6C). This is about 50 °C earlier compared to the case of scenario B (Figure 6B). This signifies the effect of excess hydrogen, which assists CO_2_ conversion into formate species.^58,59^ Unlike C–H stretching during FA-TPD, the C–H stretching observed in scenario C is shifted to lower wavenumbers (2830 cm^–1^, Figure 5F). The combination of the symmetric C–H stretching at 2830 ± 10 cm^–1^ with the CO stretching between 1100 and 1000 cm^–1^ is characteristic of the O–CH_3_ methoxy group.^56,60,61^ The carbonate species also give rise to C–O stretching in the 1100–800 cm^–1^ region, but with a higher intensity at 850 cm^–1^ (Figure 5D). When methoxy species are present in notable amounts, the intensity of bands at 850 are lower compared to that from 1100 to 1000 cm^–1^ (Figure 5F)

In contrast to scenarios A and B (Figure 6A,B), the gain in P carbonates is much smaller and actually decreases to below the initial values. In excess H_2_, the decomposition pathway of MF and BF is shifted away from P and steered toward methane and water (green trace, bottom panel of Figure 6C), apparently through the formate and methoxy intermediates (together with the disappearance of C–H and C–O stretching, Figure 5F).^62^ Under hydrogen-lean conditions, formate and H_2_COO* decompose to CO.

Pulse CO_2_ isothermal experiments at 500 °C (Supporting Information, Figure S17) in the presence of hydrogen increase CO formation and correlate well with the temperature-programmed experiments described above (scenarios A–C).

#### 3.3.6 Probing Carbonate Reactivity with H_2_ and O_2_ Pulse Experiments

Reduced 2Ni–R was pretreated with CO_2_ at 500 °C (Figure S1B) to populate its surface with polydentate carbonates, H_2_ pulses were injected at the same temperature, and DRIFTS-MS was used to analyze the catalyst surface and evolved gases. During the H_2_ pulses, no decrease in the polydentate signal was observed (Figure 6D). Consequently, in the transient experiment, H_2_ is inefficient in reacting with the polydentate carbonate at a kinetically relevant rate. Therefore, it is reasonable to assume that they mainly act as spectators during the DRM reaction. The MS signal for water (*m*/*z* = 18) and the trace signal for CO were observed during H_2_ pulsing (bottom panel of Figure 6D). This suggests that H_2_ preferably reacts with lattice oxygen, causing catalyst reduction as well as removing the remaining M and B carbonates.

Contrary to hydrogen, oxygen pulses at 500 °C caused a noticeable drop in the polydentate band intensity (Figure 6D). Catalyst oxidation caused the destabilization and desorption of surface carbonates mainly as CO_2_ and trace amounts of CO (bottom panel in Figure 6D). This is consistent with (a) a much lower density of surface carbonates on oxidized compared to partially reduced ceria surfaces (Figure S14) and (b) DFT calculations reported in section 3.3.7.

#### 3.3.7 *In Situ* Ni K-Edge XANES and EXAFS Analysis

At RT in air, nickel is present exclusively as Ni^2+^ on all three CeO_2_ morphologies (Figures 7 and S18–S25). After activation in a 5% H_2_/N_2_ flow, between 60 and 70% of the Ni^2+^ is reduced to metallic Ni (Ni^0^). This fraction of Ni^0^ is retained during the DRM reaction at 400 °C.

**Figure 7:**
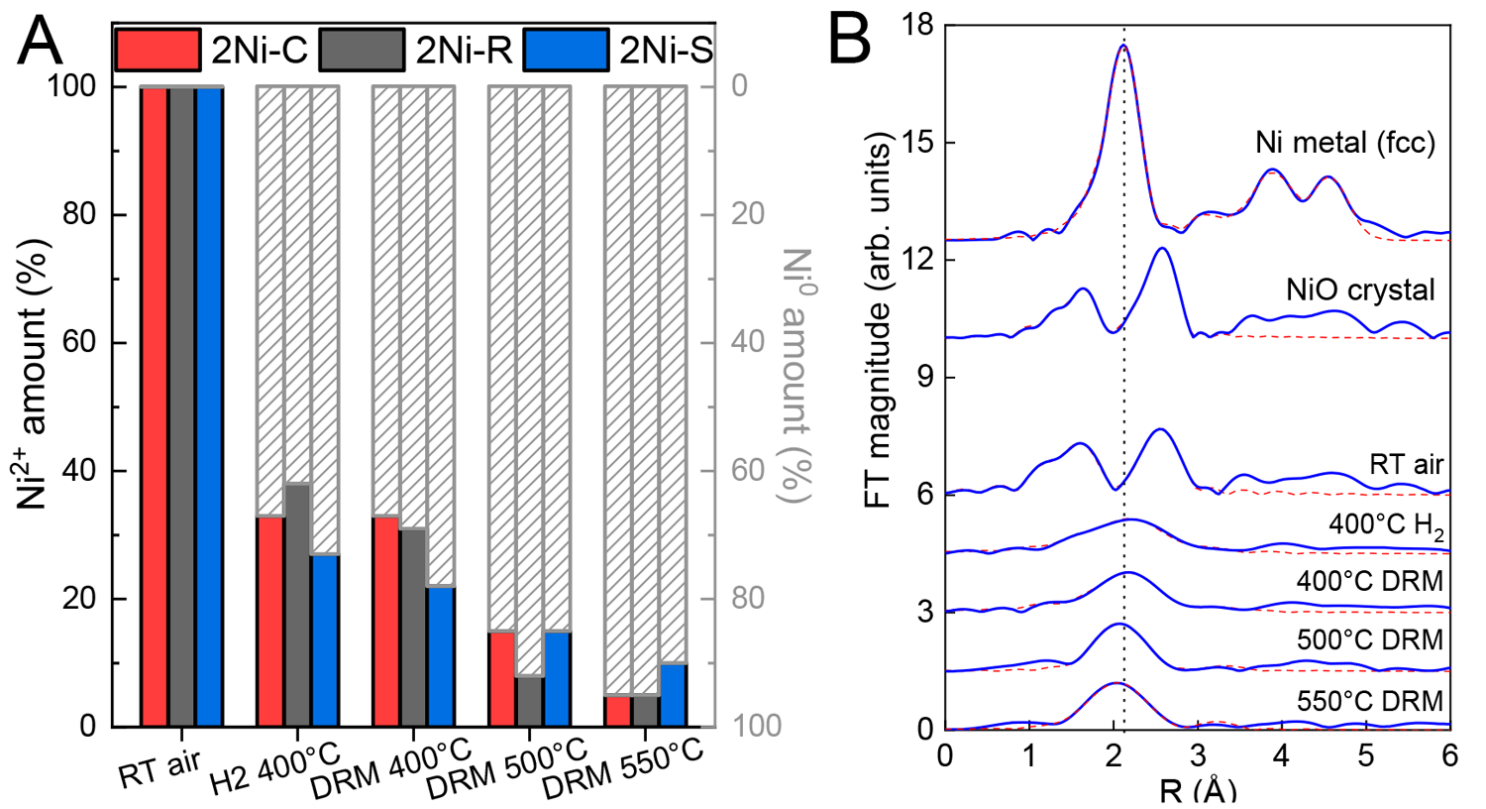
(A) Relative amounts of Ni^2+^ and Ni^0^ in 2Ni–C, 2Ni–R, and 2Ni–S catalysts and (B) Fourier transform magnitude of *k*^2^-weighted Ni K-edge EXAFS spectra of the 2Ni–R catalyst measured in situ at RT in air, after reduction in 5% H_2_/N_2_ flow for 1 h at 400 °C and during DRM reaction in equimolar CO_2_/CH_4_ flow at 400, 500, and 550 °C, calculated in the *k* range of 3–10 Å^–1^. Experiment, solid line; best fit EXAFS model calculated in the *R* range of 1 to 3.5 Å, dashed line. The spectra of two reference Ni compounds (NiO and Ni metal (fcc)) are added for comparison. Spectra are shifted vertically for clarity. A vertical dotted line is plotted at the first peak position in the FT spectrum of Ni metal.

During the DRM reaction at 500 °C, the fraction of Ni^0^ increased to 85, 85, and 90% in 2Ni–C, 2Ni–S, and 2Ni–R, respectively. At 550 °C, the fraction of Ni^0^ exceeds 90% in all catalysts. A very similar trend in the temperature-dependent evolution of the nickel oxidation state was also observed for the 4Ni–R catalyst (Figure S22).

*In situ* Ni K-edge EXAFS analysis was used to monitor the changes in the local structure around nickel under the above-mentioned conditions. In all studied catalysts in air at RT, Ni^2+^ is coordinated with oxygen atoms in the first coordination shell and with Ni and O in more distant coordination shells at distances characteristic of NiO (Figures S26–S29). In the 2Ni–R sample, the coordination numbers are significantly lower than in the bulk NiO (Tables S3–S5), indicating that nickel is in the form of small clusters, measuring below 1 nm in diameter.^63^ The estimated size of NiO clusters does not change when the nickel content is increased from 2 to 4 wt %: their structural parameters are the same (Table S5). In the 2Ni–C catalyst, the size of NiO nanoparticles is estimated to be about 2 nm.

In the case of 2Ni–S, the NiO nanoparticle size, estimated from the average coordination number, is smaller compared to those in the 2Ni–R sample. All Ni^2+^ cations are connected to Ce atoms (forming Ni–O–Ce bridges), indicating that nickel is in the form of two-dimensional thin islands, coating the CeO_2_ surface.

EXAFS is a bulk analysis method and is not as localized as TEM visualization. The variation in the NiO particle size, estimated by TEM and EXAFS, likely originated from the polydisperse size distribution of NiO in the catalysts. The NiO crystallites measuring about 6–10 nm (visualized by TEM) coexist with the subnanometer NiO clusters, which are the majority phase (detected by EXAFS).

During catalyst activation at 400 °C, the nearest-neighbor Ni–Ni distances are the same as in Ni metal fcc (2.48 and 3.52 Å). More distant Ni neighbors expected in the Ni fcc metal structure are below the limit of detection, indicating that the average size of Ni metal clusters is below 1 nm. A portion of Ni cations remain in the Ni^2+^ state, as demonstrated by the XANES analysis, and the local structure remains the same as in the NiO crystal (Ni–O and Ni–Ni distances are the same as in the NiO crystal: 2.07 and 2.97 Å, respectively). During the DRM reaction at 400 °C, there are no significant structural changes in the Ni metal or NiO clusters (Tables S6–S9).

During the DRM reaction at 500 and 550 °C, some changes in the local Ni structure are observed among the three CeO_2_ morphologies. In the case of 2Ni–R, the Ni–Ni coordination number of the metallic species becomes higher than that in 2Ni–C. The lowest Ni–Ni coordination remains in 2Ni–S. This reveals that under the DRM conditions at 500 and 550 °C the restructuring of the nickel phase is most prominent on ceria nanorods. Mao and Campbell^64^ report that an oxophilic metal such as nickel is repelled by surface oxygen vacancy sites of ceria and that nickel prefers to be associated with surface oxygen atoms. The most extensive reduction of the 2Ni–R catalyst (Table 1) results in fewer surface oxygen sites, where nickel is preferably aggregated, favoring nickel coalescence and the formation of larger Ni clusters. Over ceria nanocubes, the fraction of Ce^3+^ is lower, resulting in a higher abundance of oxygen sites which bind nickel more strongly. Also, the jagged surface of ceria nanocubes (Figure 2D) makes the diffusion of nickel across the step sites energetically much more demanding compared to the diffusion over extended closely packed (111) terraces. Furthermore, if single atom nickel atoms were present in substantial amounts, then the Ni–O–Ce chemical bonds would be discovered during EXAFS fitting. This was not the case.

#### 3.3.8 DFT Calculations

On the basis of TEM characterization, {111} and {100} are the most abundant terminating facets on the synthesized CeO_2_ materials. We include the {110} facets in our calculations, which are also commonly exposed.^12,13,65,66^

##### 3.3.8.1 CeO_2_(111)

For {111}, the stoichiometric surface will be stable under high oxygen pressures.^67^ Under lower pressures, the surface gets progressively reduced.^68^ It has been shown that vacancies form in the first subsurface layer and do not agglomerate.^69^ In the oxygen-lean limit, all subsurface oxygen atoms are removed and the ensuing structure undergoes a reconstruction with a change in the stacking of the surface Ce and O layers, gaining 0.3 eV.^70^

We investigate the stoichiometric surface (CeO_2_(111)), the surface with 0.25 ML of subsurface vacancies (CeO_2_(111)_(1/4ss)_), and the surface with 1.00 ML of subsurface vacancies and the stacking fault (CeO_2_(111)_(1ss+stacking)_).

When oxygen is removed from the surface, a surface vacancy is first created (CeO_2_(111)_(1/4s)_). Overcoming a small barrier of 0.27 eV, the subsurface oxygen migrates to the surface, forming CeO_2_(111)_(1/4ss)_, which is 0.28 eV more stable. However, the vacancies in the subsurface are immobile (*E*_a_ = 2.81 eV for diffusion), and the less stable surface vacancies can diffuse at high temperature (*E*_a_ = 1.94 eV for diffusion). This explains why vacancies do not agglomerate.^69^

A fully reduced surface CeO_2_(111)_(1ss)_ restructures to CeO_2_(111)_(1ss+stacking)_ upon overcoming a barrier of 1.05 eV, lowering its energy for 0.38 eV. This is in agreement with the findings from Lustemberg et al.^70^ All structures are shown in Figure S29.

##### 3.3.8.2 CeO_2_(110)

On stoichiometric CeO_2_(110), oxygen vacancies can also form on the surface and in the subsurface.^71^ A surface oxygen vacancy is thermodynamically 0.85 eV more stable than a subsurface one. Subsurface vacancies are also kinetically unstable, requiring only 0.04 eV to migrate to the surface. Diffusion of the oxygen vacancies on the surface can happen along (*E*_a_ = 1.76 eV) or across (*E*_a_ = 0.92 eV) the Ce–Ce rows. Thus, we will limit our calculations to the stoichiometric surface and surface vacancies. All structures are shown in Figure S29.

##### 3.3.8.3 CeO_2_(100)

Because of the polarity of the CeO_2_(100) surface, half of the surface oxygen atoms must be displaced to the bottom of the slab (Model B0 from ref (43)). Adding an additional formula unit of CeO_2_ to the surface yields Model C0, which is slightly more stable. We will analyze both terminations because of their almost identical surface energies (γ_C0_ = 0.102 eV Å^–2^, γ_B0_ = 0.105 eV Å^–2^).^43^ For the B0 surface, we investigate the structure with one surface oxygen vacancy (B1_s_) and with one subsurface oxygen vacancy (B1_ss_). For the C0 surface, two surface oxygen vacancies (C2) are considered.

On B0, a surface oxygen vacany is slightly (0.14 eV) more stable than a subsurface one. The barrier for its migration in the subsurface is also low (*E*_a_ = 0.23 eV). Hence, both structures can exist under oxygen-lean experimental conditions. In the subsurface, there are two unequivalent sites for oxygen vacancies: below the surface oxygen atoms (structure B1_ss_) and below the Ce–Ce bridge position, with the latter being much less stable (+1.33 eV). Consequently, diffusion in the subsurface is not likely (*E*_a_ = 1.37 eV), whereas it proceeds readily on the surface (*E*_a_ = 0.63 eV). Figure S30 shows all five mentioned surface structures of ceria.

##### 3.3.8.4 CO_2_ Adsorption on CeO_2_(111) Surfaces

CO_2_-TPD clearly shows three types of adsorbed CO_2_ on the partially reduced CeO_2–*x*_ surfaces (Figures 5B and S12), which are assigned to the monodentate (M), bidentate (B), and polydentate (P) species. This is in excellent agreement with the DFT data for the (111) surface at all levels of oxidation (CeO_2_(111), CeO_2_(111))_(1/4ss)_, and CeO_2_(111))_(1ss+stacking)_).

When CO_2_ is planarly positioned above the surface, the geometric and electronic structures are not perturbed noticeably and a very weak interaction of −0.15 eV (irrespective of the surface) results, which is merely physisorption.

CO_2_ can bind in a monodentate fashion in the tilted orientation atop the Ce atom, forming an angle of 54° with the surface at a O–Ce distance of 2.96 Å. The interaction energy is −0.24 eV on all surfaces. There is negligible charge transfer from the catalyst to CO_2_, no geometric distortion of CO_2_, and no perturbation in the density of states (Figure S31).

CO_2_ can also adsorb in a bidentate fashion through Ce–O and O_surf_–C with physisorption (−0.38, −0.28, and −0.33 eV on CeO_2_(111), CeO_2_(111)_(1/4ss)_, and CeO_2_(111)_(1ss+stacking)_), thus effectively forming a carbonate species with one free (dangling) oxygen. In CO_2_, the O–C–O angle is distorted from 180 to 131° and the O–C bonds are elongated to 1.29 and 1.22 Å. The Ce–O bond length is 2.38 Å, and the O_surf_–C bond length is 1.43 Å. There is some charge transfer as the CeO_2_(111) surface donates 0.15*e*^–^ to CO_2_, the CeO2(111)_(1/4ss)_ surface donates 0.21*e*^–^, and the CeO_2_(111)_(1ss+stacking)_ surface donates 0.27*e*^–^.

**Figure 8:**
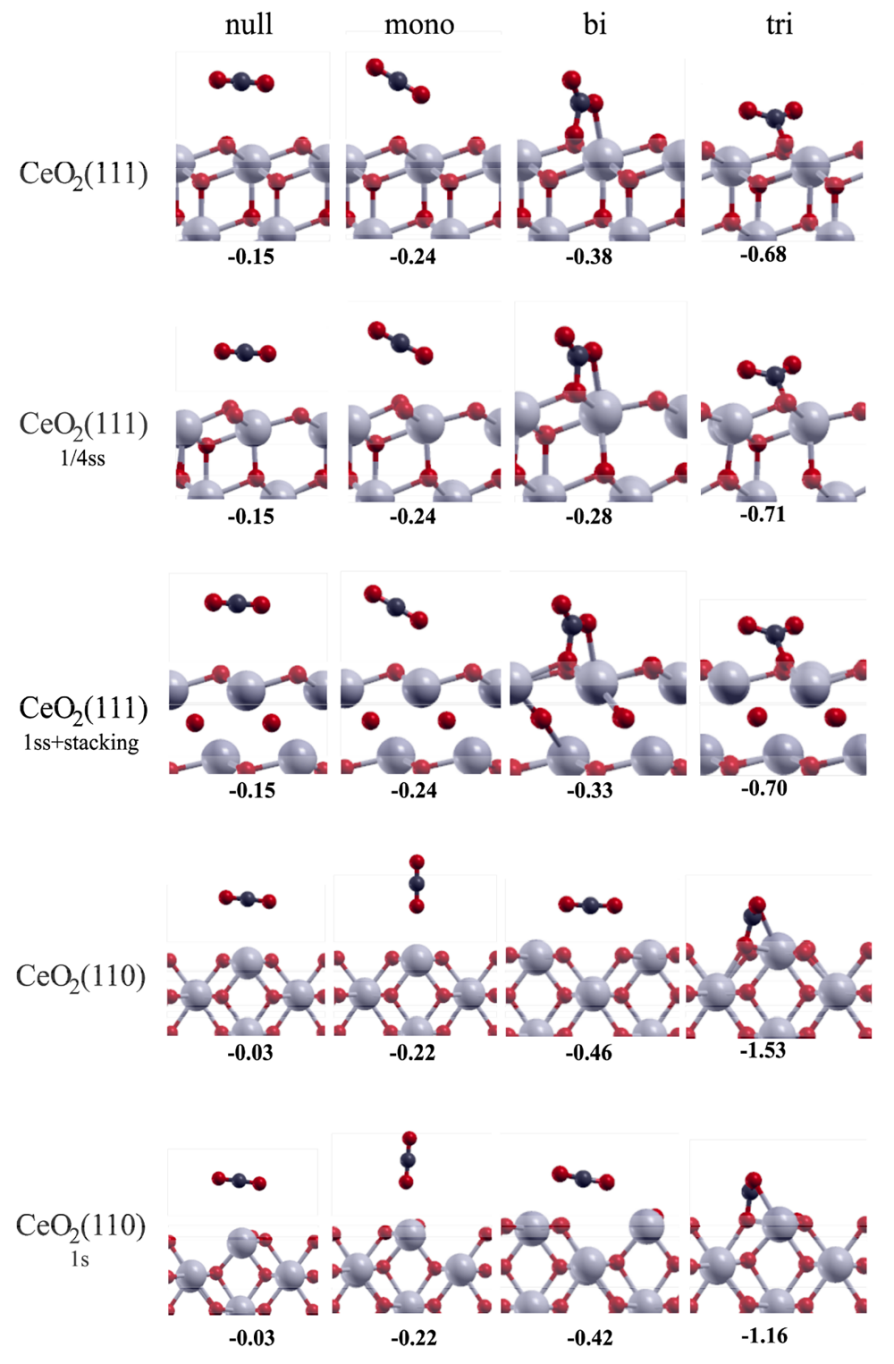
Adsorption modes of CO_2_ on CeO_2_(111), CeO_2_(111)_(1/4ss)_, CeO_2_(111)_(1ss+stacking)_, CeO_2_(110), and CeO_2_(110)_(1s)_. The values represent adsorption energies in eV.

The strongest interaction of CO_2_ with CeO_2_(111) occurs when it adsorbs as a polydentate/tridentate species (−0.68, −0.71, and −0.70 eV on CeO_2_(111), CeO_2_(111)_(1/4ss)_, and CeO_2_(111)_(1ss+stacking)_, respectively, forming one Ce–O and two O_surf_–C interactions. The resulting carbonate species has the original C–O bonds elongated to 1.27 Å and the O–C–O angle is further decreased to 129°. The Ce–O bond lengths are 2.54 Å and the O_surf_–C bond length is 1.38 Å. The charge transfer is 0.17 *e*^–^ from CeO_2_(111) and 0.27 *e*^–^ from CeO_2_(111)_(1ss+stacking)_. As shown in Figure S31, the electronic density of the surface is not noticeably perturbed by the adsorbed CO_2_ molecule. The three adsorption modes are shown in Figure 8. The adsorption interaction and charge transfer slightly increase as the surface gets reduced.

We estimate the approximate temperatures, where differently bound CO_2_ is released, setting Δ*G*(*T*)_ads_ = 0. The monodentate species would desorb from CeO_2_(111) at 99 °C, the bidenatate species at around 316 °C, and the polydentate/tridentate/carbonate species at 781 °C. On the reduced surfaces, these values are comparable. Note that the values are strongly dependent on the accuracy of DFT data and can easily vary for ±100 K because of the uncertainty of the DFT data. Nevertheless, they agree qualitatively with Figure S12, which shows three distinct peaks during CO_2_-TPD.

##### 3.3.8.5 CO_2_ Adsorption on CeO_2_(110) Surfaces

On the stoichiometric CeO_2_(110), three proper adsorption modes are possible, which is again in agreement with the CO_2_-TPD (Figures 5 and 6A). If CO_2_ is positioned horizontally 3.4 Å above a Ce atom, then the interaction is negligible (−0.03 eV). However, CO_2_ can bind as a monodentate species in a vertical position at a O–Ce distance of 2.84 Å (−0.22 eV) without a geometry distortion. When bound as a bidentate species (−0.46 eV), two O–Ce bonds are formed (2.9 Å). The strongest interaction (−1.53 eV) occurs when CO_2_ forms a tridentate (carbonate) species with a surface oxygen atom. The O–C–O angle approaches the ideal carbonate angle, and the O–C bonds are elongated (1.28 Å). There is a considerable charge transfer of (−0.21*e*_0_) from the surface to the CO_2_. The entire carbonate species is negatively charged (−1.39*e*_0_). On the surface with a surface vacancy (CeO_2_(110)_(1s)_), the adsorption modes are analogous but the interaction is slightly weaker. The monodentate species binds with 0.22 eV, the bidentate species with 0.42 eV, and the carbonate species with 1.16 eV.

##### 3.3.8.6 CO_2_ Adsorption on CeO_2_(100) Surfaces

On different CeO_2_(100) structures, the situation is more complex. First, we treat B0 and B1_ss_ structures concomitantly because they differ only in one subsurface oxygen and feature the same adsorption modes. When CO_2_ is planarly above the surface Ce atom, the purely physisorption interaction is weak (−0.07 eV). When CO_2_ binds as a monodentate species, it sits in the bridge position of the removed surface oxygen atom, forming an angle of 37.4° with the surface at a O–Ce distance of 2.76 Å. The geometry of the molecule is not distorted, and there is no charge transfer. The adsorption interaction is −0.49 eV.

Somewhat surprisingly, the bidentate mode of adsorption is weaker (−0.38 and −0.09 eV for B0 and B1_ss_, respectively). Here, upon the formation of the Ce–O and O_surf_–C bonds, CO_2_ is adsorbed as a carbonate species with one free (dangling) oxygen. The O–C–O angle is reduced to 131 °C, and the O–C bonds are elongated to 1.31 Å (toward C(−O_surf_)) and 1.21 Å (toward the dangling oxygen). CO_2_ interacts most strongly with CeO_2_(100) when it binds as a tridentate species with one Ce–O and two O_surf_–C interactions. Incorporating into the lattice horziontally, the adsorption energies are −2.45 and −1.66 eV on B0 and B1_ss_, respectively. A proper carbonate species with three equally long C–O bonds (1.30 Å) is formed. There is a considerable charge transfer from the surface to the CO_2_ molecule (−0.27*e*_0_), and the entire carbonate molecule is negatively charged (−1.33*e*_0_). Experimentally, two distinct adsorption modes can be observed: the monodentate and the carbonate species. However, under oxygen-lean conditions there will be surface oxygen vacancies present, which introduces additional possibilities, as shown below.

On the B1_s_ surfaces, more adsorption modes are accessible because of the available oxygen vacancy. Again, CO_2_ can interact planarly (−0.07 eV), as a monodentate species (−0.60 eV), as a bidentate species (−0.91 eV), or as a carbonate species (−3.28 eV). The two additional adsorption modes of CO_2_ include a bent geometry: the carbon atom can occupy the hollow (−0.57 eV) or the bridge surface site (−0.87 eV), with the CO_2_ molecule positioned horizontally.

**Figure 9:**
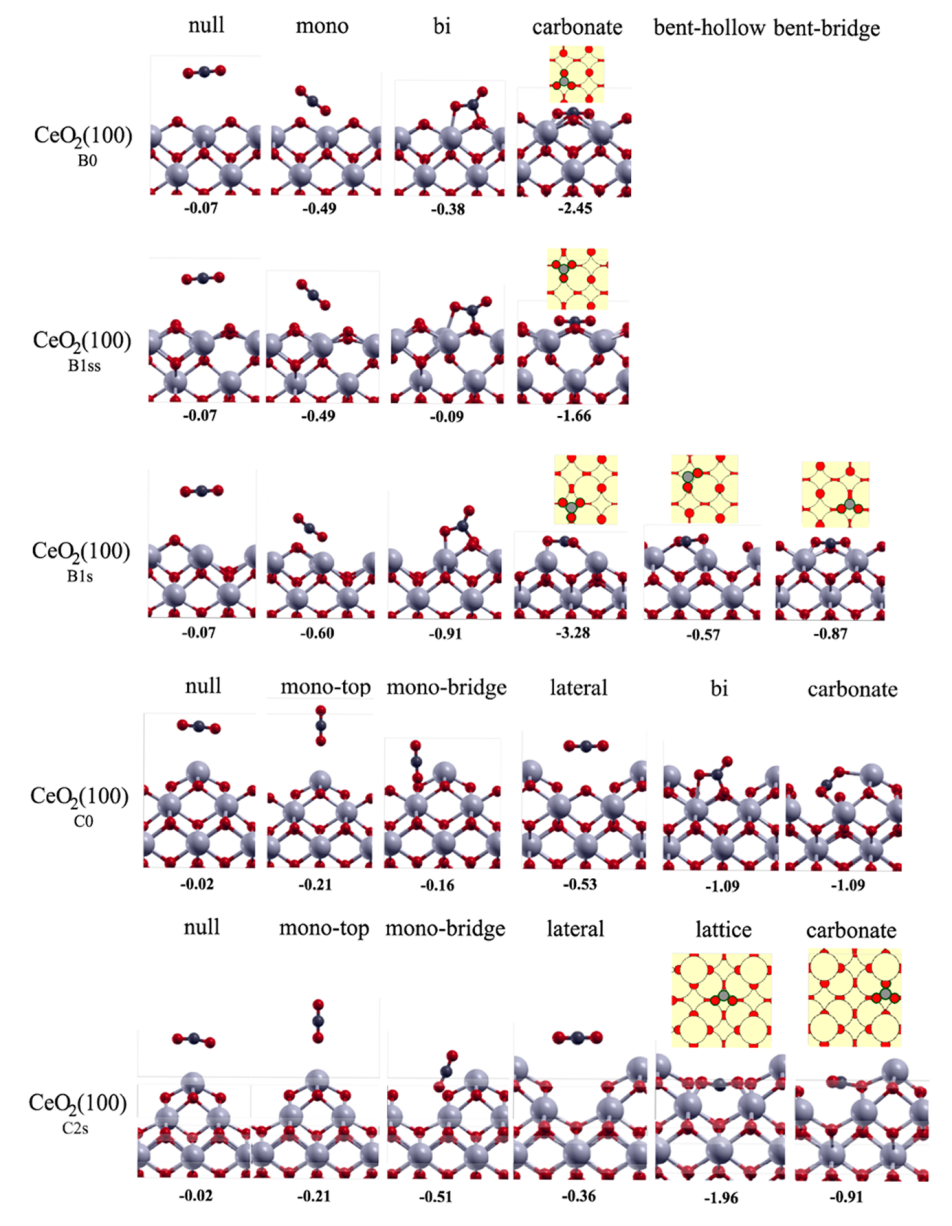
Adsorption modes of CO_2_ on CeO_2_(100):B0, CeO_2_(100):B1ss, CeO_2_(100):B1s, CeO_2_(100):B2s, CeO_2_(100):C0, and CeO_2_(100):C2s. Adsorption values are in eV.

C0 and C2 exhibit similar adsorption modes of CO_2_. When located horizontally above the surface Ce atom, the CO_2_ interaction is negligible (−0.02 eV). It can also adsorb as a monodentate species atop the Ce (−0.21 eV) without noticeable geometry distortion, electronic perturbation, or charge transfer. On C2, a bridge position for the monodentate species is also possible (−0.51 eV), while on C0 the bridge position is not particularly stable (−0.16 eV). CO_2_ can be positioned between the adjacent Ce adatoms (−0.53 and −0.36 eV on C0 and C2, respectively). On C0, there are two distinct, equally strong adsorption modes. CO_2_ can bind as a bidentate (Ce–O and O_surf_–C) or a carbonate (Ce–O and two O_surf_–C) species, with both exhibiting an adsorption energy of −1.09 eV. On C2, the carbonate species is bound similarly strongly (−0.91 eV). However, because of two missing surface oxygen atoms, there is a particularly strong (−1.96 eV) adsorption mode with CO_2_ incorporating into the lattice on the surface. All adsorption modes are shown in Figure 9.

##### 3.3.8.7 CO_2_ Adsorption on the Ni(111) Surface

The adsorption of CO_2_ on nickel has already been thoroughly researched by Wang et al.^72^ Using the GGA level of DFT, adsorption energies on Ni(111), Ni(110), and Ni(100) were calculated. The authors found that on Ni(111), which is the most stable and common nickel surface, CO_2_ can adsorb in four different ways with similar interactions (0.31–0.46 eV). Our results confirmed these and found that the adsorption energy of the lowest-lying intermediate is 0.53 eV, which is in reasonable agreement with the data from Wang et al. In the most favorable configuration (4-5fcc), CO_2_ assumes a bent geometry with a O–C–O angle of 132°. The carbon atom is positioned between the fcc and bridge sites, and the oxygen atoms interact with two neighboring Ni atoms (*d*(Ni–O) = 2.11 Å). The other adsorption modes are similar in energy (0.3–0.5 eV). See Figure S32 and Table S10 for geometries and energies.

Although we studied the adsorption of CO_2_ and the formation of different surface motifs, the decomposition of CO_2_ into CO has been studied elsewhere. Because of a strong repulsion between the surface oxygen atoms and the newly formed O*, this is feasible on oxygen-deficient surfaces. Kildgaard et al. studied this reaction on CeO_2–*x*_(111) and found that carbonate cannot decompose to CO because of a prohibitively high activation barrier, whereas the less stable bidentate CO_2_^–^ could decompose with a barrier of 0.57 eV.^73^ Similarly, CeO_2_(110) is conducive for this reaction only when oxygen vacancies are represent. Kumari et al. have shown that CeO_2_(110) with a single oxygen vacancy cannot decompose CO_2_ to CO because of a barrier of 2.6 eV. Hence, a secondary metal (Ni) is required.^74^

## 4 Discussion

Chemically identical catalysts, all containing 2 wt % nickel on ceria rods, cubes, and spheres exposing predominantly (111) and (100) facets, provide very different activity, stability, and carbon accumulation resistance in the low-temperature DRM reaction. We established the following decreasing trend in catalytic activity: 2Ni–R > 2Ni–C > 2Ni–S. The initial size of the nickel clusters, estimated via *in situ* EXAFS analysis, was found to be very similar on all ceria shapes (∼1 to 2 nm), as was the fraction of metallic nickel which reaches 90–95% at 550 °C during the DRM reaction.

A strong inverse correlation exists between the carbon accumulated during the DRM reaction and the fraction of Ce^3+^ quantified during H_2_-TPR analysis. The facile reduction of the 2Ni–R catalyst terminated predominantly with the (111) facet enables efficient carbon gasification during DRM with mobile oxygen species originating from CO_2_.^7,51^

The lowest catalytic activity and carbon deposition on 2Ni–S are due to the partial inaccessibility of Ni active sites to gaseous reactants, caused by the close packing of ceria crystallites.

The catalyst deactivation strongly depending on the surface morphology of nanoshaped ceria is most extensive over 2Ni–C terminated with the (100) facet. During methane cracking in the absence of oxidant, the highly active 2Ni–R sample was most extensively coked. However, the 2Ni–C accumulated by about an order of magnitude more carbon during DRM compared to two other tested catalysts.

The following phenomena likely contribute to extensive coking and deactivation over 2Ni–C: (i) the redox chemistry of ceria cubes is least prominent, leading to lagging oxygen supply to the nickel, resulting in slow carbon removal, and (ii) the surface of cubes during the DRM reaction is most densely populated with stable polydentate carbonate spectator species, which additionally hinders oxygen mobility and the participation of oxygen species in the catalytic cycle.

Our DFT calculations reveal a much stronger adsorption interaction of the carbonate species on stoichiometric CeO_2_(100) (2.45 eV) than on CeO_2_(111) (0.7 eV) or CeO_2_(110) (1.53 eV).

Moreover, both DFT and CO_2_-TPD analyses show that oxygen-deficient {100} facets provide stronger binding sites compared to {111}.

According to experimental data shown in Table 1, 2.0–2.6 CO_2_ molecules bind per nm^2^ of CeO_2_ (rod and cube morphology, respectively) and 1.2–1.3 bind per nm^2^ in CeO_2_ spheres, which is due to the close stacking of individual ceria crystals and the poor accessibility of its surface.

The interaction between Ni(111) and CO_2_ has been computed to be roughly 0.5 eV, whereas on various CeO_2–*x*_ surfaces this interaction can exceed 1.0 eV for polydentate species.

Consequently, CO_2_ predominantly adsorbs and undergoes activation on CeO_2–*x*_ and not nickel. This is further confirmed by the similarity of DRIFT spectra of pure ceria supports and Ni–CeO_2_ catalysts as well as the difference spectra at 500 °C (Figures S13 and S33).

For the sake of completeness, we also analyzed a 2 wt % Ni/SiO_2_ sample under identical conditions as for Ni/CeO_2_–R for the carbonate population (Figure S34). We can see a much lower (negligible) carbonate signal on Ni/SiO_2_. This indicates a fundamentally different chemistry of both materials (Ni/SiO_2_ and Ni/CeO_2_) for CO_2_ activation and adsorption.^75^

The function of nickel is methane activation and the supply of hydrogen species via spillover to facilitate the reaction of different surface carbonates (excluding polydentate).

The following arguments regarding the reactivity of different carbonates, populating fully oxidized or partially reduced (111) ceria nanorod surfaces, can be put forward.

**Figure 10:**
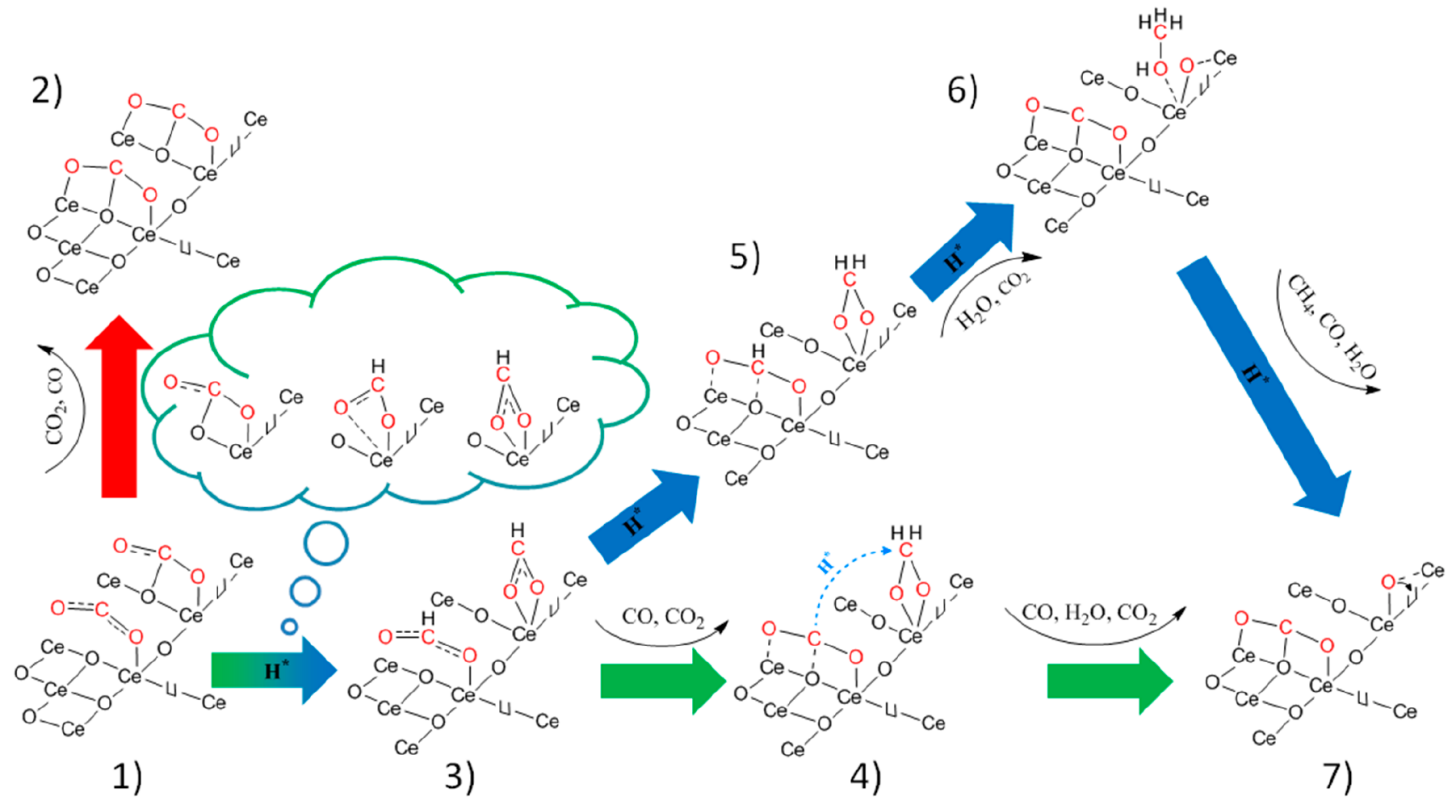
Proposed mechanism of CO_2_ activation with and without hydrogen assistance on a partially reduced (111) ceria surface. Red, green, and blue arrows represent CO_2_ reaction pathways under a hydrogen-free, equimolar H_2_/CO_2_ ratio and surplus H_2_ conditions, respectively.

On the basis of the temperature-programmed DRIFTS-MS experiments, a mechanism of CO_2_ activation on a partially reduced ceria surface in the absence or presence of hydrogen is proposed (Figure 10).

CO_2_ adsorbs on a bare CeO_2–*x*_ surface as M, B, and P carbonate species (only M and B are shown for clarity). Upon heating in the absence of hydrogen (red arrow, position 2), the M and B carbonates rearrange into the P carbonate or desorb as CO (minor pathway) or CO_2_ (major pathway).

In the presence of H species, bidentate and monodentate formate are formed (blue-green arrow, position 3). The hydrogenation of B carbonate to BF is shown above the blue-green arrow. With increasing temperature, two possibilities exist: (i) Under hydrogen-lean conditions (green arrows), the formates partially decompose to CO and CO_2_ and partially rearrange and hydrogenate into H_2_COO* intermediates (via hydrogen produced during formate decomposition, highlighted by the blue dashed arrow, position 4). The H_2_COO* group (position 4) can then further decompose to CO and H_2_O by weakening of the C–O bond in the carbonate species, arriving at position 7. (ii) In excess hydrogen, (blue arrows, where H* represents adsorbed hydrogen species which are present in excess), the formate readily converts to H_2_COO* and further to methoxy groups (attached via an oxygen atom to the cerium atom, positions 5 and 6). The methoxy can further hydrogenate to CH_4_ with byproducts of CO and H_2_O. This is supported by a DRIFTS-MS experiment, where a simultaneous decrease in bands characteristic of C–O and C–H stretching takes place together with the identification of methane gas. In parallel, MF and BF can also desorb as CO_2_ and H_2_O or convert to P carbonates (positions 4, 6, and 7). To further substantiate the conversion of methoxy to methane, Wang et al.^76^ observed that the hydrogenation of methanol to methane occurs easily over the Ru/CeO_2_ catalyst at 300 °C. Also, Kawi et al.^77^ observed that formate and methoxy species are important intermediates during CO_2_ hydrogenation to methane over Ni/CeO_2_ catalysts.

No decrease in the polydentate band intensity was detected during the injection of H_2_ pulses over the 2Ni–R catalyst at 500 °C (Figure 6D). This is in line with our experimental and DFT analyses of the polydentate carbonate binding strength on partially reduced ceria and likely also acts as a spectator during the DRM reaction. As a result, only a minor fraction of the ceria surface remains vacant to facilitate the dissociative CO_2_ activation at 500 °C (Table 1). The presence of hydrogen favors the transformation of monodentate and bidentate to CO, thus partially preventing their conversion to polydentate carbonate (Figures 6C and S16).

## 5 Conclusions

Nickel crystallites measuring about 1 nm in size were dispersed on ceria nanorods which predominantly expose {111} crystal facets. This produced an exceptionally active and stable DRM catalyst with low carbon accumulation, compared to ceria nanocube and nanosphere morphologies. This can be attributed to the synergetic actions of highly dispersed nickel for methane activation and the intermediate CO_2_ binding strength of the CeO_2_ nanorod surface for the stabilization of reactive M and B carbonates as well as high reducibility for facile CO_2_ dissociation. Catalytic stability is influenced by the terminating ceria facets: the jagged (100) surface present in ceria nanocubes helps maintain the structural integrity of nickel by preventing its sintering but causes severe coking due to poor redox activity.

CO_2_ adsorbs on (sub)stoichiometric ceria surfaces as monodentate, bidentate, and hydrogen carbonate species at room temperature. The polydentate carbonate population is favored over the {100} facet of ceria, which prevails in the ceria nanocube surface. On the contrary, monodentate and bidentate carbonates prevail on the {111} facet. The monodentate or bidentate species and the polydentate species on fully oxidized CeO_2_(111) are bound more weakly than on CeO_2_(100). The oxygen-deficient (partially reduced) ceria surfaces provide the strongest interaction with CO_2_, especially CeO_2_(100).

The most thermally stable polydentate (P) carbonate which covers a large fraction of the partially reduced ceria surface reacts sluggishly with H_2_, revealing P to be a spectator rather than an active species during the DRM reaction at 500 °C. The presence of hydrogen increases the reactivity of the surface carbonates and partially prevents their restructuring to inert polydentate, supporting the hydrogen-assisted CO_2_ activation pathway on Ni/CeO_2–*x*_. In a surplus of hydrogen, the dioxymethylene converts to methane and water via methoxy, whereas under H_2_-lean conditions, dioxymethylene preferentially decomposes to CO and water.

## Supporting Information Available

Additional explanations, kinetic analysis, catalytic performance comparison, carbon accumulation analysis, CO chemisorption, HR-TEM and SAED images, N_2_ physisorption data, XRD results, H_2_-TPR profiles, CO_2_ DRIFT spectra and temperature-dependent desorption profiles, XANES and EXAFS spectra and fitting results, ceria surface models, and DFT-calculated CO_2_ adsorption parameters (PDF)
