# Supplementary material for: CO2 Activation over Nanoshaped CeO2 Decorated with Nickel for Low-Temperature Methane Dry Reforming
Source: ACS Appl Mater Interfaces. 2022 Jul 8;14(28):31862–78. doi: 10.1021/acsami.2c05221 (PMC9305712; doi:10.1021/acsami.2c05221)
Supplement: Supplementary file 1 — am2c05221_si_001.pdf [file am2c05221_si_001.pdf]

Supporting Information

CO_2 activation over nanoshaped CeO_2 decorated with nickel for low-temperature methane dry reforming

Kristijan Lorber[1,2], Janez Zavašnik[3], Iztok Arčon[2,3], Matej Huš[1,4], Janvit Teržan[1], Blaž Likozar[1] and Petar Djinović[1,2]*

[1]National Institute of Chemistry, Hajdrihova 19, 1000 Ljubljana, Slovenia

[2]University of Nova Gorica, Vipavska 13, SI-5000 Nova Gorica, Slovenia

[3]Jožef Stefan Institute, Jamova cesta 39, SI-1000 Ljubljana, Slovenia

[4]Association for Technical Culture (ZOTKS), Zaloška 65, 1000 Ljubljana, Slovenia

Corresponding author e-mail: petar.djinovic@ki.si

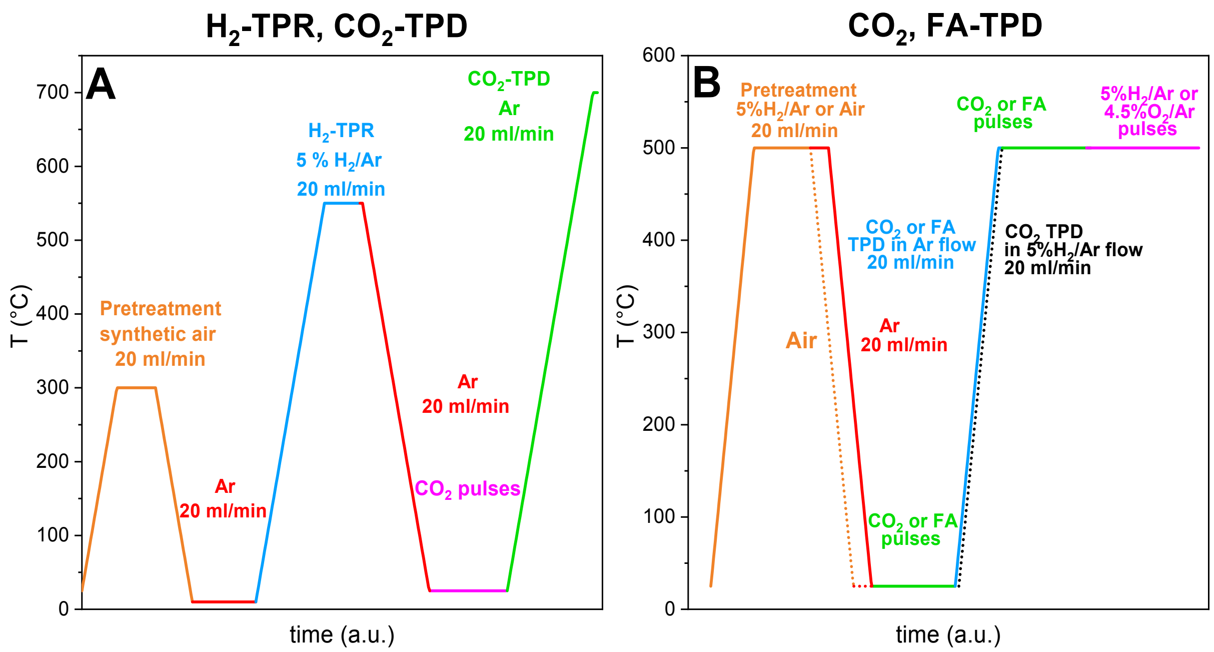

Figure S1. The analytical protocols for (A) H_2-TPR, CO_2-TPD and (B) FA and CO_2-TPD in Ar and 5% H_2/Ar flow.

Temperature programmed reduction with H_2 (H_2-TPR) and CO_2 desorption (CO_2-TPD)

Samples were pre-treated in a 20 ml/min flow of synthetic air (20% O_2/N_2, purity 5.0) at 300 °C for 30 min. After pre-treatment, the samples were cooled to 10 °C and the flow was switched to argon (20ml/min) for 10 min. An isopropanol-LN_2 cold trap (-90 °C) was installed to condense water vapor and remove interference with H_2 quantification (monitored by TCD detector). The H_2-TPR profile was recorded while the sample was heated to 550 °C with a ramp of 10 °C/min, in 5 % H_2/Ar flow (20 ml/min, purity 5.0). After reaching 550°C, the sample was kept at this temperature for 30 min to ensure that equilibrium reduction is achieved. After the H_2-TPR analysis, the atmosphere was switched to 20 ml/min of argon (purity 5.0) and the sample was cooled to 25 °C. The sample was saturated by consecutive CO_2 pulses (0.5 ml STP) until no more uptake was observed and dwelled at 25 °C for an additional 30 min to desorb any physisorbed CO_2. The saturated samples were heated in Argon to 700 °C with a ramp of 10 °C/min. The gas stream leaving the catalyst was continuously analyzed with a TCD detector and mass spectrometer (Pfeiffer Vacuum, model ThermoStar^{TM} GSD320).

Temperature programmed DRIFTS

For analysis, the reduced samples were heated with a ramp of 10 °C min^{-1} to 500 °C in 5 % H_2/Ar (purity 5.0) and held at this temperature for 1 h. The oxidized samples were pre-treated in synthetic air (20% O_2/N_2) with an identical thermal protocol. After a reductive or oxidative pretreatment, the samples were purged with Ar (purity 5.0) for 30 min at 500 °C and cooled to 25 °C. At 25 °C, four pulses (0.5 ml STP) of CO_2 (Linde, purity 5.3) were injected (Scenario A). For scenario B, where formate is the initial reaction intermediate,[1] formic acid (FA) vapors were generated by bubbling 20 ml/min of argon through a saturator filled with formic acid, which was thermostated at 70 °C and refluxed at 50 °C. After 4 injected pulses, the sample was degassed in pure argon (20 ml/min) for 20 min and heated at 10 °C/min to 500°C. For scenario C, CO_2 pulses were injected at 25 °C over the catalyst which was in a flow of 20 ml/min of 5% H_2/Ar, dwelled for 20 min to desorb weakly bound CO_2 and heated at 10 °C/min to 500°C.

For pulse isothermal experiments on activated 2Ni-R catalyst (see Fig. S17), three pulses of CO_2 or FA vapors (0.5 mL STP) were injected at 500 °C in argon flow (25 mL/min) (Scenarios A and B), or three pulses of CO_2 were injected into 5 % H_2/Ar flow (25mL/min) (Scenario C).

DRIFTS analysis was performed using ~10 mg of finely powdered catalyst, loaded into a porous ceramic cup and placed in the DiffusIR cell (PIKE Technologies) attached to a FTIR spectrometer (Perkin Elmer, model Frontier). The background was recorded with the powdered catalyst using a LN_2 cooled MCT detector. Spectra were recorded between 800 and 4000 cm^{-1}, 32 accumulations per scan and spectral resolution of 4 cm^{-1}. The outlet gas was analyzed with the mass spectrometer (Pfeiffer Vacuum, model ThermoStar™ GSD320).

1. Identification of experimental conditions ensuring operaton in kinetic regime

External mass transfer limitations. The reaction conditions that ensure catalytic experiments in the kinetic regime were experimentally tested at 500 °C with 2Ni-C, 2Ni-R and 2Ni-S catalysts. The samples were ground to a fine powder (aggregate size below 20 μm), thus ensuring absence of internal mass transfer limitations. Powdered catalysts (5 mg for 2Ni-R and 2Ni-C, 10 mg for 2Ni-S) were diluted with 20 mg of SiC and fixed between two quartz wool flocks. Prior to catalytic tests, samples were activated in 5 % H_2/N_2 stream of 15 ml/min at 500 °C for 1 h. To eliminate external mass transfer limitations, flowrates of CH_4 and CO_2 were stepwise increased from 10 to 40 ml/min and the corresponding methane conversion rate (rCH_4) was calculated based on the following equation:

$$xCH_4 = \frac{1-\left[{}^{C_{CH4\ out}}/_{C_{CH4\ in}}\right]}{1+F_{CH4}\left[{}^{C_{CH4\ out}}/_{C_{CH4\ in}}\right]}*100\% \quad (1)$$

$$F_{CH4}(\text{fractional change of volume}) = \frac{V_{X\,CH4=1} - V_{xCH4=0}}{VxCH4=0} = \frac{4-2}{2} = 1$$

$V_{xCH4=1}$=moles of reactants and products at complete methane conversion

$V_{xCH4=o}$=moles of reactants and products at zero methane conversion

$C_{CH4\ in}$= concentration of methane as measured by GC at zero methane conversion (blank)

$C_{CH4\ out}$= concentration of methane as measured by GC during reaction

CO_2 conversions were calculated in an analogous manner: CO_2 concentrations at the inlet and outlet of the catalytic reactor were used in Equation (1).

The H_2/CO ratio was calculated as follows:

$$H_2/CO = \frac{c_{H2}}{c_{CO}}$$

Whereas C_{H2} and c_{CO} are the concentrations of hydrogen and carbon monoxide, measured at the exit of reactor.

For more information on calculation of fractional change of volume and conversion calculation during varying volume reactions, the reader is referred to reference[2], Chapter 3, pages 67-70.

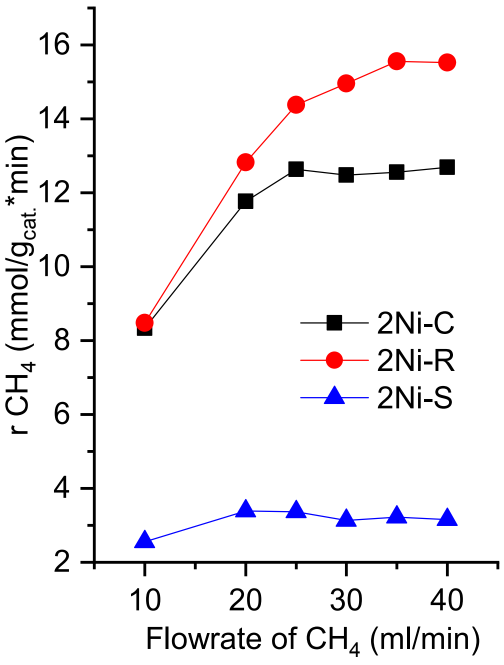

Figure S2. Experimental determination of kinetic regime through dependence of CH_4

reaction rate as a function of CH_4 flow for 2Ni-C, 2Ni-R and 2Ni-S catalysts at 500 °C.

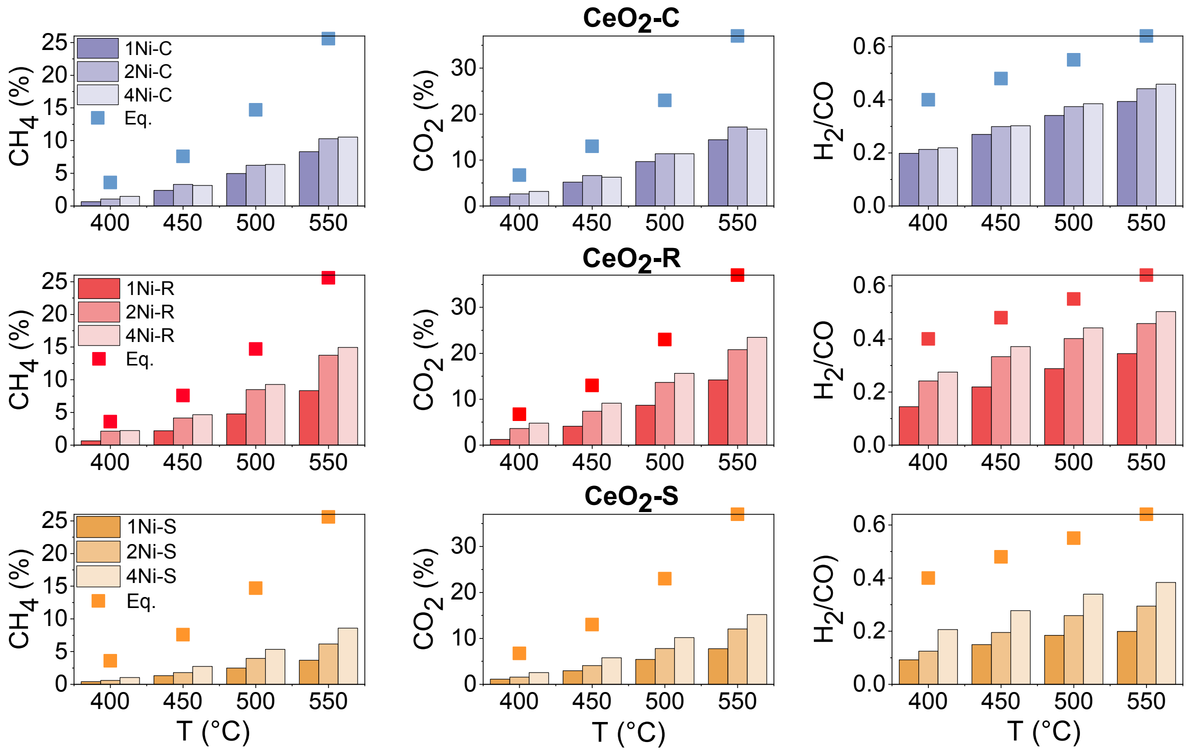

Figure S3. Results of preliminary catalytic tests showing CH_4 and CO_2 conversion, along with H_2/CO ratios achieved between 400 and 550 °C with Ni-R, Ni-C and Ni-S catalysts containing 1, 2 and 4 wt. % nickel. No catalytic activity was observed at these temperatures over bare nanoshaped CeO_2 supports, identifying the crucial role of nickel for the catalytic turnover. Squares represent equilibrium methane and CO_2 conversion as well as H_2/CO molar ratio, calculated for the equimolar CH_4/CO_2 feed ratio and 1bara pressure using Gaseq software.

Table S1. Comparison of catalytic performance of different Ni based DRM catalysts.

Catalyst composition	Reaction temperature and feed composition	CH_4 rate, mmol/g_{cat}*min	CO_2 rate, mmol/g_{cat}*min	H_2/CO, /	C selectivity, %	Source
2Ni/CeO_2-R	T=500 °C CH_4:CO_2=1:1	15.04	29.78	0.3	0.007	This work
2Ni/CeO_2-R	T=420 °C CH_4:CO_2=1:1	1.94	6.07	0.14	N.A.	This work
2Ni/CeO_2-C	T=500 °C CH_4:CO_2=1:1	12.49	22.31	0.33	0.07	This work
2Ni/CeO_2-C	T=420 °C CH_4:CO_2=1:1	1.56	3.59	0.15	N.A.	This work
2Ni/CeO_2-S	T=500 °C CH_4:CO_2=1:1	3.35	6.69	0.23	0.004	This work
2Ni/CeO_2-S	T=420 °C	0.33	1.12	0.07	N.A.	This work

	CH_4:CO_2=1:1					
25Ni/$MgAlO_x$	T=550°C CH_4:CO_2=1:1	1.00	0.89	0.8	0.007	[3]
10Ni/Al_2O_3-CeO_2	T=550 °C CH_4:CO_2=1:1	4.46	4.91	0.64	N.A.	[4]
1Ni/SiO_2	T=500 °C CH_4:CO_2=2.2:1.8	0.29	0.43	0.4	N.A.	[5]
0.5Pt-8Ni/$MgCe_{0.6}Zr_{0.4}O_2$	T=454 °C CH_4:CO_2=1:1	0.18	0.18	0.23	0	[6]
5Ni/γ-Al_2O_3	T=500 °C CH_4:CO_2=1:1	0.24	0.30	0.77	0.04	[7]
10Ni-MnO_x-ZrO_x/SiO_2	T=500 °C CH_4:CO_2=1:1	0.96	1.26	0.64	1.24	[8]
10Ni-MnO_x-ZrO_x/SiO_2	T=400 °C CH_4:CO_2=1:1	0.12	0.26	0.56	0.70	[8]
10Ni-Zr/SiO2	T=450 °C CH_4:CO_2=1:1	0.35	0.49	0.61	N.A.	[9]
10Ni-Zr/SiO_2	T=400 °C CH_4:CO_2=1:1	0.01	0.01	0.67	N.A.	[9]
20Ni/$MgAlZrO_x$-CeO_2	T=550 °C CH_4:CO_2=1:1	0.71	0.83	0.83	0.1	[10]
15Ni/La-$MgAlO_x$	T=550 °C CH_4:CO_2:Ar=1:1:8	0.95	1.19	0.78	2.45	[11]
18Ni/$CeAlMgO_x$	T=550 °C CH_4:CO_2:Ar=1:1:8	1.02	1.05	0.95	3.27	[12]
59Ni/$AlCeO_x$	T=550 °C CH_4:CO_2:Ar=1:1:8	1.40	1.02	1.70	4.90	[12]
7.8Ni-Si/ZrO_2	T=400 °C CH_4:CO_2=1:1	0.23	0.20	0.67	0.07	[13]
7.8Ni-Si/ZrO_2	T=450 °C CH_4:CO_2=1:1	0.64	0.60	0.70	0.18	[13]
19Ni/$MgAlO_x$	T=550 °C CH_4:CO_2=1:1	0.25	0.27	0.88	N.A.	[14]
64Ni/Al_2O_3	T=550 °C CH_4:CO_2=2:1	0.59	0.33	1.05	N.A.	[15]
7Ni/$MgAlCeO_x$	T=550 °C CH_4:CO_2=1:1	0.17	0.21	0.74	N.A.	[16]
5Ni-CaO/ZrO2-La2O3	T=450 °C CH_4:CO_2=1:1	0.44	0.58	0.67	N.A.	[17]
11NiSc/Al_2O_3	T=450 °C CH_4:CO_2=1:1	0.22	0.27	0.79	0.25	[18]
6.8Ni/SiO2	T=450 °C CH_4:CO_2=1:1	0.2	0.44	0.25	0.11	[19]

N.A.= Not analyzed

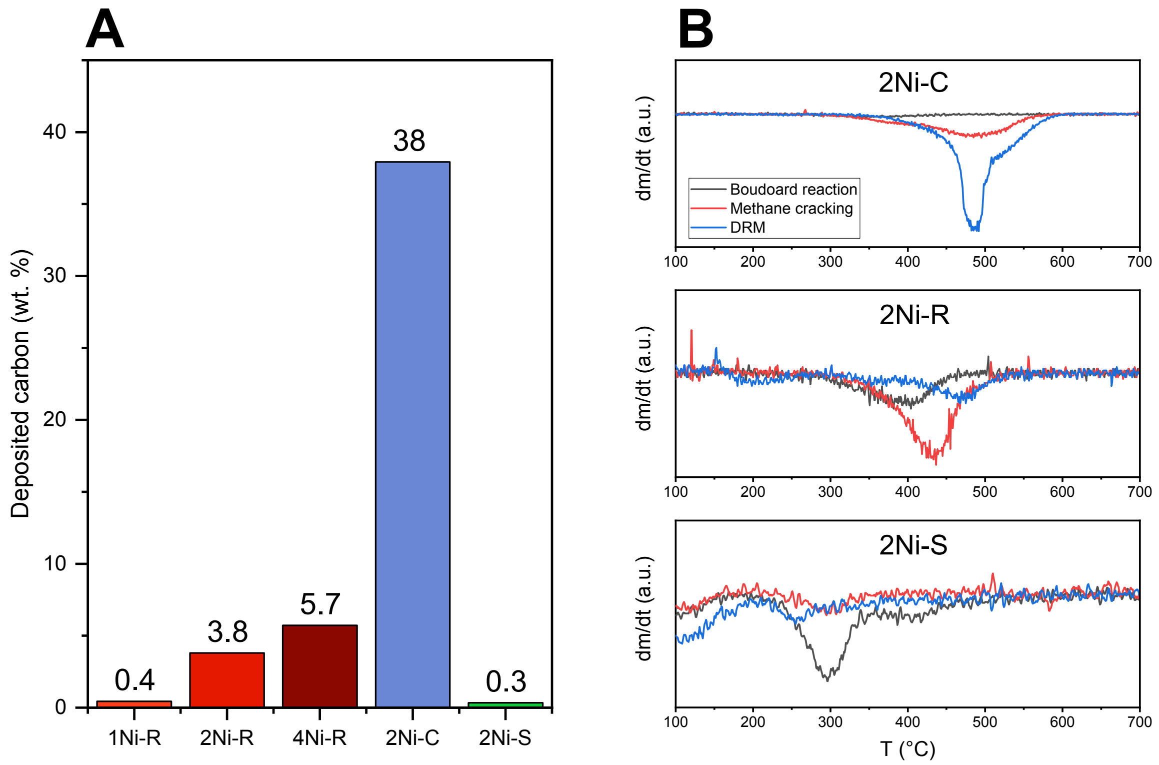

Figure S4. (A) Carbon deposition during 6h DRM reaction on different CeO_2 morphologies and different loadings of Ni content on CeO_2 nanorods morphologie. (B) Oxidation of carbon accumulated during DRM, methane cracking and Boudouard reaction as function of temperature.

To evaluate the reactivity of the carbon which accumulated during DRM, methane cracking and the Boudouard reaction, the 2Ni-R, 2Ni-C, and 2Ni-S samples were analyzed. The catalysts were first activated in 5 % H_2/Ar for 1h at 500 °C followed by exposure to either equimolar CH_4-CO_2 (WHSV = 180 L/g_{cat}*h), pure CH_4, or a CO flow (WHSV = 120 L/g_{cat}*h), for 1 h. The reactivity of carbon was analyzed using a TGA-TPO technique (Perkin Elmer, model STA6000) by heating the samples to 800 °C in air (25 mL/min) at a rate of 10 °C/min.

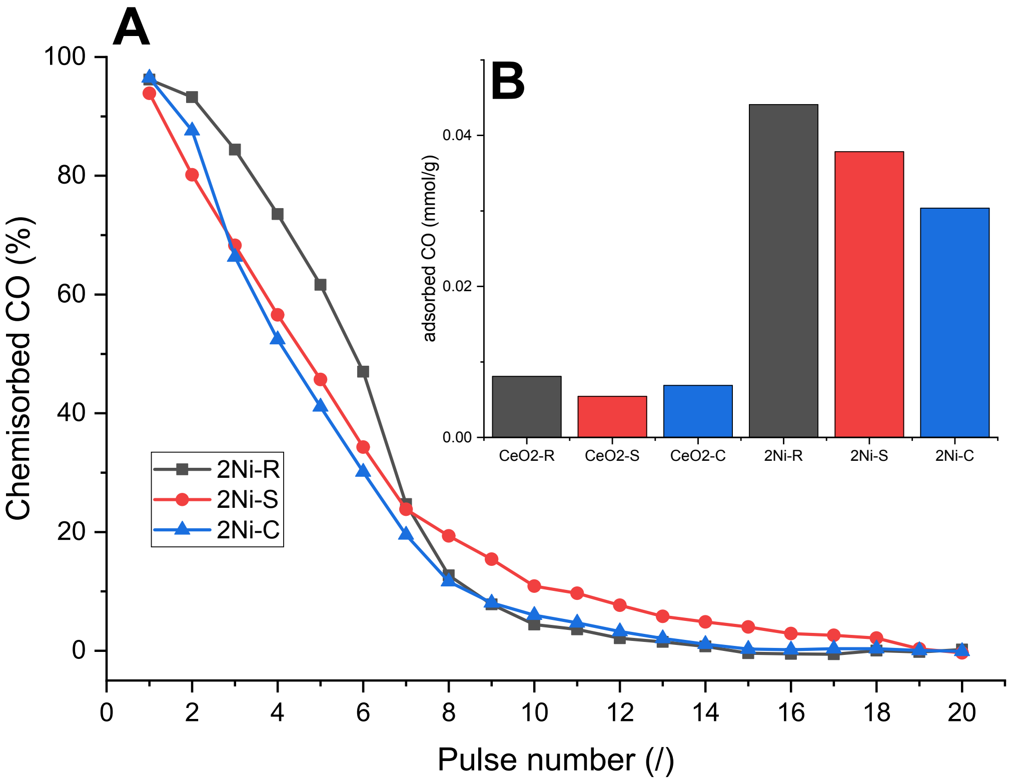

Figure S5. CO chemisorbed during pulsing at -10 °C (A) on 2Ni-R, 2Ni-C and 2Ni-S catalysts and (B) total chemisorbed CO for reduced bare ceria supports and 2Ni-R, 2Ni-C and 2Ni-S catalysts. Experients were performed in Autochem 2920 apparatus using 100mg of powdered catalysts, which were *in-situ* reduced at 500°C for 30 min in 5% H_2/Ar flow of 20 ml/min. Afterwards, the samples were cooled in Argon flow to -10°C, where 20 CO pulses (0.5 ml of 5%CO/He) were injected.

The purpose of dynamic CO chemisorption was to analyze accessibiliy of nickel sites. For 2Ni-R and 2Ni-C catalysts which are mesoporous, the cumulative CO adsorption reaches a steady state faster i.e. after 8 pulses. On the other hand, the CO chemisorption on 2Ni-S tails considerably, indicating kinetically hindered accessibility of active sites for CO. This is consistent with microporous structue of this sample and hindered mass transfer. This is a strong evidence revealing the origin of low catalytic activity of 2Ni-S catalyst.

2. Transmission Electron Microscopy (TEM) analysis

2.1 Nickel on ceria nanocubes (2Ni-C catalyst)

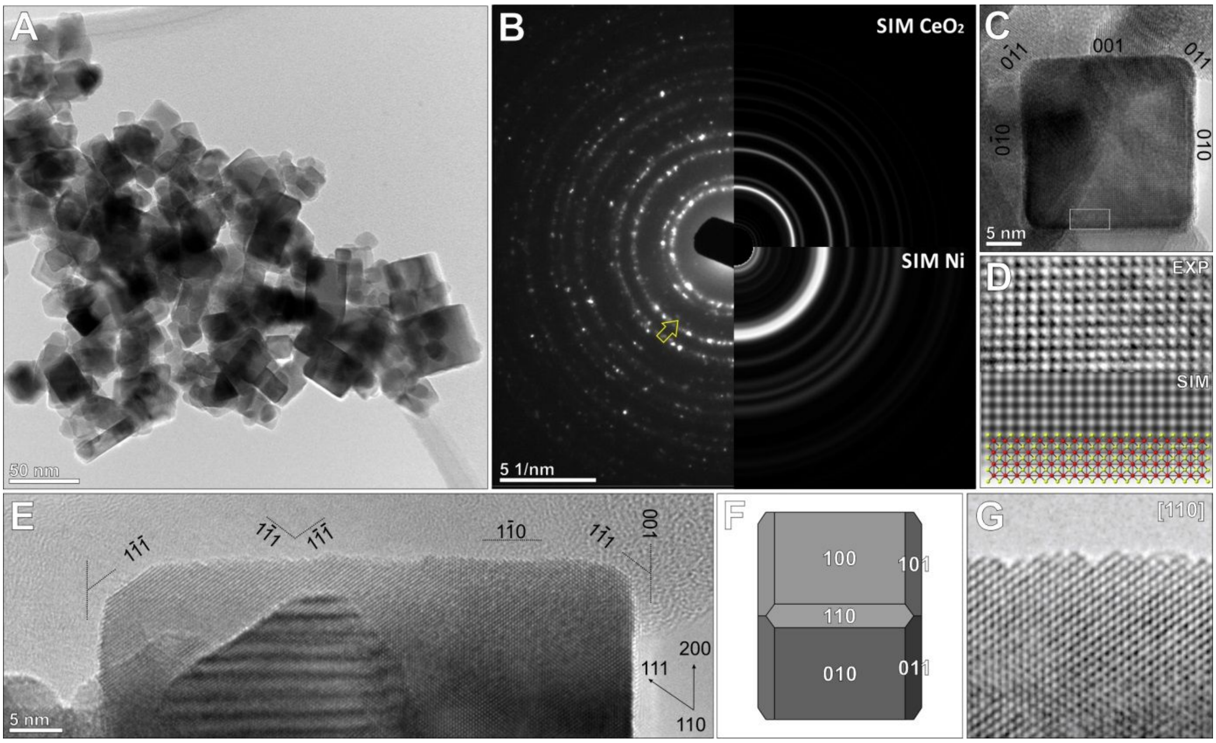

Figure S6. (A) overiview of CeO_2 cubes, decorated with 2 wt.% Ni NPs, with (B) corresponding SAED experimental ring pattern, recorded over the larger agglomerate (characteristic weak diffractions of metallic Ni are marked with yellow arrow); experimental pattern is supplemented with calculated simulations for cubic CeO_2 and Ni. (C) CeO_2 cube in [100] zone axis, with marked crystal faces. The region marked with white square is enlarged in (D), HR-TEM micrograph (EXP), and supplemented by HR-TEM simulation (SIM) with superimposed structure model. (E) CeO_2 cube in [110] zone axis, with marked steps on the (110) edge, and (F) model in corresponding orientation. (G) is enlarged HR-TEM micrograph of individual <111> steps as observed on the (110) edge.

2.2 Nickel on ceria nanorods (2Ni-R catalyst)

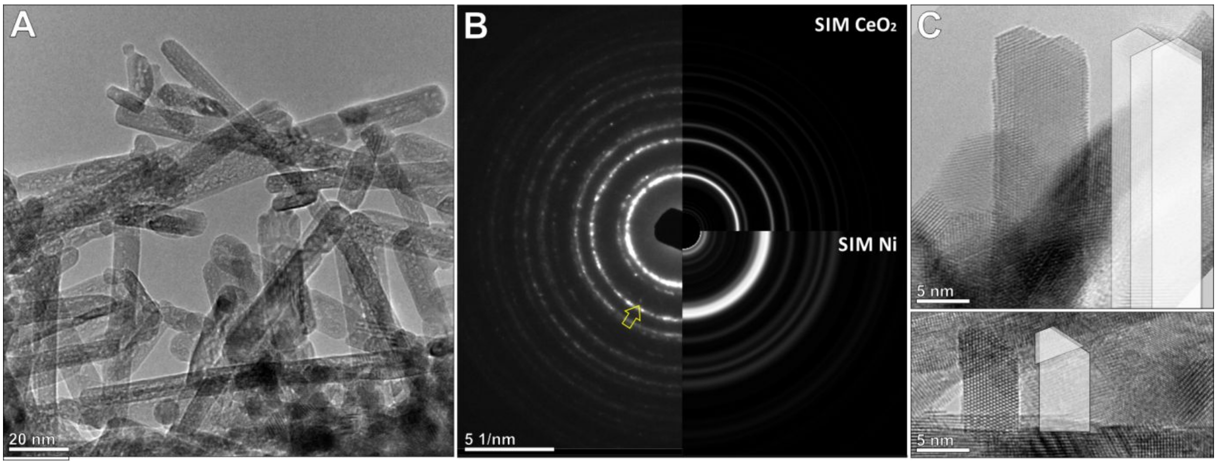

Figure S7. (A) overiview of CeO_2 nanorods, decorated with 2 wt.% Ni NPs (B) corresponding SAED experimental ring pattern, recorded over the larger agglomerate (characteristic weak diffractions of metallic Ni are marked with yellow arrow); experimental pattern is supplemented with calculated simulations for cubic CeO_2 and Ni and (C) HR-TEM micrograph showing parallel growth of CeO_2 rods, with added model representation.

2.2 Nickel on ceria nanospheres (2Ni-S)

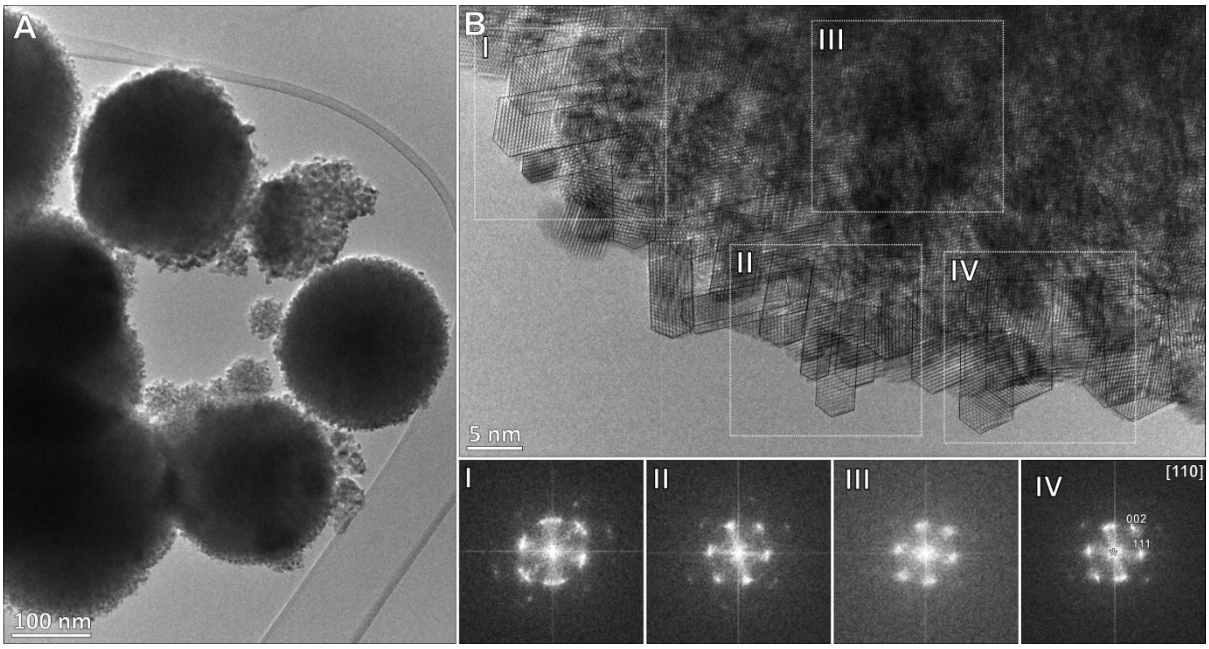

Figure S8. (A) overiview TEM micrograph of 2Ni-S catalyst showing spherical shape of agglomerates. (B) Phase contrast HR-TEM micrograph of sphere's edge, with outlined individual CeO_2 crystallites showing prominent {111} facets. The Fast fourier transform (FFT) patters (I-IV) from corresponding regions corresponds to minute missalignment of the crystallites; grains from region I which appear 90° rotated compared to grains from region II or IV have different morphology, but internal structure in very similar orientation.

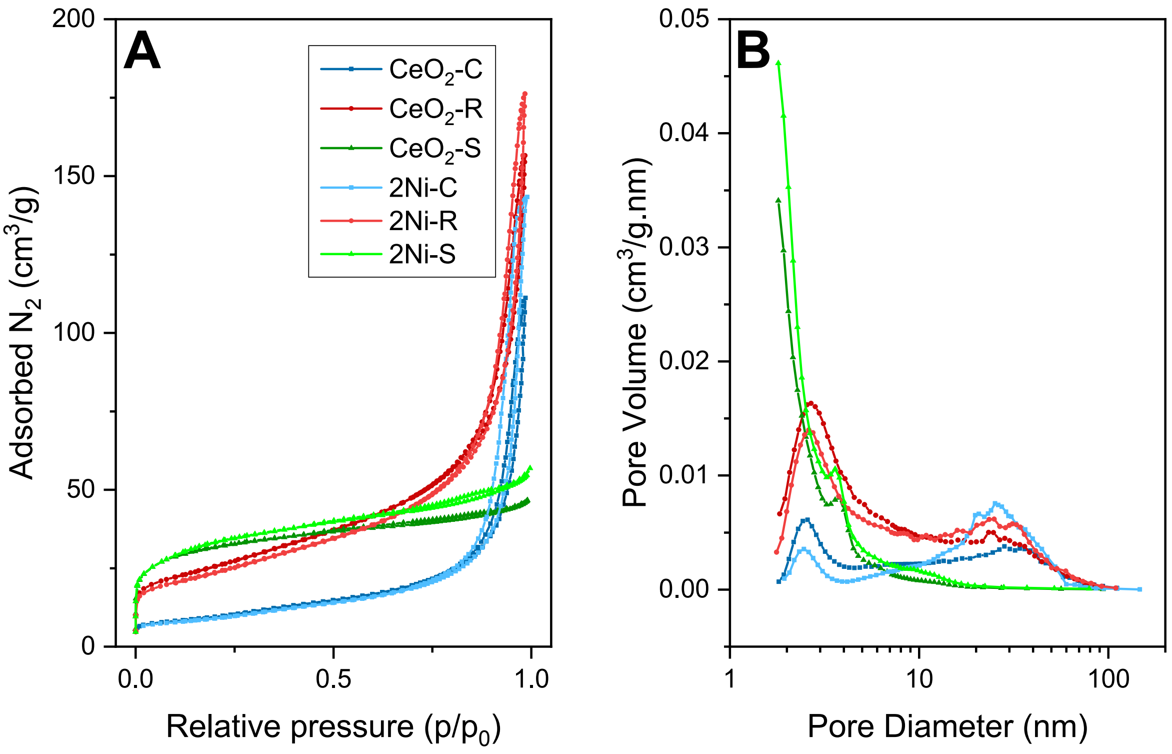

Figure S9. (A) N_2 physisorption isotherms and (B) pore size distribution for CeO_2 supports before and after deposition of 2 wt.% nickel.

Table S2. Specific surface area, total pore volume and average pore size diameter for different CeO_2 morphologies before and after deposition of 2 wt. % nickel.

Sample	S_{BET}(m^2/g)	V_{pore}(cm^3/g)
CeO_2-R	90	0.25
2Ni-R	84	0.28
CeO_2-C	35	0.17
2Ni-C	33	0.22
CeO_2-S	103	0.06
2Ni-S	111	0.07

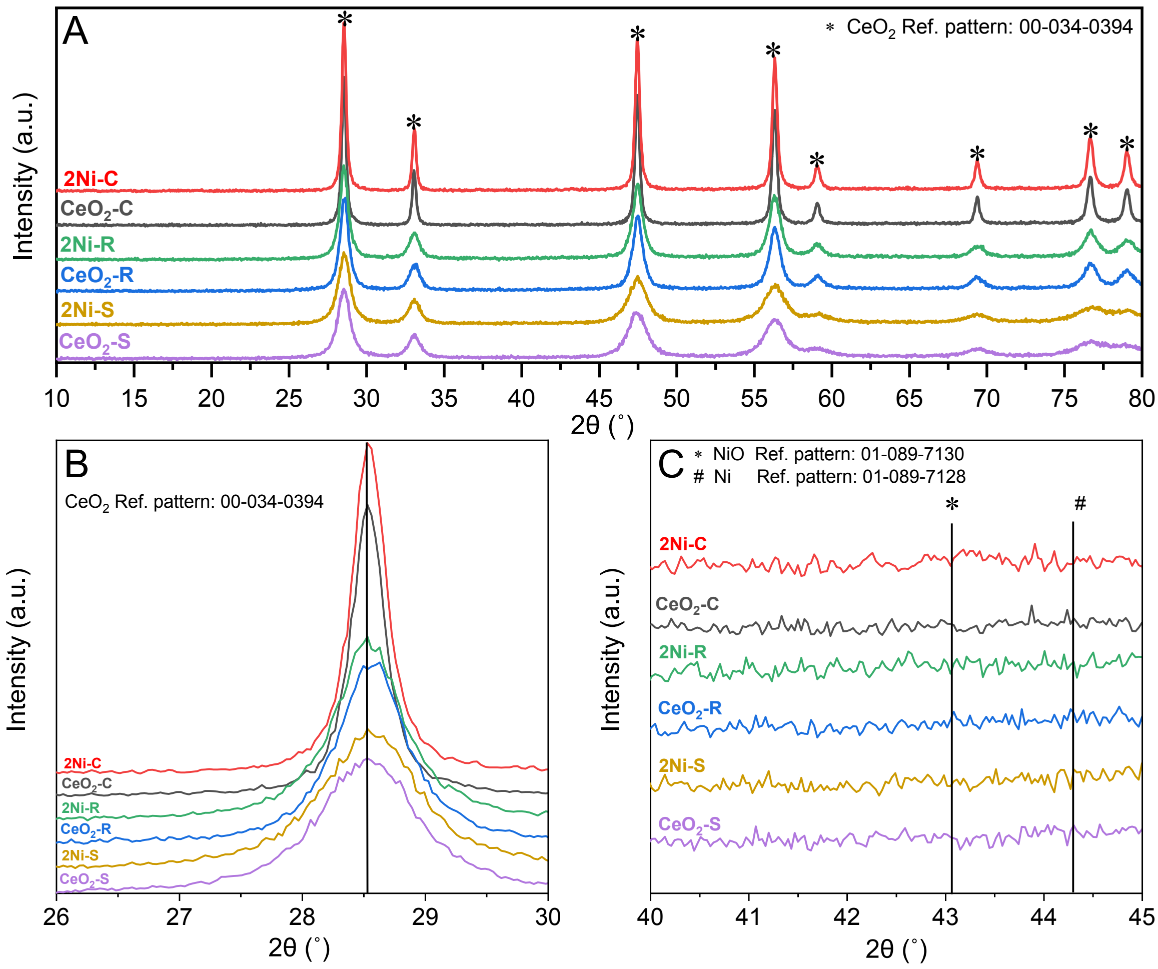

Figure S10. (A) XRD diffraction patterns of bare CeO_2 nanorods and 2Ni/CeO_2 catalysts, (B) magnification of the CeO_2 [111] peak region and (C) magnification of the 2 theta region where the most intensive diffraction from NiO[200] and Ni [111] (cubic, Fm-3m phase) is expected.

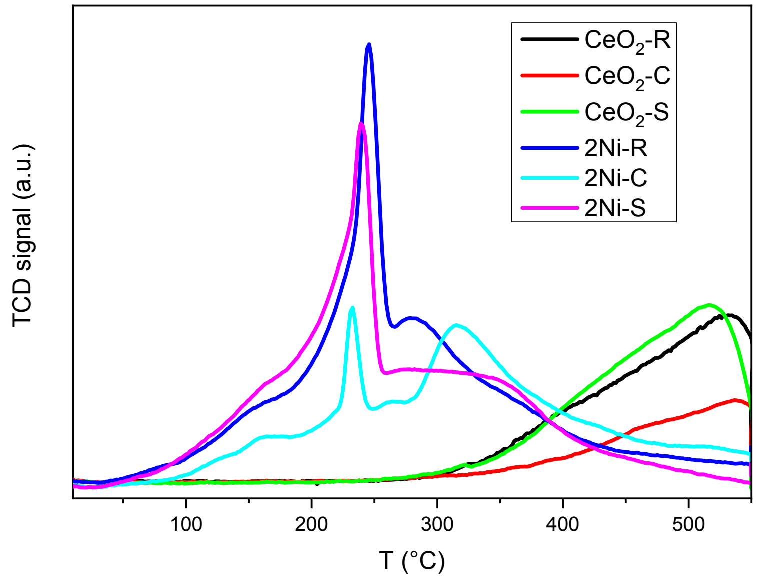

Figure S11. H_2-TPR profiles for different CeO_2 morphologies with and without deposited Ni.

The methodology for Ce^{3+} estimation during H_2-TPR analysis

The H_2 *tot* (ml/g) amount is obtained from the integration of the H_2-TPR signal using a suitable calibration.
The H_2 *tot* contains contributions from reduction of NiO to Ni (H_2 *Ni*) and CeO_2 to Ce_2O_3 (H_2 *Ce*), which occur simultaneously.
The exact sample mass during the H_2-TPR analysis is known, as is the exact Ni content (ICP OES, see Experimental section). We calculated the (H_2 *Ni*), required for reduction of 95% (2Ni-Rand 2Ni-C) or 90% (2Ni-S) of nickel oxide to metallic nickel, according to Fig. 7A at 550°C. For bare CeO_2 samples, the H_2 *Ni* is zero.
The difference between (H_2 *tot* - H_2 *Ni*) is the amount of hydrogen that remains for reduction of ceria (H_2 *Ce*). The ceria reduction stoichiometry is the following: $2CeO_2 + H_2 = Ce_2O_3 + H_2O$
As a result, the amount of oxygen removed from ceria can be calculated, and thus the fraction of Ce^{3+}.

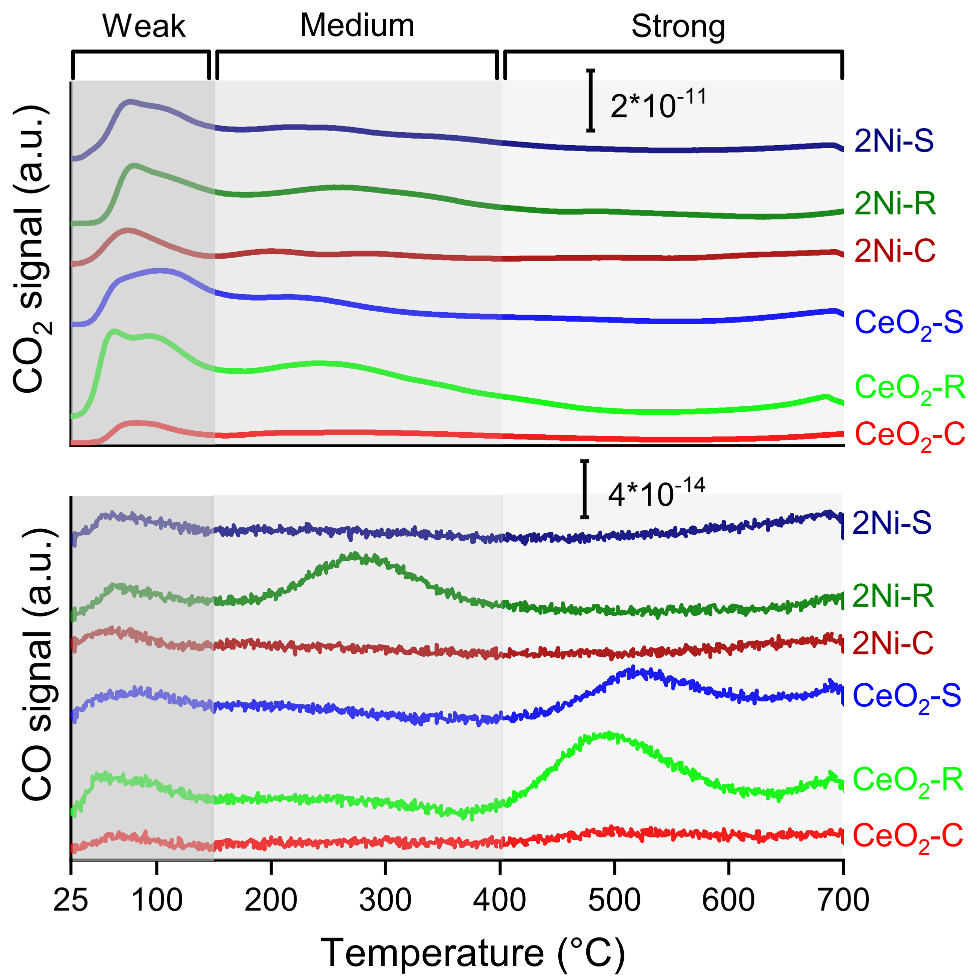

Figure S12. The CO_2-TPD (Temperature Programmed Desorption) showing evolution of CO_2 (m/z= 44) and CO (m/z= 29) as a function of temperature for bare reduced CeO_2 supports and 2 wt. % nickel containing catalysts. Weak, medium and strong is related to strength of CO_2 adsorption. Spectra are offset vertically for clarity. Despite the m/z=29 ion being common to both CO and CO_2 during electron ionization of the mass spectrometer[20], the dissimilarity in CO_2 (m/z= 44) and CO (m/z= 29) traces strongly confirms formation of CO.

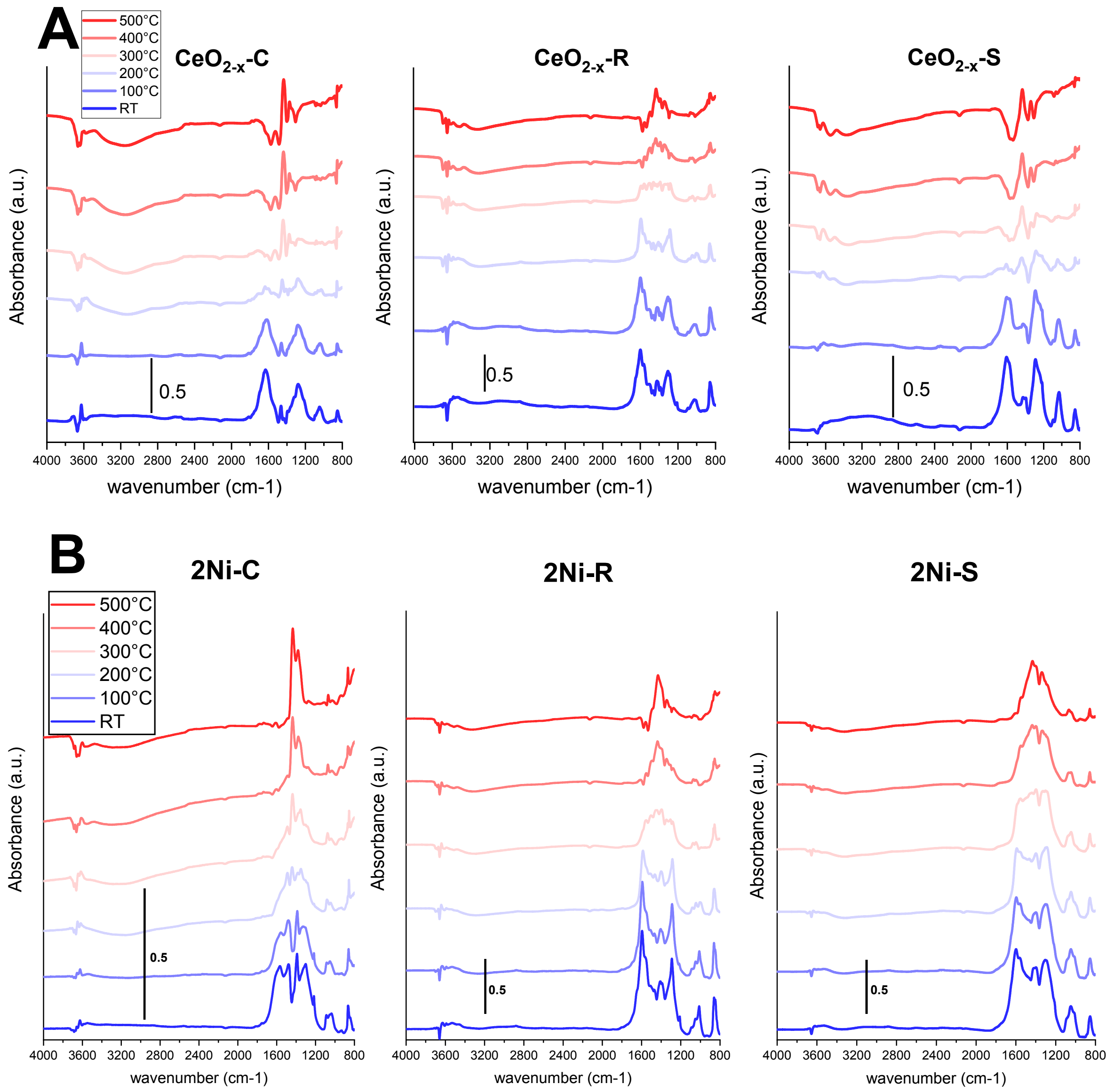

Figure S13. (A) DRIFT spectra of bare CeO_{2-x}-C, CeO_{2-x}-R and CeO_{2-x}-S and (B) 2Ni-C, 2Ni-R and 2Ni-S catalysts. Spectra were recorded after sample reduction in 5% H_2 at 500 °C for 30 min, cooling in argon to 25°C and CO_2 adsorption at 25 °C, followed by ramping to 500 °C.

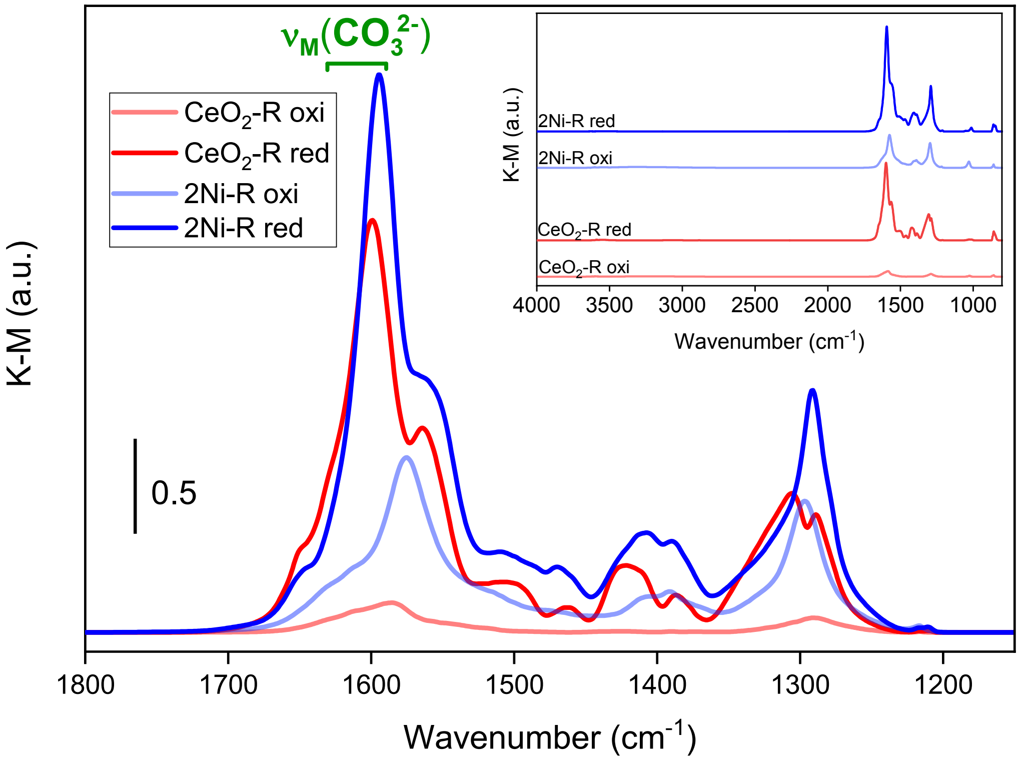

Figure S14. CO_2 adsorbed at 25 °C on reduced and oxidized CeO_2-R and 2Ni-R samples, highlighting the characteristic region where carbonate vibrations occurr. Inset shows the entire analyzed range.

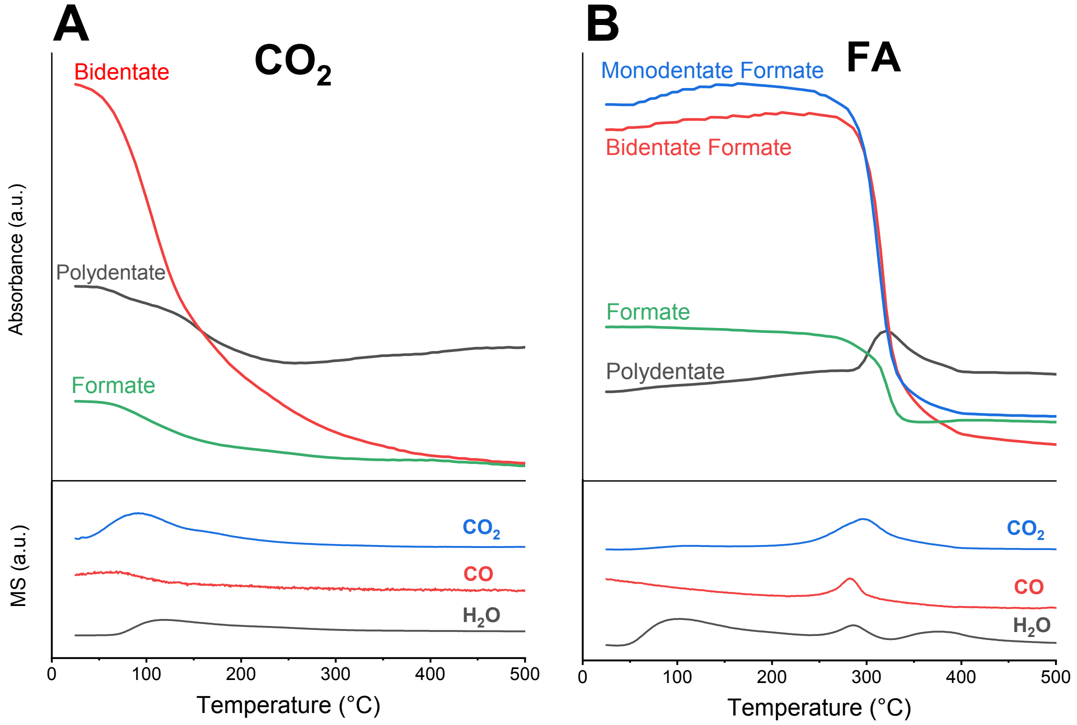

Figure S15. Intensity profiles of characteristic IR bands during (A) CO_2-TPD and (B) FA-TPD for oxidized 2Ni-R sample (upper panel). MS analysis of evolved gases (bottom panel).

Assignation of carbonate vibrations with formic acid as probe molecule

Due to complexity and overlapping contributions of individual vibrations in the obtained CO_2-DRIFTS spectra (see manuscript, Fig. 5), reliable peak assignation and identification of carbonate species is not trivial. Formic acid (FA) was used as a probe molecule, due to its signature IR peaks for carboxylate vibrations: 1650 - 1540 cm^{-1} and 1450 - 1360 cm^{-1} for v_{as} (COO^-) and v_s (COO^-), respectively (Fig. S16). In order to assign COO^- vibrations to carboxylate, both asymmetric and symmetric vibrations need to be present at the same time. Moreover, after FA adsorption, the vibrations between 2950 cm^{-1} and 2700 cm^{-1} appear, characteristic for aldehydic (C-H) bond stretching.[21] When FA vapors were adsorbed on 2Ni-R samples, signature peaks appeared between 1360 - 1450 cm^{-1}, 1540 – 1650 cm^{-1} and 2700 – 2950 cm^{-1}, identifying symmetric and asymmetric COO^- vibrations as well as C-H stretching and bending vibrations, clearly identifiable as formate groups. The mentioned peaks are present between 25 and 200 °C on oxidized as well as reduced 2Ni-R. The presence of (C-H) vibrations suggests that formate is adsorbed via O to Ce^{4+} and/or Ce^{3+} and not through C, as that would require C-H bond breaking and absence of 2800 – 2950 cm^{-1} vibrations. The presence of (COO^-) vibrations, as well as peaks at 1600 cm^{-1} and 1550 – 1570 cm^{-1} suggests that adsorbed FA on CeO_2 surface between 25 and 200 °C exists as monodentate formate and bidentate formate, respectively.[22–24]

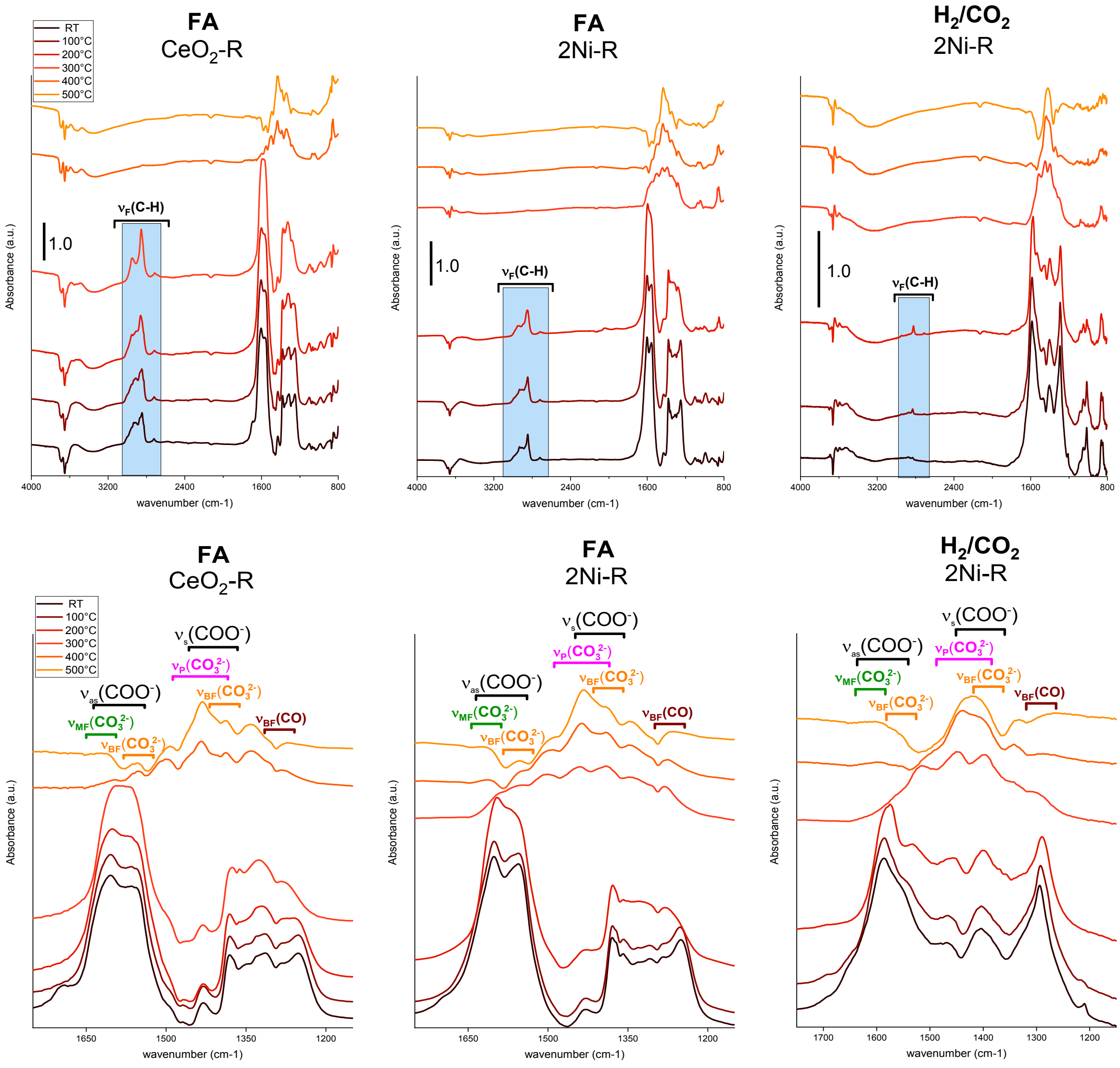

Figure S16. DRIFT spectra of temperature programmed desorption of formic acid (FA) in argon flow, or adsorbed CO_2 in 5% H_2/Ar flow from reduced CeO_2-R and 2Ni-R samples. Entire spectra are shown in the upper row, bottom row shows characteristic region where carbonate vibrations occur. Spectra are shifted vertically for clarity.

Isothermal pulse experiments

The interaction of the reduced 2Ni-R catalyst with pulses of CO_2 and FA vapors in inert argon atmosphere, as well as CO_2 pulses in diluted H_2 atmosphere was analyzed at a catalytically relevant temperature of 500 °C. The analysis of the gas phase during CO_2 pulsing in argon atmosphere showed that the CO/CO_2 ratio of the desorbed gases is 0.18 (Fig. S17, blue bar columns). This indicates that, at 500 °C, CO_2 partly disassociates to CO and lattice oxygen, leading to the re-oxidation of CeO_{2-x}. It additionally causes enrichment of the surface with polydentate carbonates (Fig. S17 orange spheres).

FA pulses at 500°C result in a rise of the polydentate carbonate coverage in a similar amount as with CO_2 pulses, while the gaseous CO/CO_2 ratio reaches 0.83. Water is also desorbed, which is consistent with temperature programmed dissociation of formic acid (see manuscript Fig. 6B).

During CO_2 pulses in a 5% H_2/Ar flow at 500°C, the desorbed CO/CO_2 ratio is 0.32 and substantial amounts of water and methane are formed. This is consistent with the results of the temperature programmed experiment where in an excess of hydrogen, methanation is favored over the RWGS reaction, see manuscript Fig. 6C).

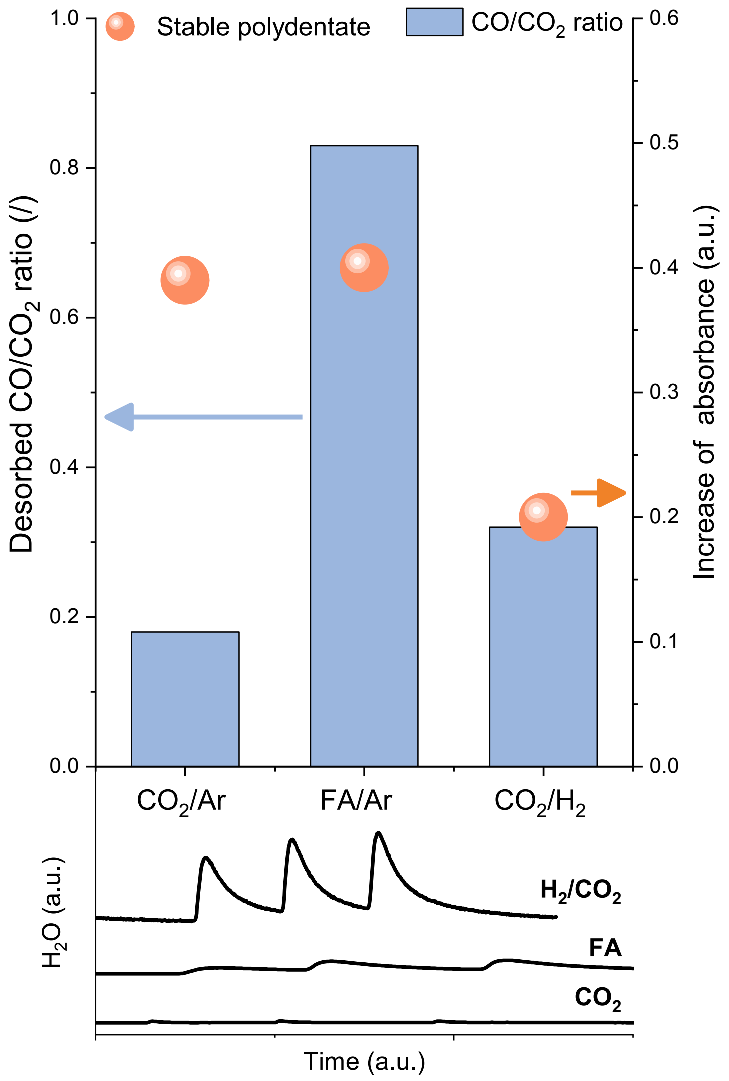

Figure S17. The gaseous CO/CO_2 ratio (blue columns) and the increase in the polydentate carbonate band intensity (and at 1440 - 1420 cm^{-1}, orange spheres) during CO_2 or FA pulses in an argon flow, and CO_2 pulses in 5 % H_2/Ar flow at 500°C, over a reduced 2Ni-R catalyst. Water detection (analyzed with MS, *m/z*= 18) in each pulse is shown in the bottom panel.

3. Ni K-edge XANES analysis

The experiments were performed at the P65 beamline of PETRA III, DESY, Hamburg, Germany and at the XAFS beamline of the ELETTRA synchrotron radiation facility in Trieste, Italy. A Si(111) double crystal monochromator was used with energy resolution of about 1 eV at 8 keV. At the P65 beamline higher-order harmonics were effectively eliminated by the flat mirror installed in front of the monochromator. The beam size on the sample was 1.5 mm horizontal and 0.2 mm vertical. At XAFS beamline the higher harmonics were eliminated by detuning of the second monochromator crystal to 60% of the maximum in the rocking curve, and the size of the beam on the sample was set to about 1 mm × 3 mm. The intensity of the monochromatic X-ray beam was measured by three consecutive ionization detectors, filled with appropriate nitrogen and argon gas mixtures to obtain 15% absorption in the first cell and 70% in the second and third cell.

The catalyst samples were prepared in the form of homogeneous pellets, pressed from micronized sample powder mixed with BN powder, with the total absorption thickness (μd) of about 2.5 above the Ni K-edge. The sample pellets, closed in a tubular oven reactor (Carbolite MTF 12/25/400) with 20 micron aluminum foil windows, were placed in the monochromatic beam between first two ionization detectors.

The absorption spectra were measured in the energy region from -150 eV to +1000 eV relative to the Ni K-edge. At XAFS beamline the spectra were measured with 0.25 eV steps in the XANES region and equidistant k steps of 0.03 $Å^{-1}$ in EXAFS region, with an integration time of 1 s/step. At P65 beamline fast (3 min) continuous scans were performed and re-binned to the same energy steps as at XAFS beamline. At each intermediate stationary state several repetitions of the scans were measured and superimposed to improve the signal-to-noise ratio.

In-situ Ni K-edge XANES analysis is used to monitor changes in valence of Ni cations after reduction in 5% H_2/N_2 flow for 1h at 400 °C, and during DRM reaction in equimolar CO_2/CH_4 flow at 400°C, 500 °C and 550 °C. Normalized Ni K-edge XANES spectra of the 2Ni-R, 2Ni-C, 2Ni-S and 4Ni-R catalysts, measured *in-situ* are presented in Figures S18-S21, together with the spectra of corresponding Ni reference compounds (crystaline NiO and Ni metal).

Linear combination fit (LCF) analysis of the in-situ XANES is used to determine the relative amounts of Ni^{2+} and Ni metal species in the catalyst after reduction and during DRM reaction. All in situ XANES spectra can be completly described by a linear combination of two reference XANES profiles: the spectrum of crystaline NiO as reference for Ni^{2+} and the spectrum of 2Ni-R catalyst measured after DRM reaction at 550°C as reference for Ni metal nanoparticles (with small amount, about 5%, of Ni^{2+}). Examples of LCF analysis are shown on Figures S23-S25.

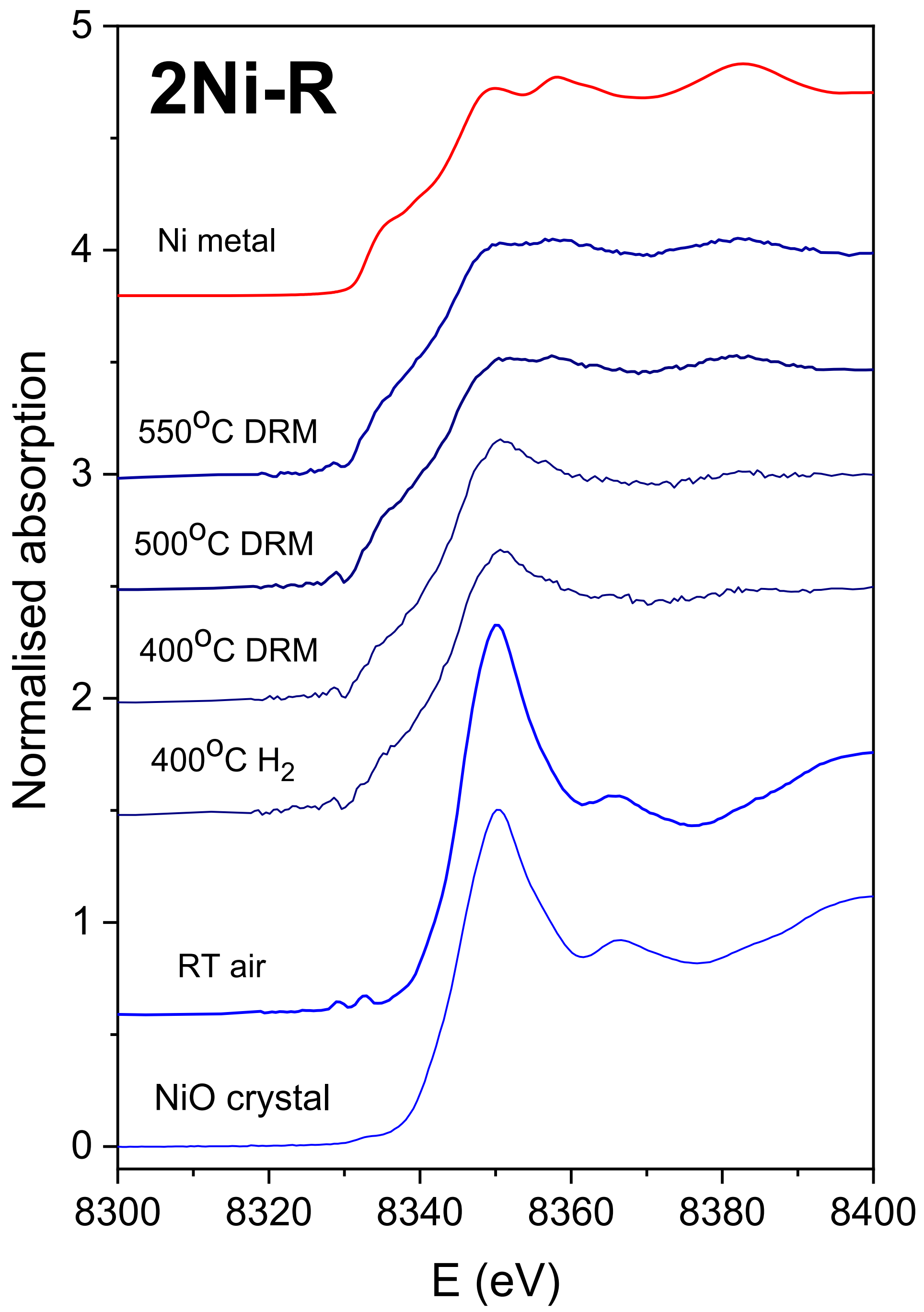

Figure S18. Normalized Ni K-edge XANES spectra measured in situ on the 2Ni-R catalyst measured in-situ at RT in air, after reduction in 5% H_2/N_2 flow for 1h at 400 °C, and during DRM reaction in equimolar CO_2/CH_4 flow at 400 °C, 500 °C and 550 °C**.** The spectrum of Ni metal and the spectrum of crystalline NiO, as a reference for Ni^{2+}, are shown for comparison.

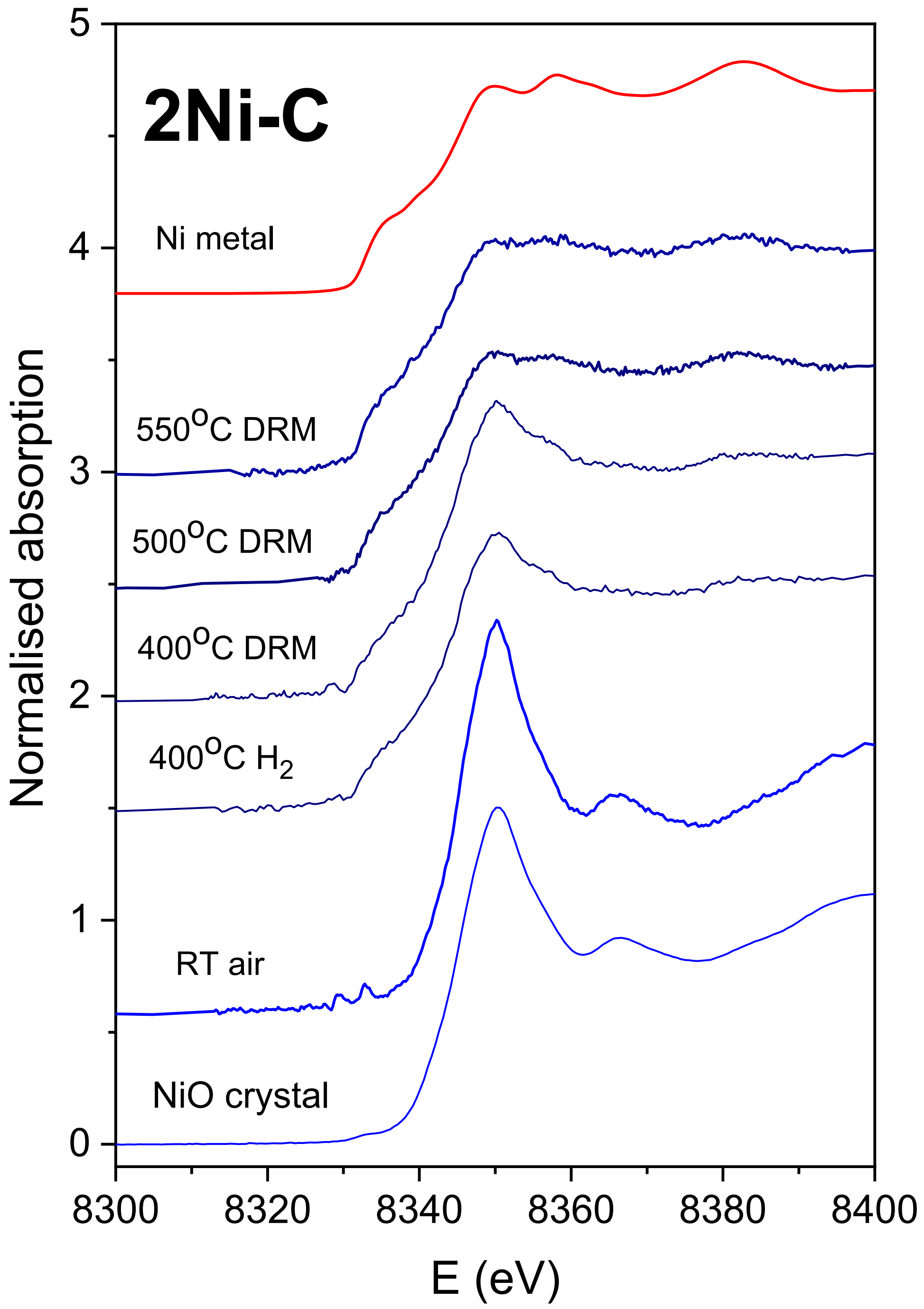

Figure S19. Normalized Ni K-edge XANES spectra measured in situ on the 2Ni-C catalyst measured in-situ at RT in air, after reduction in 5% H_2/N_2 flow for 1h at 400 °C, and during DRM reaction in equimolar CO_2/CH_4 flow at 400 °C, 500 °C and 550 °C. The spectrum of Ni metal and the spectrum of crystalline NiO, as a reference for Ni^{2+}, are shown for comparison.

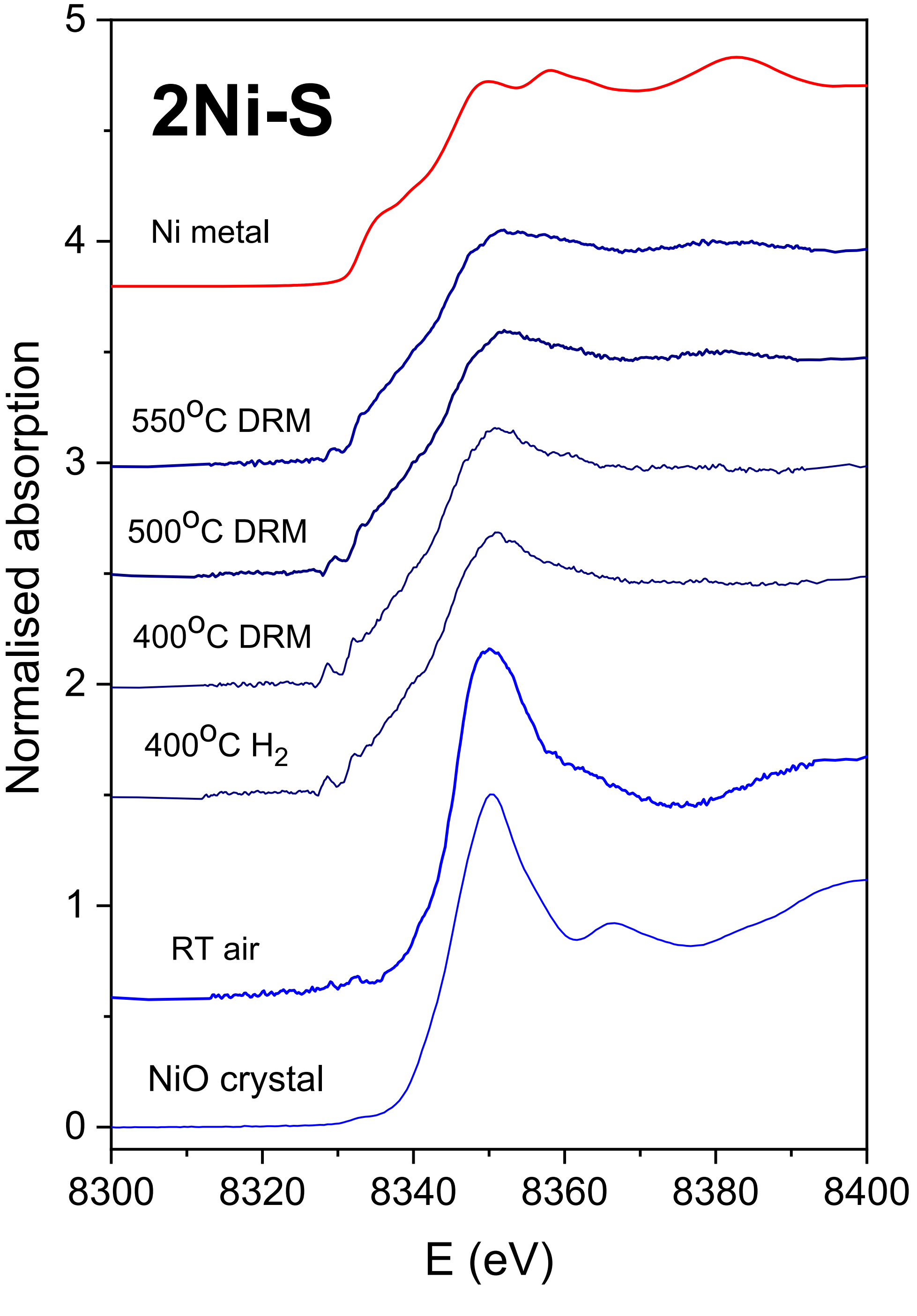

Figure S20. Normalized Ni K-edge XANES spectra measured in situ on the 2Ni-S catalyst measured in-situ at RT in air, after reduction in 5% H_2/N_2 flow for 1h at 400 °C, and during DRM reaction in equimolar CO_2/CH_4 flow at 400 °C, 500 °C and 550 °C**.** The spectrum of Ni metal and the spectrum of crystalline NiO, as a reference for Ni^{2+}, are shown for comparison.

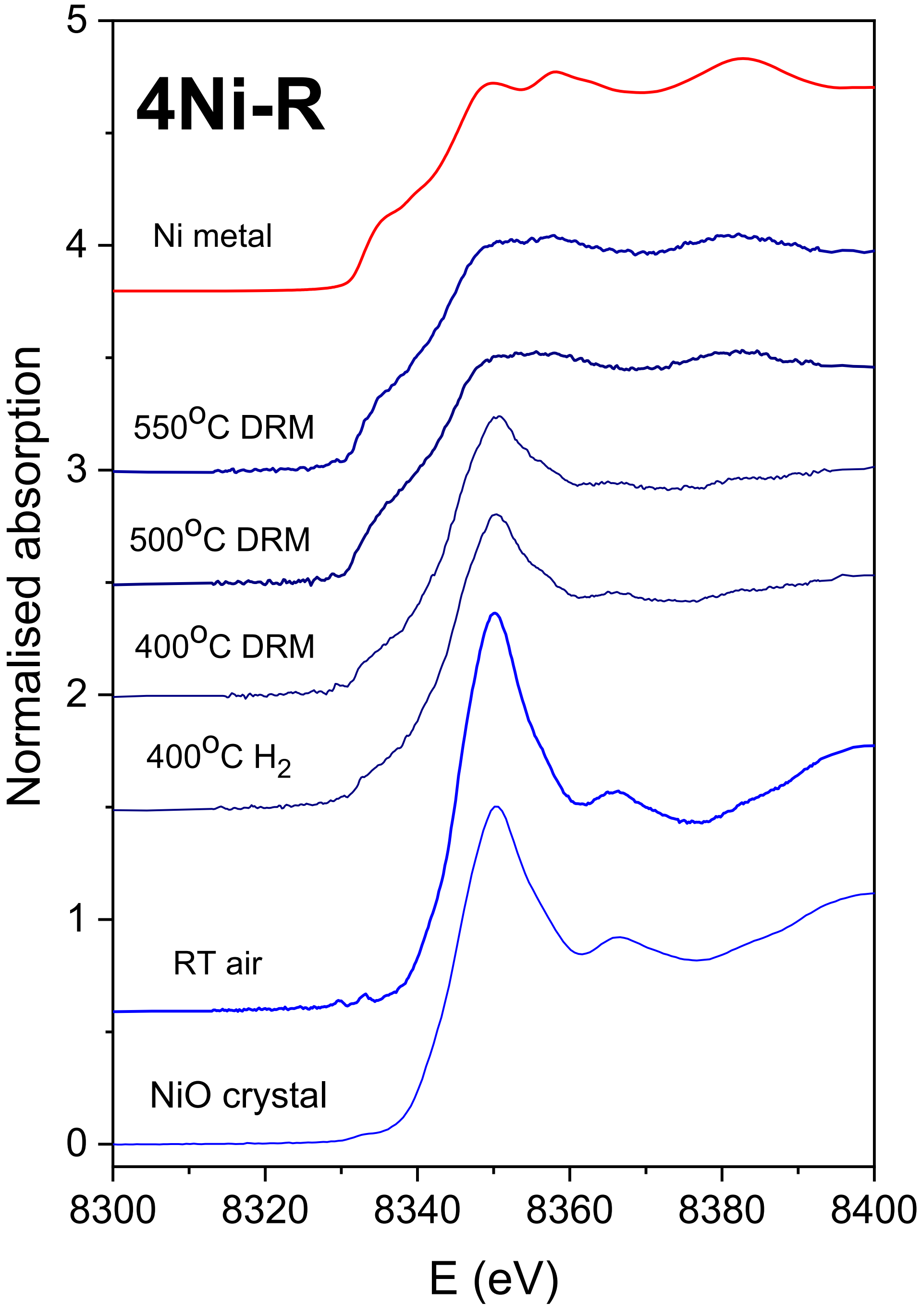

Figure S21. Normalized Ni K-edge XANES spectra measured in situ on the 4Ni-R catalyst measured in-situ at RT in air, after reduction in 5% H_2/N_2 flow for 1h at 400 °C, and during DRM reaction in equimolar CO_2/CH_4 flow at 400 °C, 500 °C and 550 °C**.** The spectrum of Ni metal and the spectrum of crystalline NiO, as a reference for Ni^{2+}, are shown for comparison.

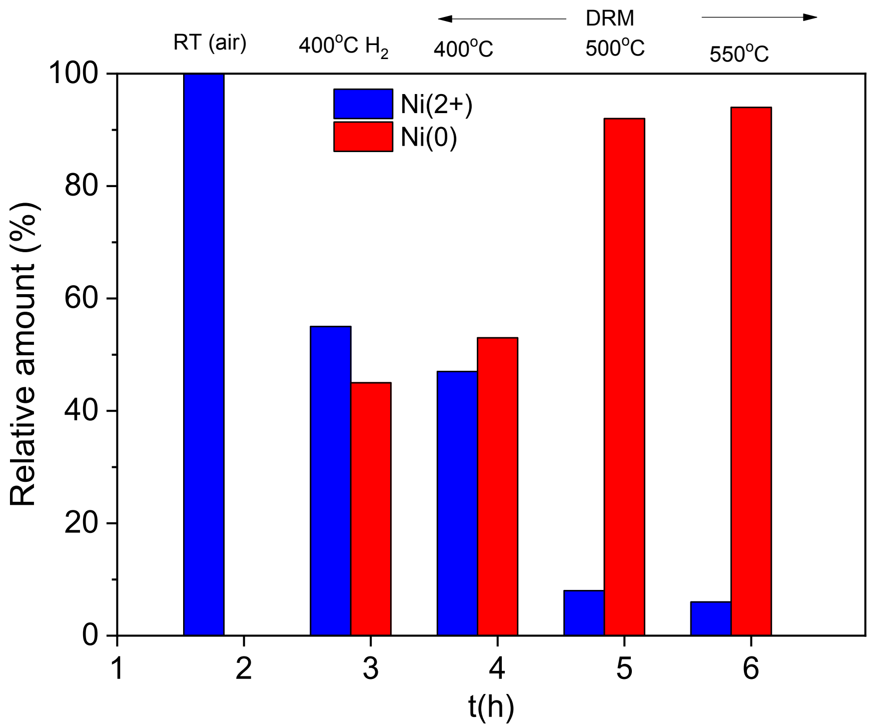

Figure S22. Relative amounts of Ni^{2+} and Ni^0 in 4Ni-R catalysts at RT in air, after reduction in 5% H_2/N_2 flow for 1h at 400 °C, and during DRM reaction in equimolar CO_2/CH_4 flow at 400 °C, 500 °C and 550 °C.

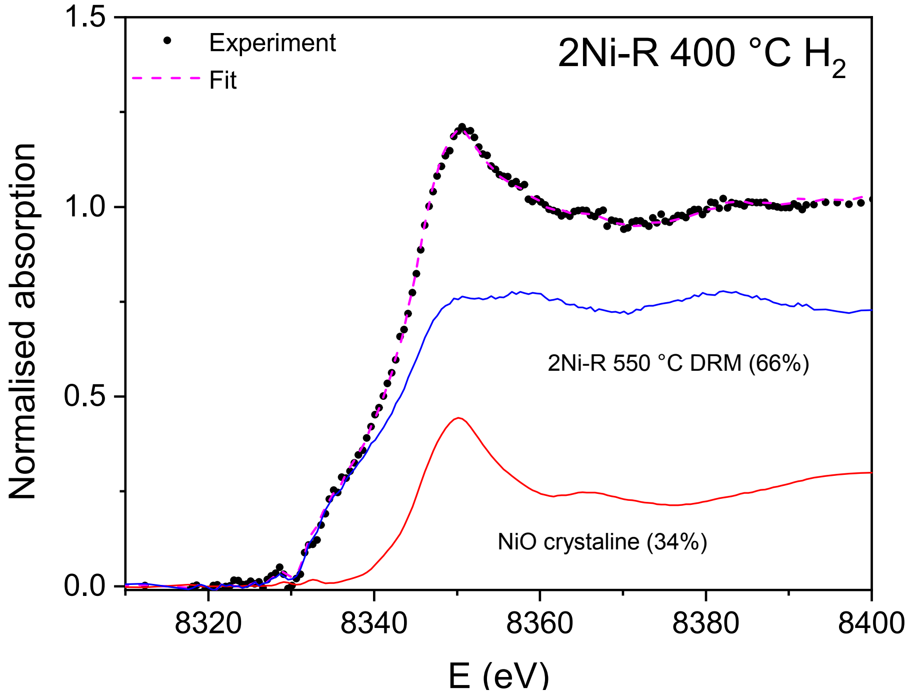

Figure S23. Ni K-edge XANES spectrum of 2Ni-R catalyst after reduction in 5% H_2/N_2 flow for 1h at 400 °C.. (Black dots: experiment; magenta dashed line: best fit with linear combination of reference XANES profiles (the spectrum of crystaline NiO as reference for Ni^{2+} and the spectrum of 2Ni-R catalyst measured after DRM reaction at 550°C as reference for Ni metal nanoparticles (with small amount, about 5%, of Ni^{2+}), plotted below.

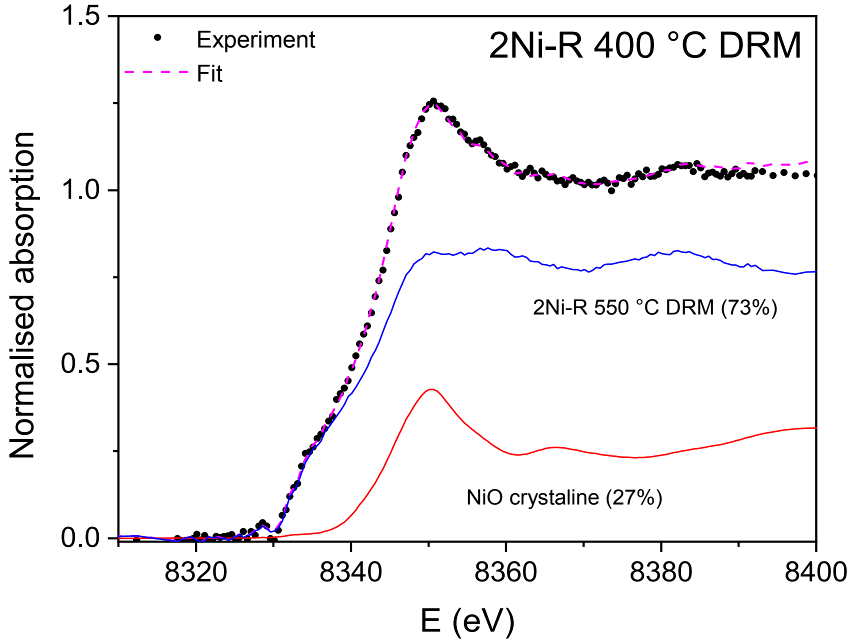

Figure S24. Ni K-edge XANES spectrum of 2Ni-R catalyst during DRM reaction in equimolar CO_2/CH_4 flow 400 °C.. (Black dots: experiment; magenta dashed line: best fit with linear combination of reference XANES profiles (the spectrum of crystaline NiO as reference for Ni^{2+} and the spectrum of 2Ni-R catalyst measured after DRM reaction at 550°C as reference for Ni metal nanoparticles (with small amount, about 5%, of Ni^{2+}), plotted below.

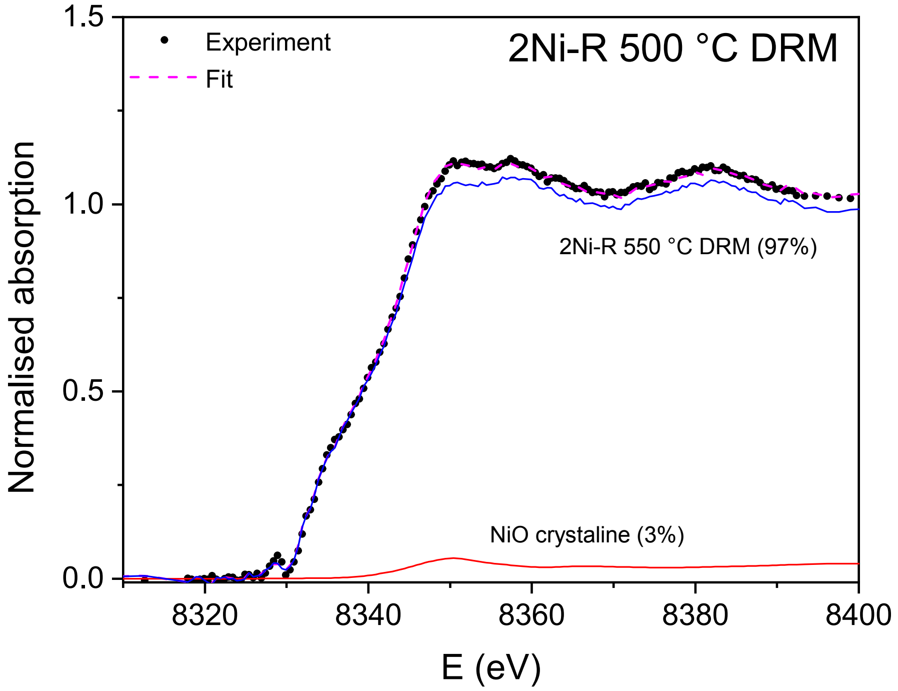

Figure S25. Ni K-edge XANES spectrum of 2Ni-R catalyst during DRM reaction in equimolar CO_2/CH_4 flow 500 °C. (Black dots: experiment; magenta dashed line: best fit with linear combination of reference XANES profiles (the spectrum of crystaline NiO as reference for Ni^{2+} and the spectrum of 2Ni-R catalyst measured after DRM reaction at 550°C as reference for Ni metal nanoparticles (with small amount, about 5%, of Ni^{2+}), plotted below.

3.0 Ni K-edge EXAFS analysis

In-situ Ni K-edge EXAFS analysis is used to monitor the changes of local structure around Ni cations in the 2Ni-R, 2Ni-C, 2Ni-S and 4Ni-R catalysts after the activation in 5% H_2/N_2 flow at 400 °C and during DRM reaction at 400°C, 500 °C and 550 °C. In Fourier transform magnitude of the EXAFS spectra (Figures S26-S29**)** the contributions of photoelectron scattering on the nearest shells of neighbors around the Ni atoms are observed in the R range up to about 4 Å. Already by qualitative comparisons of the FT EXAFS spectra, the differences in average Ni neighbourhoods that apper during the in-situ expoeriment, are evident. Details of quantitative EXAFS analysis and best fit structural parameters are presented in Figures S26-S29 and Tables S2-S8.

Structural parameters of the average local Ni neighborhood (type and average number of neighbors, the radii and Debye-Waller factor of neighbor shells) are quantitatively resolved from the EXAFS spectra by comparing the measured EXAFS signal with model signal, constructed *ab initio* with the FEFF6 program code[25]. A combined FEFF model is used, composed of neighbor atoms at distances characteristic for the expected Ni oxide and Ni metal species, indcated by XANES analysis of the catalyst at different states (at RT in air, after activation in 5%H_2/N_2 and during DRM reaction in equimolar undiluted CH_4-CO_2 atmosphere). The atomic species of neighbors are identified in the fit by their specific scattering factor and phase shift.

The FEFF model for Ni oxide is based on monoclinic crystal structure of NiO with space group *Fm3m* with the lattice constant a = 4.177 Å, where Ni is coordinated to 6 oxygen atoms at a distance of 2.08 Å, 12 Ni atoms at 2.95 Å and 8 oxygen atoms at 3.62 Å. The FEFF model comprised three single scattering and two significant multiple scattering paths up to 4 Å, with 8 variable parameters: coordination shell distance (Δr) and Debye-Waller factors (σ^2) of all single scattering paths are introduced, and the amplitude reduction factor S_0^2, shift of energy origin of the photoelectron ΔE_o, common to all scattering paths. The shell coordination numbers were fixed to the crystallographic values. The structural parameters of multiple scattering paths are constrained to to those of the corresponding single scattering paths. The model is tested on the EXAFS spectrum measured on crystalline NiO. A very good EXAFS fit (Fig. S26) obtained in the in the k range of 3 – 14 $Å^{-1}$ and the R-range of 1.2 – 3.3 Å. The best fit structural parameters are lined in the Table S2.

The FEFF model of *fcc* crystal structure of Ni metal with the lattice constant, a = 3.520 Å was composed of all four single and all multiple scattering paths up to 4.8 Å. The model was calibrated by the Ni metal foil spectrum in the k range of 2 – 15 $Å^{-1}$ with six variable parameters: the amplitude reduction factor (S_0^2), the shift of energy origin of the

photoelectron (ΔE_o), the lattice expansion ($\Delta r/r$), Debye temperature in modelling the Debye-Waller factors (σ^2) of all paths except the first, for which a separate distance correction Δr_1 and Debye-Waller factor (σ^2) were introduced. The shell coordination numbers were fixed at their *fcc* values (12, 6, 24, 12). The model precisely describes EXAFS spectrum measured on Cu metal foil (Figure S26). The best fit structural parameters are lined in the Table S3.
The Ni K-edge EXAFS spectra of the catalyst samples in the initial state at RT in air can be copmpletly described by the FEFF model for crystalline NiO, where also the relative coordination shell numbers N_{rel} were allowed to vary. Results of EXAFS analysis (Table S4, Figures S26-S29) show that in all studied catalysts at RT in air Ni cations are coordinated to oxygen atoms in the first coordination shell and Ni and O neighbors in more distant coordination shells, at distances characteristic for Ni oxide. In case of 2Ni-S catalyst an additional coordination shell compised of Ce atoms at about 3.6 Å had to be introduced in the EXAFS model.

Ni K-edge EXAFS spectra of the catalysts measured *in-situ* after activation in H_2 at 400 °C, and during DRM reaction at 400 °C, 500 °C and 550 °C, were modelled with a combined, NiO and Ni metal, FEFF model, composed of neighbour atoms at distances characteristic for the expected Ni oxide and Ni metal species that may be present in the samples as indicated by XANES analysis. The proposed FEFF model aims to describe the average Ni local neighbourhood and identify the main structural changes before and during the catalytic DRM reaction.

Three variable parameters for each shell of neighbors are introduced in the model: the relative shell coordination number (N_{rel}), the distance (R) and the Debye-Waller factor (σ^2). In addition, a common shift of energy origin ΔE_o is also allowed to vary. The amplitude-reduction factor S_0^2 is kept fixed at the value of 0.9. A good agreement between the model and the experimental spectra is found using the k range of 3 Å^{-1} to 10 Å^{-1} and the R range of 1.0 Å to 3.5 Å. Best fit results are presented in Figures S26-S29).The list of best fit parameters is given in the and Tables S2-S8.

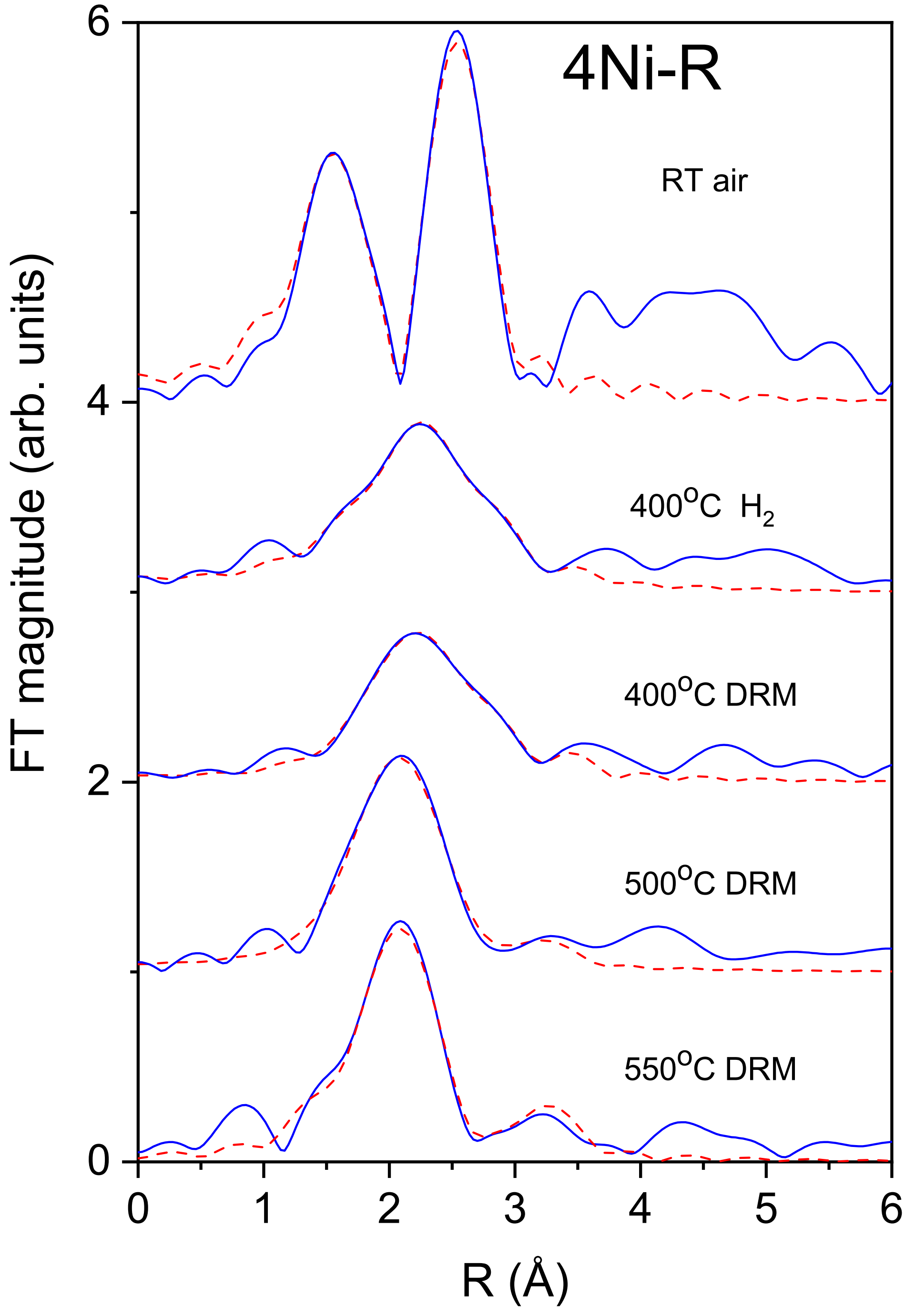

Figure S26. Fourier transform magnitude of k^2-weighted Ni K-edge EXAFS spectra of the 4Ni-R catalyst measured in-situ at RT in air, after reduction in 5% H_2/N_2 flow for 1h at 400 °C, and during DRM reaction in equimolar CO_2/CH_4 flow at 400 °C, 500 °C and 550 °C, calculated in the k range of 3–10 $Å^{-1}$. Experiment – (solid line); best fit EXAFS model calculated in the R range of 1 to 3.5 Å -- (dashed line). Spectra are shifted vertically for clarity.

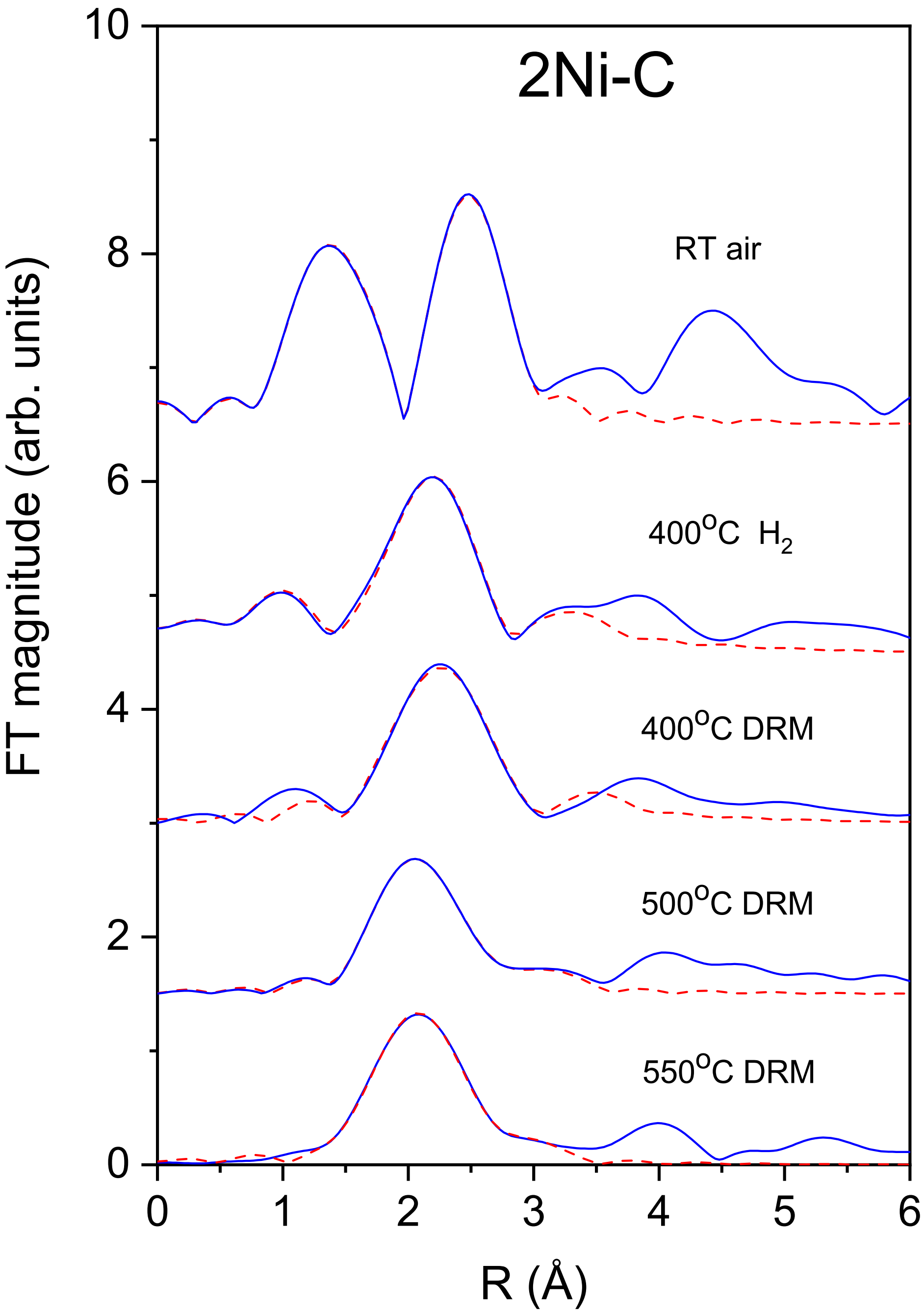

Figure S27. Fourier transform magnitude of k^2-weighted Ni K-edge EXAFS spectra of the 2Ni-C catalyst measured in-situ at RT in air, after reduction in 5% H_2/N_2 flow for 1h at 400 °C, and during DRM reaction in equimolar CO_2/CH_4 flow at 400 °C, 500 °C and 550 °C, calculated in the k range of 3–10 $Å^{-1}$. Experiment – (solid line); best fit EXAFS model calculated in the R range of 1 to 3.5 Å -- (dashed line). Spectra are shifted vertically for clarity.

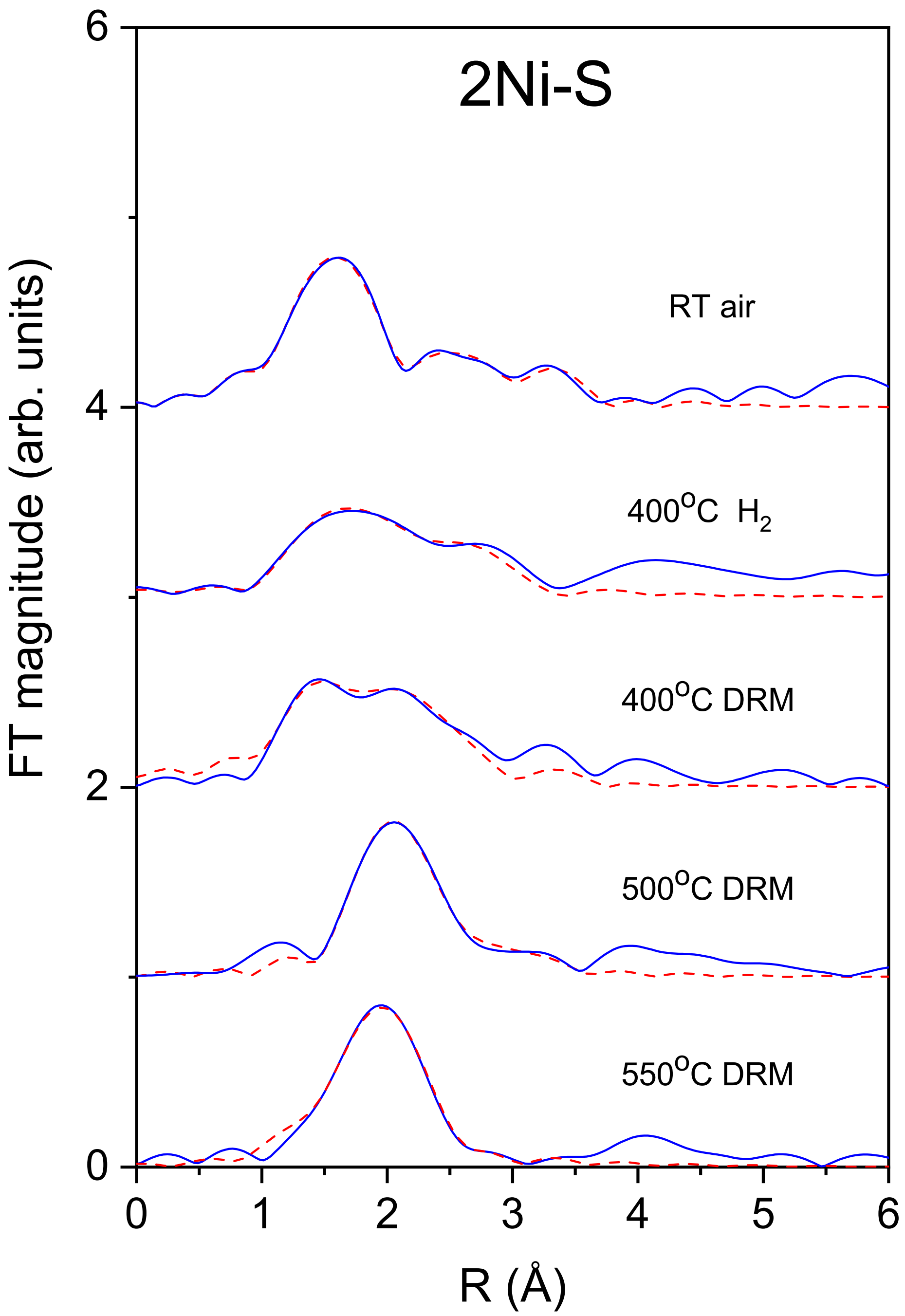

Figure S28. Fourier transform magnitude of k^2-weighted Ni K-edge EXAFS spectra of the 2Ni-S catalyst measured in-situ at RT in air, after reduction in 5% H_2/N_2 flow for 1h at 400 °C, and during DRM reaction in equimolar CO_2/CH_4 flow at 400 °C, 500 °C and 550 °C, calculated in the k range of 3–10 Å^{-1}. Experiment – (solid line); best fit EXAFS model calculated in the R range of 1 to 3.5 Å -- (dashed line). Spectra are shifted vertically for clarity.

Table S3. Parameters of the nearest coordination shells around Ni cations in **the crystalline NiO reference sample**: average number of neighbour atoms (N), distance (R), and Debye-Waller factor (σ^2). Uncertainty of the last digit is given in parentheses. A best fit is obtained with the amplitude reduction factor S_0^2=0.90(5) and the shift of the energy origin ΔE_o -2 eV. The R-factor (quality of fit parameter) is listed in the last column.

Ni neigh.	*N*	*R* [Å]	σ^2 [Å^2]	*R*-factor
**NiO crystaline**				
O	6	2.080(5)	0.0047(5)	
Ni	12	2.952(2)	0.0053(5)	0.0016
O	8	3.42(3)	0.009(2)	

Table S4. Parameters of the nearest coordination shells around Ni cations in **the crystalline Ni metal reference sample**: average number of neighbour atoms (N), distance (R), and Debye-Waller factor (σ^2). Uncertainty of the last digit is given in parentheses. A best fit is obtained with the amplitude reduction factor S_0^2=0.90(5) and the shift of the energy origin ΔE_o = -6 eV. The R-factor (quality of fit parameter) is listed in the last column.

Ni neigh.	*N*	*R* [Å]	σ^2 [Å^2]	*R*-factor
**Ni metal (fcc)**				
Ni	12	2.483(2)	0.0067(3)	
Ni	6	3.521(2)	0.009(1)	0.0083
Ni	24	4.313(3)	0.009(1)	
Ni	12	4.980(3)	0.009(1)	

3.1 The Ni-CeO_2 catalysts with different ceria morphologies analyzed in air at room temperature

Table S5. Parameters of the nearest coordination shells around Ni cations in the **fresh 2%Ni and 4%Ni CeO_2 catalyst with different morphologies (rods, cubes and spheres) analyzed in air at RT**: average number of neighbour atoms presented as a product of full occupancy in bulk (N) multiplied by relative coordination number N_{rel}, distance (R), and Debye-Waller factor (σ^2). Uncertainty of the last digit is given in parentheses. A best fit is obtained with the amplitude reduction factor S_0^2=0.90 and the shift of the energy origin ΔE_o = 2 eV. The R-factor (quality of fit parameter), is listed in the last column.

Ni neigh.	$N*Ni_{rel}$	*R* [Å]	σ^2 [Å^2]	*R*-factor
**2Ni-R**				
O	6*1.0(1)	2.07(1)	0.009(1)	0.0074
Ni	12*0.5(1)	2.97(1)	0.007(1)	
O	8*0.5(1)	3.45(3)	0.009(2)	

Ni neigh.	$N*Ni_{rel}$	*R* [Å]	σ^2 [Å^2]	*R*-factor
**4Ni-R**				
O	6*0.9(1)	2.08(1)	0.007(1)	0.0048
Ni	12*0.5(1)	2.97(1)	0.006(1)	
O	8*0.5(1)	3.48(3)	0.009(2)	

Ni neigh.	$N*Ni_{rel}$	*R* [Å]	σ^2 [Å^2]	*R*-factor
**2Ni-C**				
O	6*1.0(1)	2.02(4)	0.006(1)	0.0028
Ni	12*1.0(1)	2.93(4)	0.012(1)	
O	8*1.0(1)	3.31(5)	0.009(2)	

Ni neigh.	$N*Ni_{rel}$	*R* [Å]	σ^2 [Å^2]	*R*-factor
**2Ni-S**				
O	6*0.8(2)	2.07(2)	0.013(4)	0.0039
Ni	12*0.3(1)	3.05(6)	0.021(9)	
O	8*0.6(1)	3.35(6)	0.012(4)	
Ce	1.2(4)	3.59(3)	0.007(2)	

3.2. The 2Ni-R catalyst analyzed *in-situ* during activation and under reaction conditions

Table S6. Parameters of the nearest coordination shells around Ni cations in the **2Ni-R catalyst analyzed *in-situ* after reduction in 5% H_2/N_2 stream for 1h at 400°C, and during DRM reaction, under reaction conditions at 400°C, 500°C and 550°C**: average number of neighbour atoms presented as a product of full occupancy in bulk (N) multiplied by relative coordination number N_{rel}, distance (*R*), and Debye-Waller factor (σ^2). A combination of two FEFF models is used: NiO and Cu metal. Uncertainty of the last digit is given in parentheses. A best fit is obtained with the amplitude reduction factor S_0^2=0.90 and the shift of the energy origin ΔE_o = 2 eV. *R*-factor (quality of fit parameter) is listed in the last column.

Ni neigh.	$N*Ni_{rel}$	*R* [Å]	σ^2 [Å^2]	*R*-factor
**2Ni-R in H_2 at 400 °C**				
Ni oxide species				
O	6*0.4(2)	2.00(5)	0.015(2)	0.0037
Ni	12*0.4(2)	3.41(3)	0.011(2)	
Metallic Ni				
Ni	12*0.6(2)	2.47(4)	0.017(3)	
Ni	12*0.6(2)	3.64(4)	0.017(3)	

Ni neigh.	$N*Ni_{rel}$	*R* [Å]	σ^2 [Å^2]	*R*-factor
**2Ni-R in CH_4_CO_2 at 400 °C**				
Ni oxide species				
O	6*0.5(1)	2.01(5)	0.006(2)	0.0035
Ni	12*0.5(1)	3.41(3)	0.007(2)	
Metallic Ni				
Ni	12*0.5(1)	2.48(4)	0.012(3)	
Ni	12*0.5(1)	3.63(4)	0.012(3)	

Ni neigh.	$N*Ni_{rel}$	*R* [Å]	σ^2 [Å^2]	*R*-factor
**2Ni-R in CH_4_CO_2 at 500 °C**				
Ni oxide species				
O	6*0.11(4)	2.07(5)	0.006(2)	0.0089
Ni	12*0.11(4)	3.25(3)	0.007(2)	
Metallic Ni				
Ni	12*0.88(5)	2.45(4)	0.018(3)	
Ni	12*0.88(5)	3.48(4)	0.018(3)	

Ni neigh.	$N*Ni_{rel}$	*R* [Å]	σ^2 [Å^2]	*R*-factor
**2Ni-R in CH_4_CO_2 at 550 °C**				
Ni oxide species				
O	6*0.11(4)	1.94(2)	0.008(2)	0.0062
Ni	12*0.11(4)	3.10(3)	0.012(2)	
Metallic Ni				
Ni	12*0.89(5)	2.47(4)	0.018(3)	
Ni	12*0.89(5)	3.40(4)	0.018(3)	

3.3 The 2Ni-C catalyst analyzed *in-situ* during activation and under reaction conditions

Table S7. Parameters of the nearest coordination shells around Ni cations in the **2Ni-C catalyst analyzed *in-situ* after reduction in 5% H_2/N_2 stream for 1h at 400°C, and during DRM reaction, under reaction conditions at 400°C, 500°C and 550°C**: average number of neighbour atoms presented as a product of full occupancy in bulk (N) multiplied by relative coordination number N_{rel}, distance (R), and Debye-Waller factor (σ^2). A combination of two FEFF models is used: NiO and Cu metal. Uncertainty of the last digit is given in parentheses. A best fit is obtained with the amplitude reduction factor S_0^2=0.90 and the shift of the energy origin ΔE_o = 2 eV. R-factor (quality of fit parameter) is listed in the last column.

Ni neigh.	$N*Ni_{rel}$	R [Å]	σ^2 [Å^2]	R-factor
**2Ni-C in H_2 at 400 °C**				
Ni oxide species				
O	6*0.2(1)	2.06(5)	0.008(2)	0.0036
Ni	12*0.2(1)	3.02(7)	0.012(3)	
Metallic Ni				
Ni	12* 0.3(1)	2.50(5)	0.005(2)	
Ni	6*0.3(1)	3.53(5)	0.005(2)	

Ni neigh.	$N*Ni_{rel}$	R [Å]	σ^2 [Å^2]	R-factor
**2Ni-C in CH_4_CO_2 at 400 °C**				
Ni oxide species				
O	6*0.09(3)	2.06(5)	0.008(2)	0.0047
Ni	12*0.09(3)	3.02(7)	0.012(3)	
Metallic Ni				
Ni	12* 0.33(2)	2.50(5)	0.005(2)	
Ni	6*0.33(2)	3.53(5)	0.005(2)	

Ni neigh.	$N*Ni_{rel}$	R [Å]	σ^2 [Å^2]	R-factor
**2Ni-C in CH_4_CO_2 at 500 °C**				
Ni oxide species				
O	6*0.11(6)	2.11(9)	0.009(2)	0.0008
Ni	12*0.11(6)	2.98(7)	0.012(3)	
Metallic Ni				
Ni	12* 0.7(2)	2.45(5)	0.015(2)	
Ni	6*0.7(2)	3.40(5)	0.015(2)	

Ni neigh.	$N*Ni_{rel}$	R [Å]	σ^2 [Å^2]	R-factor
**2%Ni-C in CH_4_CO_2 at 550 °C**				
Ni oxide species				
O	6*0.2(1)	2.00(9)	0.009(2)	0.0006
Ni	12*0.2(1)	2.92(7)	0.012(3)	
Metallic Ni				
Ni	12* 0.5(2)	2.41(5)	0.010(2)	
Ni	6*0.5(2)	3.39(5)	0.010(2)	

3.4 The 2Ni-S catalyst analyzed *in-situ* during activation and under reaction conditions

Table S8. Parameters of the nearest coordination shells around Ni cations in the **2Ni-S catalyst analyzed *in-situ* after reduction in 5% H_2/N_2 stream for 1h at 400°C, and during DRM reaction, under controlled reaction conditions at 400°C, 500°C and 550°C**: average number of neighbour atoms presented as a product of full occupancy in bulk (N) multiplied by relative coordination number N_{rel}, distance (*R*), and Debye-Waller factor (σ^2). A combination of two FEFF models is used: NiO and Cu metal. Uncertainty of the last digit is given in parentheses. A best fit is obtained with the amplitude reduction factor S_0^2=0.90 and the shift of the energy origin ΔE_o = 2 eV. *R*-factor (quality of fit parameter) is listed in the last column.

Ni neigh.	$N*Ni_{rel}$	*R* [Å]	σ^2 [Å^2]	*R*-factor
**2Ni-S in H_2 at 400 °C**				
Ni oxide species				
O	6*0.19(6)	1.90(5)	0.004(2)	0.05
Ni	12*0.19(6)	2.94(7)	0.012(3)	
Metallic Ni				
Ni	12* 0.81(6)	2.58(4)	0.04(1)	
Ni	6*0.81(6)	3.54(2)	0.04(1)	

Ni neigh.	$N*Ni_{rel}$	*R* [Å]	σ^2 [Å^2]	*R*-factor
**2%Ni CeO_2_sphere catalyst in CH_4_CO_2 at 400 °C**				
Ni oxide species				
O	6*0.19(6)	1.85(5)	0.004(1)	0.041
Ni	12*0.19(6)	2.84(4)	0.012(4)	
Metallic Ni				
Ni	12*0.81(6)	2.53(4)	0.027(3)	
Ni	12*0.81(6)	3.52(4)	0.027(3)	

Ni neigh.	$N*Ni_{rel}$	*R* [Å]	σ^2 [Å^2]	*R*-factor
**2%Ni CeO_2_sphere catalyst in CH4_CO2 at 500 °C**				
Ni oxide species				
O	6*0.07(4)	2.09(5)	0.003(1)	0.0077
Ni	12*0.07(4)	2.82(4)	0.017(4)	
Metallic Ni				
Ni	12*0.4(1)	2.42(4)	0.014(3)	
Ni	12*0.4(1)	3.37(4)	0.014(3)	

Ni neigh.	$N*Ni_{rel}$	*R* [Å]	σ^2 [Å^2]	*R*-factor
**2Ni-CeO_2_sphere catalyst in CH_4_CO_2 at 550 °C**				
Ni oxide species				
O	6*0.08(4)	1.89(5)	0.003(1)	0.0037
Ni	12*0.08(4)	2.68(4)	0.010(4)	
Metallic Ni				
Ni	12*0.3(1)	2.48(4)	0.010(3)	

3.5 The 4Ni-R catalyst analyzed *in-situ* during activation and under reaction conditions

Table S9. Parameters of the nearest coordination shells around Ni cations in the **4Ni-R catalyst analyzed *in-situ* after reduction in 5% H_2/N_2 stream for 1h at 400°C, and during DRM reaction, under controlled reaction conditions at 400°C, 500°C and 550°C**: average number of neighbour atoms presented as a product of full occupancy in bulk (N) multiplied by relative coordination number N_{rel}, distance (*R*), and Debye-Waller factor (σ^2). A combination of two FEFF models is used: NiO and Cu metal. Uncertainty of the last digit is given in parentheses. A best fit is obtained with the amplitude reduction factor S_0^2=0.90 and the shift of the energy origin ΔE_o = 2 eV. *R*-factor (quality of fit parameter) is listed in the last column.

Ni neigh.	$N*Ni_{rel}$	*R* [Å]	σ^2 [Å^2]	*R*-factor
**4Ni-R in H_2 at 400 °C**				
Ni oxide species				
O	6*0.43(4)	2.11(2)	0.008(2)	
Ni	12*0.43(4)	3.12(3)	0.019(4)	0.0017
Metallic Ni				
Ni	12*0.6(1)	2.54(2)	0.016(2)	
Ni	12*0.6(1)	3.50(2)	0.016(2)	

Ni neigh.	$N*Ni_{rel}$	*R* [Å]	σ^2 [Å^2]	*R*-factor
**4Ni-R in CH_4_CO_2 at 400 °C**				
Ni oxide species				
O	6*0.24(4)	2.09(2)	0.008(2)	
Ni	12*0.24(4)	3.14(3)	0.012(4)	0.002
Metallic Ni				
Ni	12*0.5(1)	2.56(2)	0.015(3)	
Ni	12*0.5(1)	3.48(2)	0.015(3)	

Ni neigh.	$N*Ni_{rel}$	*R* [Å]	σ^2 [Å^2]	*R*-factor
**4Ni-R in CH_4_CO_2 at 500 °C**				
Ni oxide species				
O	6*0.10(4)	1.94(2)	0.008(2)	
Ni	12*0.10(4)	2.93(3)	0.013(4)	0.018
Metallic Ni				
Ni	12*0.9(1)	2.46(2)	0.020(3)	
Ni	12*0.9(1)	3.47(2)	0.020(3)	

Ni neigh.	$N*Ni_{rel}$	*R* [Å]	σ^2 [Å^2]	*R*-factor
**4Ni-R in CH_4_CO_2 at 550 °C**				
Ni oxide species				
O	6*0.10(4)	1.91(2)	0.008(2)	
Ni	12*0.10(4)	2.75(3)	0.006(2)	0.0074
Metallic Ni				
Ni	12*0.9(1)	2.51(2)	0.017(3)	
Ni	12*0.9(1)	3.50(2)	0.017(3)	

4. DFT Calculations

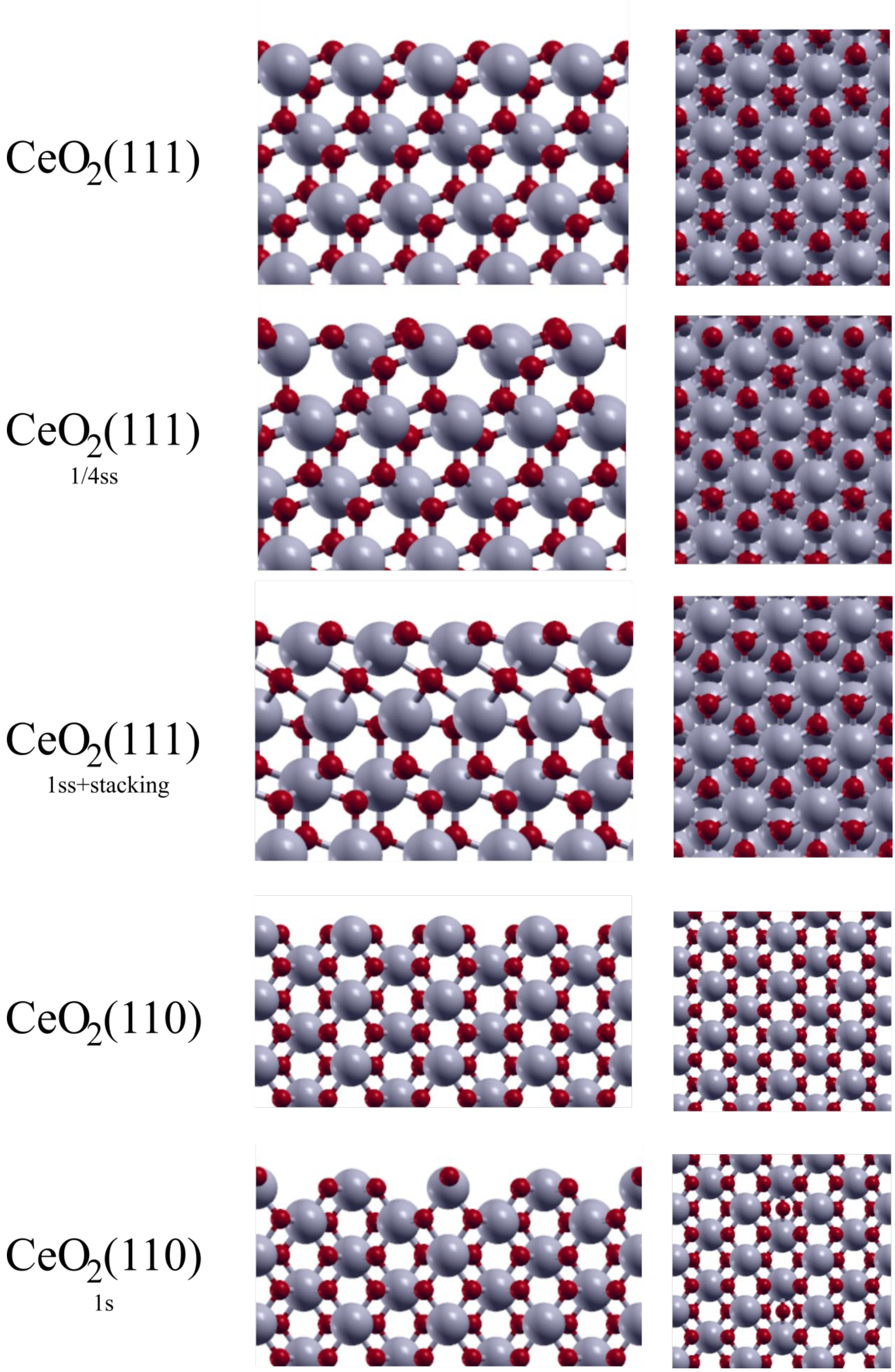

Figure S29. Slabs used to model various terminations of the (111) and (110) CeO_2 surfaces.

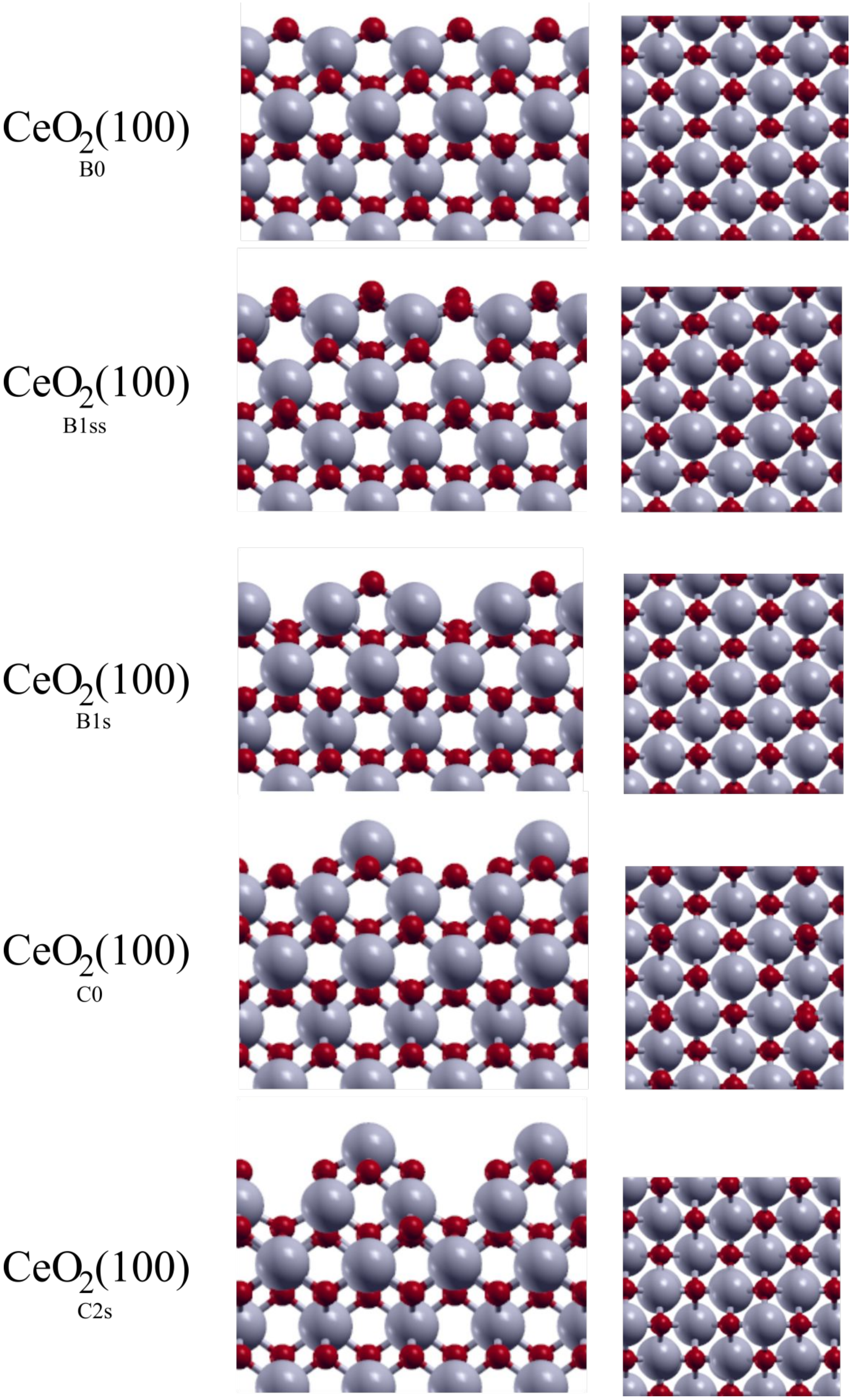

Figure S30. Slabs used to model various terminations of the (100) CeO_2 surfaces.

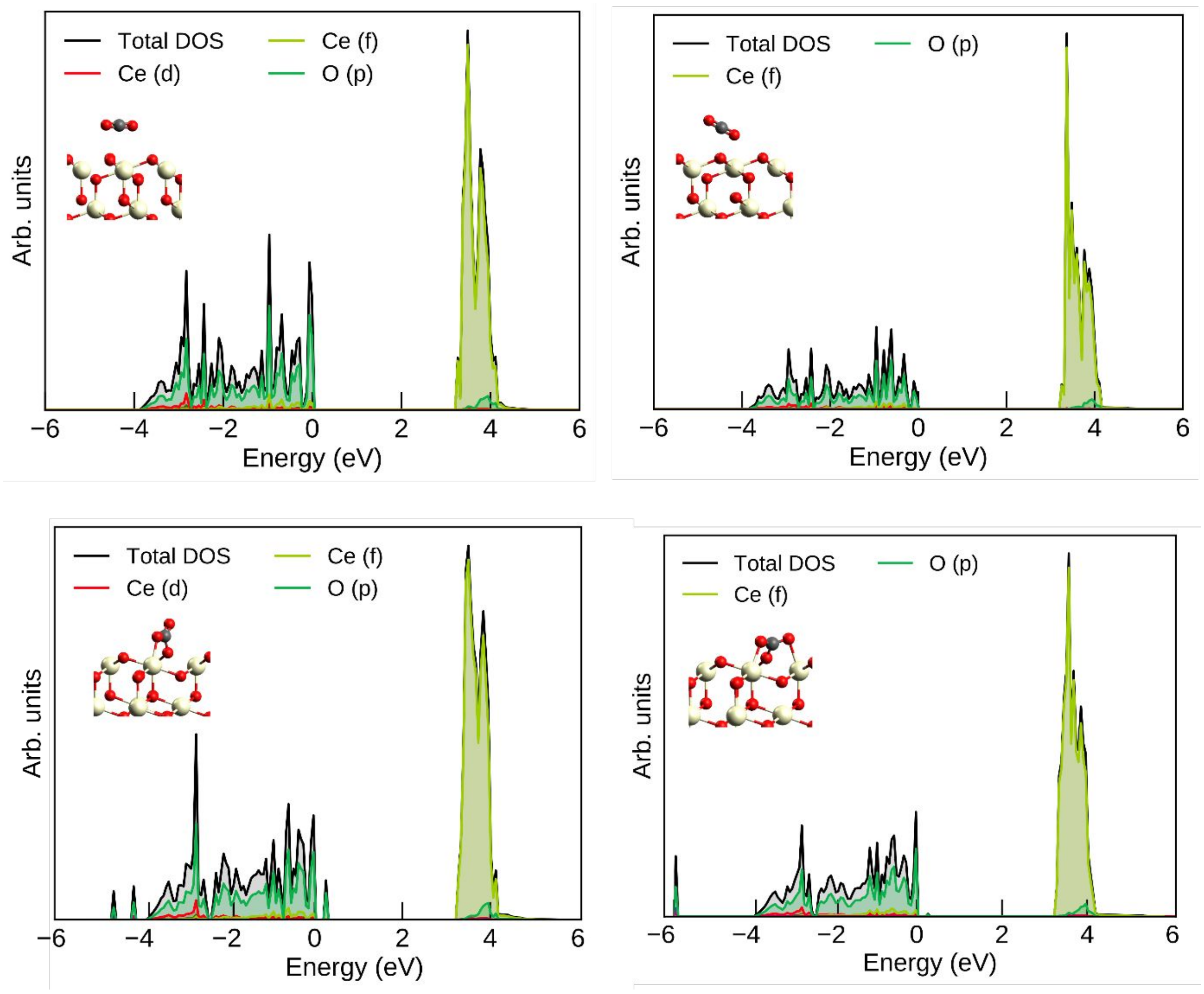

Figure S31. Projected density of states (PDOS) of electronic density for different adsorption modes of CO_2 on CeO_2(111).

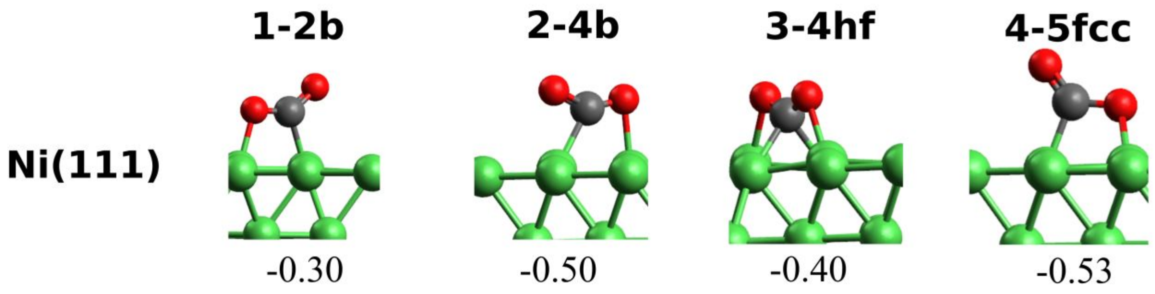

Figure S32. Adsorption modes of CO_2 on Ni(111). For consistency, the adsorption modes on Ni(111) are named as in.[26] The values represent adsorption energies in eV.

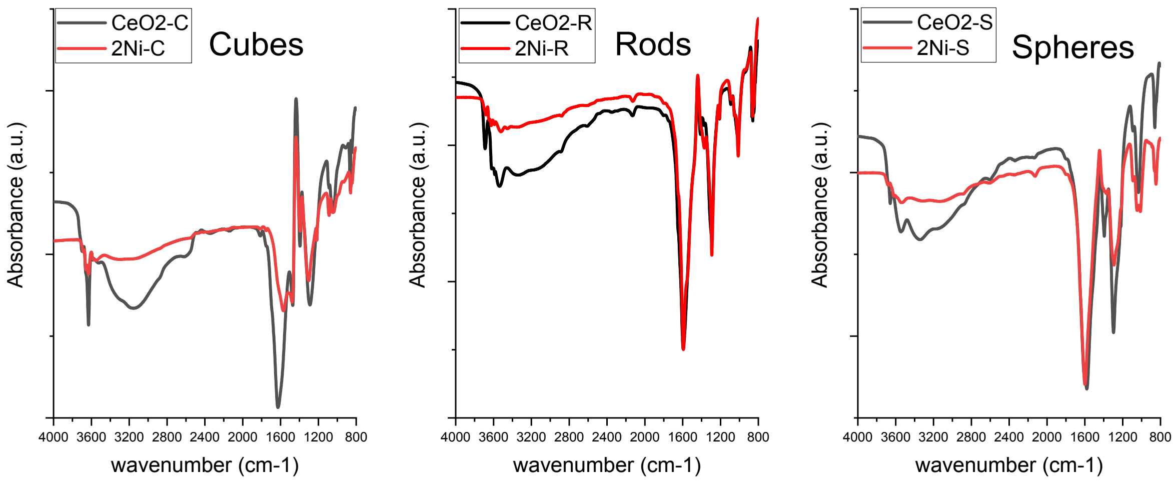

Figure S33. Difference DRIFT spectra (RT - 500°C) for ceria cube, rod and sphere morphologies with and without nickel.

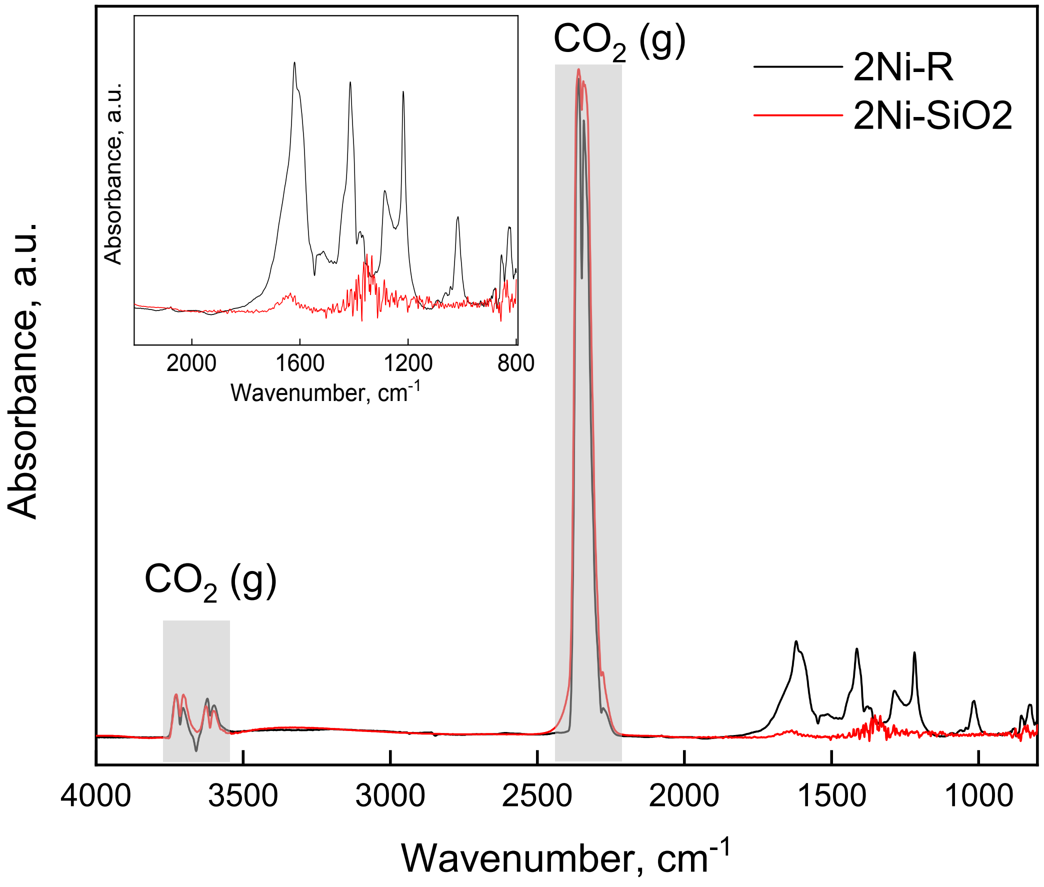

Figure S34. DRIFT spectra of 2Ni/CeO_2-R and 2Ni/SiO_2 catalysts, recorded after reduction in 5% H_2 at 500 °C for 30 min, cooling in argon and CO_2 adsorption at 25 °C.

Table S10. Adsorption modes, energies, charge transfer, bond lengths and angles for CO_2 adsorption on different ceria surfaces and Ni (111) facet.

Surface	Adsorption mode	Adsorption energy (eV)	Charge transfer *to* CO_2 (e^-)	Angle O-C-O (°)	Bond lengths (O-C) (Å)
$CeO_2(111)$	null	–0.15	0.00	179°	1.18
$CeO_2(111)$	mono	–0.24	0.00	179°	1.18
$CeO_2(111)$	bi	–0.38	–0.15	131°	1.29<br>1.22
$CeO_2(111)$	tri	–0.68	–0.17	130°	1.27
$CeO_2(111)_{(1/4ss)}$	null	–0.15	0.00	179°	1.18
$CeO_2(111)_{(1/4ss)}$	mono	–0.24	–0.01	179°	1.18
$CeO_2(111)_{(1/4ss)}$	bi	–0.28	–0.21	133°	1.28<br>1.23
$CeO_2(111)_{(1/4ss)}$	tri	–0.71	–0.22	130°	1.26
$CeO_2(111)_{(1ss+stacking)}$	null	–0.15	0.00	179°	1.18
$CeO_2(111)_{(1ss+stacking)}$	mono	–0.24	–0.01	179°	1.18
$CeO_2(111)_{(1ss+stacking)}$	bi	–0.33	–0.27	134°	1.28<br>1.22
$CeO_2(111)_{(1ss+stacking)}$	tri	–0.70	–0.27	131°	1.26
$CeO_2(110)$	null	–0.03	0.00	179°	1.18
$CeO_2(110)$	mono	–0.22	0.00	180°	1.17
$CeO_2(110)$	bi	–0.46	–0.01	177°	1.17
$CeO_2(110)$	tri	–1.53	–0.21	127°	1.28
$CeO_2(110)_{(1s)}$	null	–0.03	0.00	179°	1.18
$CeO_2(110)_{(1s)}$	mono	–0.14	0.00	180°	1.18
$CeO_2(110)_{(1s)}$	bi	–0.42	–0.03	176°	1.18
$CeO_2(110)_{(1s)}$	tri	–1.16	–0.26	129°	1.27
$CeO_2(100)$:B0	null	–0.07	0.00	180°	1.18
$CeO_2(100)$:B0	mono	–0.49	–0.03	174°	1.19<br>1.16
$CeO_2(100)$:B0	bi	–0.38	–0.07	131°	1.22<br>1.31
$CeO_2(100)$:B0	carbonate	–2.45	–0.33	124°	1.30
$CeO_2(100)$:$B1_{ss}$	null	–0.07	0.00	180°	1.18
$CeO_2(100)$:$B1_{ss}$	mono	–0.49	–0.03	174°	1.19<br>1.16
$CeO_2(100)$:$B1_{ss}$	bi	–0.09	–0.14	135°	1.23<br>1.27
$CeO_2(100)$:$B1_{ss}$	carbonate	–1.66	–0.27	127°	1.28
$CeO_2(100)$:$B1_s$	null	–0.07	0.00	180°	1.18
$CeO_2(100)$:$B1_s$	mono	–0.60	–0.06	171°	1.20<br>1.17
$CeO_2(100)$:$B1_s$	bi	–0.91	–0.22	129°	1.21<br>1.33
$CeO_2(100)$:$B1_s$	carbonate	–3.28	–0.33	122°	1.30
$CeO_2(100)$:$B1_s$	bent-hollow	–0.57	–1.14	115°	1.30

CeO_2(100):$B1_s$	bent-bridge	–0.87	–1.09	124°	1.28
CeO_2(100):C0	null	–0.02	0.00	180°	1.18
CeO_2(100):C0	mono-top	–0.21	0.00	180°	1.18
CeO_2(100):C0	mono-bridge	–0.16	–0.02	179°	1.17
CeO_2(100):C0	lateral	–0.53	–0.01	176°	1.17
CeO_2(100):C0	bi	–1.09	–0.29	122°	1.30
CeO_2(100):C0	carbonate	–1.09	–0.18	128°	1.31<br>1.24
CeO_2(100):$C2_s$	null	–0.02	0.00	180°	1.18
CeO_2(100):$C2_s$	mono-top	–0.21	–0.05	180°	1.18
CeO_2(100):$C2_s$	mono-bridge	–0.51	–0.08	175°	1.17
CeO_2(100):$C2_s$	lateral	–0.36	–0.05	178°	1.18
CeO_2(100):$C2_s$	carbonate	–0.91	–0.33	132°	1.28
CeO_2(100):$C2_s$	lattice	–1.96	–1.33	122°	1.29
Ni(111)	1-2b	–0.30	–0.46	136°	1.28<br>1.21
Ni(111)	2-4b	–0.50		142°	1.24<br>1.22
Ni(111)	3-4hf	–0.40		132°	1.27
Ni(111)	4-5fcc	–0.53		130°	1.34<br>1.21

References

(1) Jiang, F.; Wang, S.; Liu, B.; Liu, J.; Wang, L.; Xiao, Y.; Xu, Y.; Liu, X. Insights into the Influence of CeO2 Crystal Facet on CO2 Hydrogenation to Methanol over Pd/CeO2 Catalysts. *ACS Catal.* **2020**.

(2) Levenspiel, O. *Chemical Reaction Engineering*, third edit.; Wiley and Sons, 1999.

(3) Dębek, R.; Motak, M.; Duraczyska, D.; Launay, F.; Galvez, M. E.; Grzybek, T.; Da Costa, P. Methane Dry Reforming over Hydrotalcite-Derived Ni–Mg–Al Mixed Oxides: The Influence of Ni Content on Catalytic Activity, Selectivity and Stability. *Catal. Sci. Technol.* **2016**, *6* (17), 6705–6715.

(4) Aghamohammadi, S.; Haghighi, M.; Maleki, M.; Rahemi, N. Sequential Impregnation vs. Sol-Gel Synthesized Ni/Al2O3-CeO2 Nanocatalyst for Dry Reforming of Methane: Effect of Synthesis Method and Support Promotion. *Mol. Catal.* **2017**, *431*, 39–48.

(5) Baudouin, D.; Rodemerck, U.; Krumeich, F.; Mallmann, A. de; Szeto, K. C.; Ménard, H.; Veyre, L.; Candy, J.-P.; Webb, P. B.; Thieuleux, C.; et al. Particle Size Effect in the Low Temperature Reforming of Methane by Carbon Dioxide on Silica-Supported Ni Nanoparticles. *J. Catal.* **2013**, *297*, 27–34.

(6) Elsayed, N. H.; Roberts, N. R. M.; Joseph, B.; Kuhn, J. N. Low Temperature Dry Reforming of Methane over Pt–Ni–Mg/Ceria–Zirconia Catalysts. *Appl. Catal. B Environ.* **2015**, *179*, 213–219.

(7) Wang, S.; G. Q. (Max) Lu. A Comprehensive Study on Carbon Dioxide Reforming of Methane over Ni/γ-Al2O3 Catalysts. *Ind. Eng. Chem. Res.* **1999**, *38* (7), 2615–2625.
(8) Yao, L.; Wang, Y.; Shi, J.; Xu, H.; Shen, W.; Hu, C. The Influence of Reduction Temperature on the Performance of ZrOx/Ni-MnOx/SiO2 Catalyst for Low-Temperature CO2 Reforming of Methane. *Catal. Today* **2017**, *281*, 259–267.
(9) Yao, L.; Shi, J.; Xu, H.; Shen, W.; Hu, C. Low-Temperature CO2 Reforming of Methane on Zr-Promoted Ni/SiO2 Catalyst. *Fuel Process. Technol.* **2016**, *144*, 1–7.
(10) Dębek, R.; Motak, M.; Galvez, M. E.; Grzybek, T.; Da Costa, P. Influence of Ce/Zr Molar Ratio on Catalytic Performance of Hydrotalcite-Derived Catalysts at Low Temperature CO2 Methane Reforming. *Int. J. Hydrogen Energy* **2017**, *42* (37), 23556–23567.
(11) Liu, H.; Wierzbicki, D.; Debek, R.; Motak, M.; Grzybek, T.; Da Costa, P.; Gálvez, M. E. La-Promoted Ni-Hydrotalcite-Derived Catalysts for Dry Reforming of Methane at Low Temperatures. *Fuel* **2016**, *182*, 8–16.
(12) Dębek, R.; Motak, M.; Galvez, M. E.; Da Costa, P.; Grzybek, T. Catalytic Activity of Hydrotalcite-Derived Catalysts in the Dry Reforming of Methane: On the Effect of Ce Promotion and Feed Gas Composition. *React. Kinet. Mech. Catal.* **2017**, *121* (1), 185–208.
(13) Wang, Y.; Yao, L.; Wang, Y.; Wang, S.; Zhao, Q.; Mao, D.; Hu, C. Low-Temperature Catalytic CO2 Dry Reforming of Methane on Ni-Si/ZrO2 Catalyst. *ACS Catal.* **2018**, *8* (7), 6495–6506.
(14) Dębek, R.; Radlik, M.; Motak, M.; Galvez, M. E.; Turek, W.; Da Costa, P.; Grzybek, T. Ni-Containing Ce-Promoted Hydrotalcite Derived Materials as Catalysts for Methane Reforming with Carbon Dioxide at Low Temperature – On the Effect of Basicity. *Catal. Today* **2015**, *257*, 59–65.
(15) Dębek, R.; Zubek, K.; Motak, M.; Galvez, M. E.; Da Costa, P.; Grzybek, T. Ni–Al Hydrotalcite-like Material as the Catalyst Precursors for the Dry Reforming of Methane at Low Temperature. *Comptes Rendus Chim.* **2015**, *18* (11), 1205–1210.
(16) Dębek, R.; Motak, M.; Galvez-Parruca, M. E.; Grzybek, T.; Da Costa, P.; Pieńkowski, L. Ceria Promotion over Ni-Containing Hydrotalcite-Derived Catalysts for CO2 Methane Reforming. *E3S Web Conf.* **2017**, *14*, 02039.
(17) Bachiller-Baeza, B.; Mateos-Pedrero, C.; Soria, M. A.; Guerrero-Ruiz, A.; Rodemerck, U.; Rodríguez-Ramos, I. Transient Studies of Low-Temperature Dry Reforming of Methane over Ni-CaO/ZrO2-La2O3. *Appl. Catal. B Environ.* **2013**, *129*, 450–459.
(18) Zhao, X.; Cao, Y.; Li, H.; Zhang, J.; Shi, L.; Zhang, D. Sc Promoted and Aerogel Confined Ni Catalysts for Coking-Resistant Dry Reforming of Methane. *RSC Adv.* **2017**, *7* (8), 4735–4745.

(19) Bradford, M. C. J.; Vannice, M. A. Catalytic Reforming of Methane with Carbon Dioxide over Nickel Catalysts I. Catalyst Characterization and Activity. *Appl. Catal. A Gen.* **1996**, *142* (1), 73–96.

(20) CO2 electron ionization fragmentation https://webbook.nist.gov/cgi/cbook.cgi?ID=C124389&Mask=200#Mass-Spec (accessed Jun 30, 2020).

(21) Smith, B. *Infrared Spectral Interpretation A Systematic Approach*; CRC Press, 1999.

(22) Lin, L.; Yao, S.; Liu, Z.; Zhang, F.; Li, N.; Vovchok, D.; Martínez-Arias, A.; Castañeda, R.; Lin, J.; Senanayake, S. D.; et al. In Situ Characterization of Cu/CeO2 Nanocatalysts for CO2 Hydrogenation: Morphological Effects of Nanostructured Ceria on the Catalytic Activity. *J. Phys. Chem. C* **2018**, *122* (24), 12934–12943.

(23) Jacobs, G.; Keogh, R.; Davis, B. H. Steam Reforming of Ethanol over Pt/Ceria with Co-Fed Hydrogen. *J. Catal.* **2007**, *245* (2), 326–337.

(24) Wu, Z.; Mann, A. K. P.; Li, M.; Overbury, S. H. Spectroscopic Investigation of Surface-Dependent Acid–Base Property of Ceria Nanoshapes. *J. Phys. Chem. C* **2015**, *119* (13), 7340–7350.

(25) Ravel, B.; Newville, M. ATHENA , ARTEMIS , HEPHAESTUS : Data Analysis for X-Ray Absorption Spectroscopy Using IFEFFIT. *J. Synchrotron Radiat.* **2005**, *12* (4), 537–541.

(26) Wang, S.-G.; Cao, D.-B.; Li, Y.-W.; Wang, J.; Jiao, H. Chemisorption of CO 2 on Nickel Surfaces. *J. Phys. Chem. B* **2005**, *109* (40), 18956–18963.
